# Hydroxylation of the NOTCH1 intracellular domain regulates Notch signaling dynamics

**DOI:** 10.1038/s41419-022-05052-9

**Published:** 2022-07-12

**Authors:** Francesca Ferrante, Benedetto Daniele Giaimo, Tobias Friedrich, Toshiya Sugino, Daniel Mertens, Sabrina Kugler, Bernd Martin Gahr, Steffen Just, Leiling Pan, Marek Bartkuhn, Michael Potente, Franz Oswald, Tilman Borggrefe

**Affiliations:** 1 Institute of Biochemistry, University of Giessen, Friedrichstrasse 24, 35392 Giessen, Germany; 2 Biomedical Informatics and Systems Medicine, Science Unit for Basic and Clinical Medicine, Aulweg 128, 35392 Giessen, Germany; 3 Max Planck Institute for Heart and Lung Research, Angiogenesis and Metabolism Laboratory, Ludwigstr. 43, 61231 Bad Nauheim, Germany; 4 University Medical Center Ulm, Center for Internal Medicine, Department of Internal Medicine III, Albert-Einstein-Allee 23, 89081 Ulm, Germany; 5 German Cancer Research Center (DKFZ), Bridging Group Mechanisms of Leukemogenesis, B061, Im Neuenheimer Feld 280, 69120 Heidelberg, Germany; 6 University Medical Center Ulm, Center for Internal Medicine, Molecular Cardiology, Department of Internal Medicine II, Albert-Einstein-Allee 23, 89081 Ulm, Germany; 7 University Medical Center Ulm, Center for Internal Medicine, Department of Internal Medicine I, Albert-Einstein-Allee 23, 89081 Ulm, Germany; 8 Institute for Lung Health (ILH), Aulweg 132, 35392 Giessen, Germany; 9 Berlin Institute of Health (BIH) at Charité-Universitätsmedizin Berlin, Berlin, Germany; 10 Max Delbrück Center for Molecular Medicine in the Helmholtz Association (MDC), 13125 Berlin, Germany

**Keywords:** Ubiquitylation, Enzymes

## Abstract

Notch signaling plays a pivotal role in the development and, when dysregulated, it contributes to tumorigenesis. The amplitude and duration of the Notch response depend on the posttranslational modifications (PTMs) of the activated NOTCH receptor – the NOTCH intracellular domain (NICD). In normoxic conditions, the hydroxylase FIH (factor inhibiting HIF) catalyzes the hydroxylation of two asparagine residues of the NICD. Here, we investigate how Notch-dependent gene transcription is regulated by hypoxia in progenitor T cells. We show that the majority of Notch target genes are downregulated upon hypoxia. Using a hydroxyl-specific NOTCH1 antibody we demonstrate that FIH-mediated NICD1 hydroxylation is reduced upon hypoxia or treatment with the hydroxylase inhibitor dimethyloxalylglycine (DMOG). We find that a hydroxylation-resistant NICD1 mutant is functionally impaired and more ubiquitinated. Interestingly, we also observe that the NICD1-deubiquitinating enzyme USP10 is downregulated upon hypoxia. Moreover, the interaction between the hydroxylation-defective NICD1 mutant and USP10 is significantly reduced compared to the NICD1 wild-type counterpart. Together, our data suggest that FIH hydroxylates NICD1 in normoxic conditions, leading to the recruitment of USP10 and subsequent NICD1 deubiquitination and stabilization. In hypoxia, this regulatory loop is disrupted, causing a dampened Notch response.

## Introduction

The highly conserved Notch signaling pathway regulates a wide range of biological processes such as immune cell development and function [1] vascular morphogenesis [2, 3] and its deregulation is frequently observed in cancer [4–6]. Notch signaling is activated by the binding of a Notch ligand to a transmembrane NOTCH receptor. This interaction results in two sequential proteolytic cleavages that lead to the release of the NOTCH intracellular domain (NICD). The NICD subsequently translocates into the nucleus where it interacts with the transcription factor (TF) RBPJ, the coactivator MAML1 (MASTERMIND-LIKE 1), and the acetyltransferase EP300 to drive the expression of Notch target genes [7]. This transcriptional program is regulated by posttranslational modifications (PTMs) of the NICD, including prolyl isomerization [8–10], influencing the amplitude and duration of the Notch response [11, 12].

Cells have developed mechanisms to cope with oxygen (O_2_) deprivation (hypoxia) by activating the hypoxia-induced factors (HIFs) [13, 14]. Under normoxic conditions, two different 2-oxoglutarate-dependent oxygenases regulate HIF1α: Prolyl hydroxylases (PHDs), that catalyze prolyl (P) hydroxylation of HIF1α, and factor inhibiting HIF (FIH), which catalyzes of asparagine (N) hydroxylation [15, 16]. Proline hydroxylation of HIF1α promotes its proteasomal degradation by the Von-Hippel-Lindau (VHL)-containing E3 ubiquitin ligase complex [13, 14, 17]. FIH-mediated asparagine hydroxylation in the C-terminal activation domain of HIF1α prevents its interaction with the coactivator EP300 [16]. Limited availability of O_2_ leads to inhibition of both PHDs and FIH resulting in the stability of HIF1α (and of other members of the same family of transcription factors) and activation of the hypoxia-inducible target genes [13, 14, 16, 17]. Interestingly, FIH has a higher O_2_ affinity and still functions under intermediate O_2_ levels [18]. In addition, FIH seems to be physiologically important in situations with a rapid onset of hypoxia such as ischemia [19].

Given the central importance of Notch and hypoxia pathways not only for development but also for homeostasis, it is not surprising that both signaling cascades regulate each other. For instance, hypoxia has been reported to induce the expression of direct Notch target genes of the Hairy Enhancer of the Split family [20–28], suggesting that hypoxia increases the Notch pathway activity. Moreover, FIH has been shown to hydroxylate NICD within the ankyrin (ANK) repeats [12, 27, 29–31], providing a potential molecular mechanism for the direct oxygen-dependent regulation of Notch signaling. However, the functional consequences of NICD hydroxylation are not entirely clear.

Here, we study the consequences of hypoxia on Notch signaling using a mouse progenitor T-cell line, in which the Notch pathway is constitutively active. Unexpectedly, we observe that the majority of Notch target genes are downregulated in conditions of hypoxia correlating with lower NICD1 hydroxylation and protein levels. Using an antibody recognizing site-specific NICD1 hydroxylation, we find that FIH mediates NICD1 hydroxylation. We further show that NICD1 hydroxylation alters its ubiquitination, influencing both degradative and non-degradative ubiquitin chains. Moreover, we observe that the molecular crosstalk between NICD1 hydroxylation and ubiquitination depends on deubiquitinase (DUB) USP10, which modulates Notch responses.

## Materials and methods

### Generation of the NICD1 N1945-OH antibody

Antibody was raised against the hydroxylated asparagine 1945 (N*) of the NICD1 sequence ASADA N* IQDNM and affinity purified with peptides immobilized on sulfolink beads. The serum was first passed over a column with NICD1 N1945-OH peptides; afterward, unspecific antibody was depleted over a column with unmodified NICD1 peptides. The supernatant containing the NICD1 N1945-OH antibody was recovered and dialyzed overnight in PBS. The specificity of the purified antibody was analyzed by dot blot. Peptides were synthesized at Biosynthan and the antibody was produced by BioGenes.

## Results

### Hypoxia or DMOG-treatment downregulates expression of Notch target genes

We studied the molecular interplay between Notch signaling and hypoxia using the mouse progenitor T-cell line Beko, derived from T-cell receptor knockout mice, in which the Notch pathway is constitutively active [32–34]. We treated Beko cells for 24 hours with the hydroxylase inhibitor dimethyloxalylglycine (DMOG) or kept the cells for 12 h under hypoxic conditions (1% O_2_). We observed increased stability of both HIF1α and HIF2α proteins (Fig. S1A, B). In addition, both regimens elicited similar transcriptional responses: 780 genes were affected in both conditions (Fig. S1C–G and Tables S1, S2). Gene set enrichment analysis using KEGG datasets showed that genes associated with the “HIF1 signaling pathway” were significantly enriched in the genes commonly regulated by DMOG and hypoxia (Fig. S1H and Table S3). Similarly, “response to hypoxia” and “cellular response to hypoxia” were among the significantly enriched gene ontology (GO) terms of the overlap of upregulated genes (Table S4). Gene set enrichment analysis (GSEA) also demonstrated that genes linked to “response to hypoxia” were enriched (Tables S5, S6). The upregulation of hypoxia target genes was further validated by qPCR (Fig. S1I, J). The GSEA analysis also unveiled that genes associated with the “Notch signaling pathway” were significantly changed upon both DMOG and hypoxia treatment (Fig. S2A, B and Tables S5,S6).

**Fig. 1:**
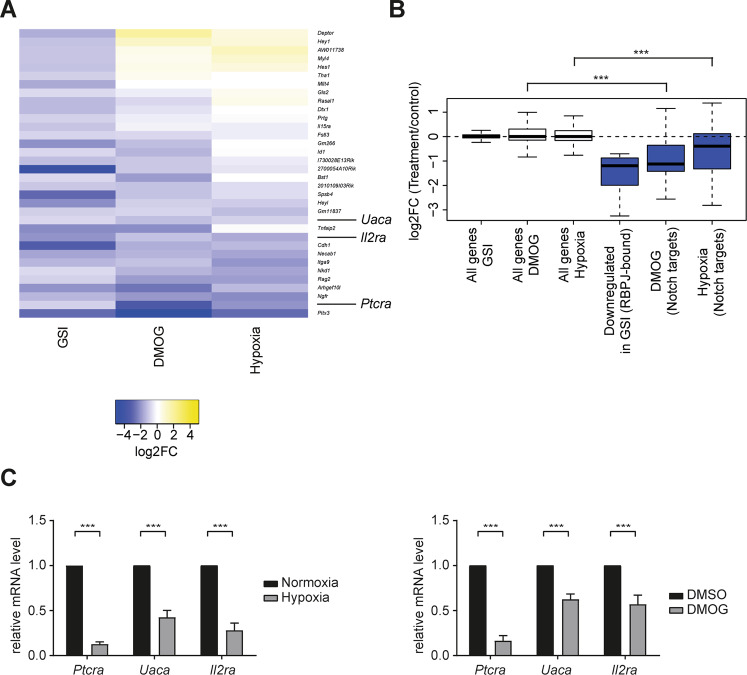
Hypoxia downregulates the Notch-dependent gene expression program. Beko cells were treated with 10 μg/mL GSI [32], 0.5 mM DMOG, or with DMSO as a control for 24 h. Alternatively, cells were kept for 12 h in hypoxia (1% O_2_) or normoxia (5% O_2_) as a control. RNA was purified and analyzed by deep sequencing or qPCR. **A** Heat map showing the effect of DMOG treatment (DMOG vs DMSO) or hypoxia (hypoxia vs normoxia) on the expression of bona fide Notch target genes, defined as those genes that are significantly downregulated by GSI (GSI vs DMSO; log2FC < −1 and adjusted *p* value <0.05) and associated with an RBPJ binding site. **B** Box plot showing the quantification of the heat map shown in panel **A**. Bona fide Notch target genes are significantly downregulated by DMOG (DMOG vs DMSO) or hypoxia (hypoxia vs normoxia). Wilcoxon rank-sum tests (***P < 0.001, NS not significant). **C** Validation of the RNA-Seq experiments by qPCR. *Ptcra*, *Uaca*, and *Il2ra* (encoding for CD25) are downregulated upon (left) hypoxia or (right) DMOG treatment in Beko cells. Data were normalized to the housekeeping gene *Hypoxanthine Guanine Phosphoribosyltransferase* (*Hprt*). Shown is the mean ± SD of five independent experiments (*N* = 5; [***] *P* < 0.001, unpaired Student’s *t*-test).

To further investigate how Notch signaling is regulated by hypoxia in these cells, we made use of RNA- and ChIP-Seq datasets in Beko cells, in which Notch signaling is inhibited by the γ-secretase inhibitor (GSI) DAPT [32]. We focused on genes that were downregulated by GSI and bound by the transcriptional effector RBPJ, assuming that these are direct Notch target genes. Using this approach, we defined 34 genes as bona fide direct Notch targets, including *Hes1* and *Hey1* encodes for transcriptional repressors, *Il2ra* (IL2 receptor-alpha, also known as CD25) that encodes for a subunit of the IL2 receptor, which is required for cell-autocrine T-cell homeostasis and *Ptcra* (preT-cell receptor-alpha) which encodes for the essential subunit of the preT-cell receptor (preTCR) (Fig. 1A, B and Table S7). Taking advantage of this Notch signature, we observed that canonical Notch targets *Hes1* and *Hey1* are upregulated in hypoxic conditions, which is in line with previous reports [12, 20–28]. However, surprisingly, we observed that the majority of the Notch signature genes are downregulated upon hypoxia or DMOG treatment (Fig. 1A, B and Table S2), a finding that was also validated by qPCR (Figs. 1C and S2C, D). To better understand whether the transcriptional regulation is RBPJ/Notch-dependent or HIF1α dependent, we performed ChiP-Seq experiments in Beko cells to determine the localization of transcription factors RBPJ and HIF1α in normoxic and hypoxic conditions (Table S7). We observed a significant increase in the binding of HIF1α upon hypoxia (Fig. S3A) but the genomic binding of RBPJ was hardly influenced (Fig. S3B). The hypoxia-responsive element (HRE) was promptly identified at the HIF1α binding sites (Fig. S3C) and we could identify several genes that are deregulated by hypoxia or DMOG and bound by HIF1α (Fig. S3D and Table S8). When focusing on the bona fide Notch target genes, we observed that both *Hes1* and *Hey1* are bound by HIF1α upon hypoxia while the binding of RBPJ is reduced, suggesting that their upregulation is HIF1α dependent (Fig. S3E, F).

Together, our data reveal that in the Notch-ON state the majority of Notch target genes are downregulated upon hypoxia. In contrast, Notch targets like *Hes1* and *Hey1*, which are also true HIF1α targets, are upregulated upon hypoxia.

### FIH positively regulates the NICD1 protein stability

**Fig. 2:**
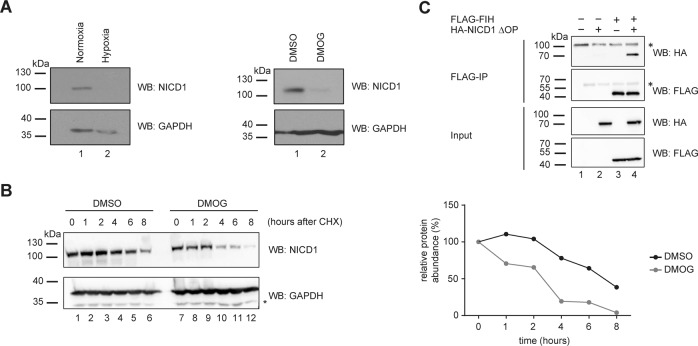
Hypoxia destabilizes the active cleaved NICD1 protein. **A** Beko cells were kept for (left) 12 h in hypoxia (1% O_2_) or normoxia (5% O_2_) as control or, alternatively, (right) treated with 0.5 mM DMOG or with DMSO as a control for 24 h. Whole-cell extracts (WCE) were analyzed by Western blotting (WB) versus the endogenous cleaved NICD1 protein or GAPDH as a loading control. **B** DMOG treatment destabilizes the NICD1 protein. Beko cells were treated for 24 h with 0.5 mM DMOG or DMSO as control and, after the first 16 h, protein synthesis was blocked by adding 50 µg/mL cycloheximide (CHX). Samples were collected at the indicated time points. WCE was analyzed by WB versus endogenous cleaved NICD1 or GAPDH as a loading control. Quantification of the NICD1 levels normalized to GAPDH is shown on the right. The experiment was repeated independently three times. **C** FIH and NICD1 ΔOP interact with each other. Phoenix^TM^ cells were transfected with plasmids encoding HA-tagged NICD1 deleted of the OPA and PEST domains (ΔOP) and/or FLAG-tagged FIH. WCE were subjected to FLAG immunoprecipitation (FLAG-IP) and the immunoprecipitates were analyzed by WB versus FLAG or HA.

PTMs of the NICD1 regulate its stability and activity [11, 12]. Given that FIH is an O_2_-sensitive enzyme known to hydroxylate NICD1 [12, 27, 29–31], we reasoned that FIH might influence NICD1’s stability. Indeed, we observed lower NICD1 protein levels after 12 h of hypoxia incubation or 24 h of DMOG treatment (Fig. 2A) in Beko cells, consistent with the downregulation of the Notch target gene signature (Figs. 1A–C, S2C, D, and Table S2). To determine whether these effects are due to changes in NICD1 protein half-life, we performed cycloheximide (CHX) experiments in Beko cells (Figs. 2B and S4H). We observed that DMOG treatment reduces the stability of NICD1 compared to the DMSO treated controls (Figs. 2B and S4H). Given that DMOG inhibits both FIH and PHDs [35–39], we treated Beko cells with roxadustat, a selective inhibitor of PHDs. We observed that roxadustat did not affect NICD1 protein levels (Fig. S4A), suggesting that the DMOG- and hypoxia-induced effects on Notch signaling are caused by FIH. In support of a functional relationship between FIH and NICD1, we found that these proteins interact with each other in both HEK293 cells (Fig. 2C) and in Beko cells (Fig. S4B, C). We further mapped the domains necessary for the FIH/NICD1 interaction and found that it requires the ankyrin-repeats of the NICD1 (Fig. S4D, E). Finally, using NICD1 and FIH ChIP we demonstrate in Beko cells that both proteins co-occupy enhancers of Notch target genes that are downregulated in hypoxic conditions (Fig. S4F, G). Altogether, these data suggest that FIH stabilizes the NICD1.

### Characterization of the FIH-mediated asparaginyl hydroxylation of the NICD1

**Fig. 3:**
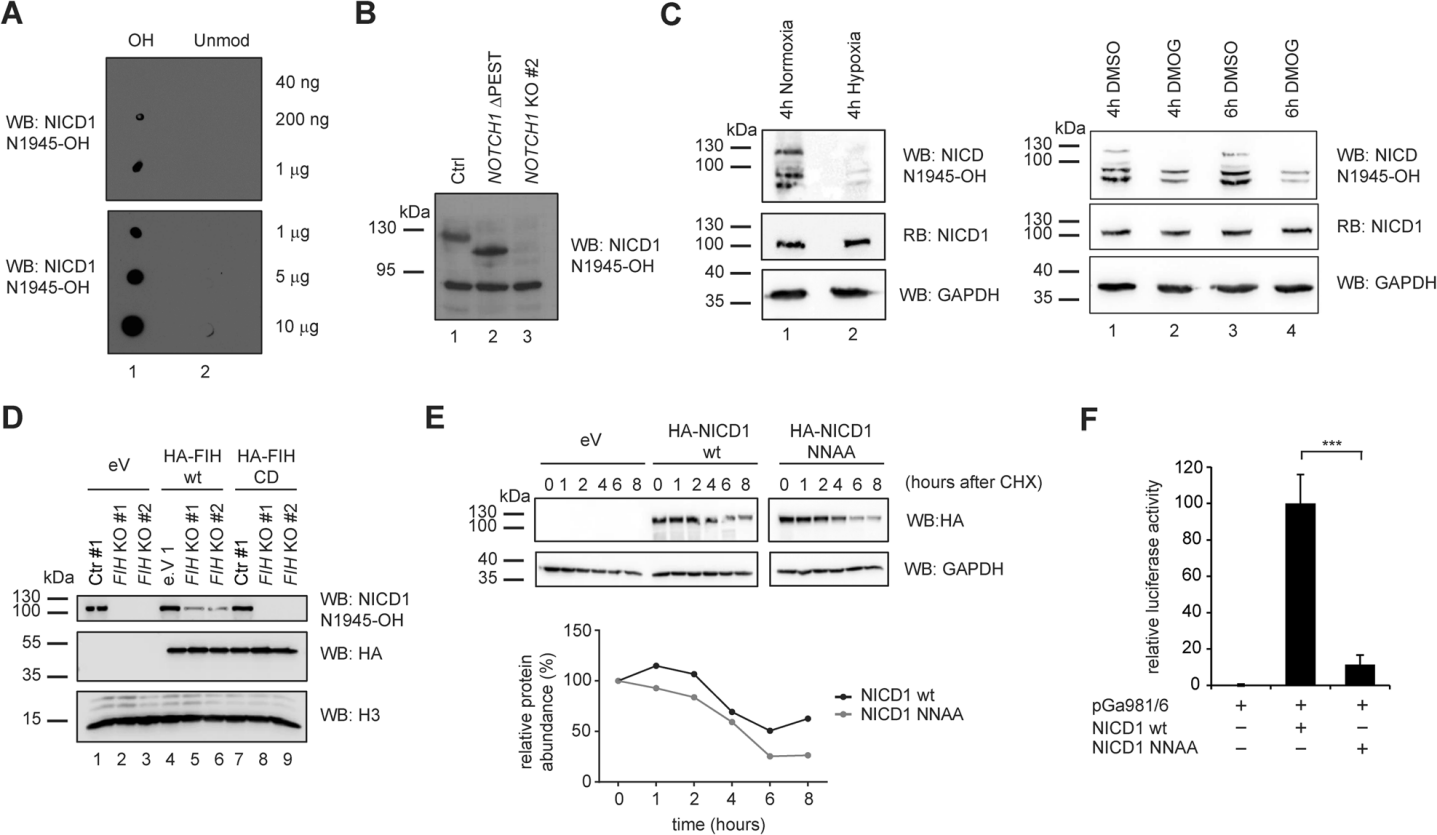
A hydroxylation-resistant NICD1 mutant has reduced transcriptional activity. **A** Dot blot showing the specificity of the antibody against NICD1 hydroxylated on N1945 (NICD1 N1945-OH). The indicated amounts of NICD1 N1945-OH or unmodified NICD1 N1945 peptides were pipetted on a nitrocellulose membrane. Membranes were incubated with the NICD1 N1945-OH antibody. **B** The NICD1 N1945-OH antibody specifically recognizes the NICD1 in HEK 293 cells. Whole-cell extracts (WCE) from HEK 293 cells wild-type (wt), depleted of the *NOTCH1* gene (*NOTCH1* KO #2) or of only the sequence encoding for its C-terminal PEST domain (*NOTCH1* ΔPEST) were analyzed by Western blotting (WB) versus NICD1 N1945-OH. **C** The NICD1 N1945-OH antibody specifically recognizes the NICD1 in Beko cells. Beko cells were kept for (left) 4 h in hypoxia (1% O_2_) or normoxia (5% O_2_) as control or, alternatively, treated with (right) 0.5 mM DMOG or with DMSO as a control for 4 or 6 h. WCE were analyzed by WB versus the endogenous NICD1 N1945-OH and reblotted (RB) versus the cleaved NICD1 protein or GAPDH as a loading control. **D** FIH hydroxylates NICD1 on N1945. HeLa cells depleted of FIH were transfected with plasmids encoding for HA-tagged FIH wild-type (wt), HA-tagged catalytic dead (CD) FIH mutant, or an empty vector as a control (eV). WCE were analyzed by WB versus NICD1 N1945-OH, HA or H3 as a loading control. **E** NICD1 wild-type is more stable compared to the NICD1 NNAA mutant. Phoenix^TM^ cells were transfected with plasmids encoding for HA-tagged NICD1 wild-type (wt), HA-tagged NICD1 NNAA mutant or an empty vector as a control (eV). Protein synthesis was blocked by adding 150 µg/mL cycloheximide (CHX) and samples were collected at the indicated time points. WCE were analyzed by WB versus HA or GAPDH as a loading control. Quantification of the NICD1 levels normalized to GAPDH is shown below. The experiment was repeated independently three times. **F** NICD1 wildtype is more active compared to the NICD1 NNAA mutant. Transactivation capacities of NICD1 wildtype (wt) or NICD1 NNAA mutant was tested in luciferase assays using the RBPJ-dependent reporter construct pGA891/6 in HeLa cells. Mean values ± SD (error bars) from four independent experiments are shown (*N* = 4; [***] *p* < 0.0001, unpaired Student’s *t*-test).

To further understand the regulation of Notch signaling by the FIH-mediated hydroxylation of NICD1, we generated an antibody recognizing hydroxylated NICD1 on N1945 (NICD1 N1945-OH) but not the unmodified protein (Fig. 3A). To test the specificity of the antibody, we generated HEK293 cells lacking NOTCH1 or just its C-terminal PEST domain important for the ubiquitin-mediated degradation of NICD1 (ΔPEST; Fig. S5A). Western blotting for NICD1 validated the correct targeting of HEK293 cells showing the truncated protein in the NICD1 ΔPEST cells (Fig. S5A, compare lane 2 to lane 1) but no detectable signal in the *NOTCH1* knockout cells (Fig. S5A, compare lanes 3 and 4 to lane 1). Similar results were obtained for the N1945-OH-specific antibody (Fig. 3B, compare lane 2 to lane 1 and lane 3 to lane 1). Importantly, the NICD1 N1945-OH antibody failed to recognize the NICD1 protein when both the NICD1 hydroxylation sites N1945 or N2012 were mutated to alanine residues (NNAA; Fig. S5B lane 3) or when only the N1945 was mutated to alanine (N1945A; Fig. S5B lane 4). In line with these results, our antibody recognizes a NICD1 mutated only on N2012 (N2012A; Fig. S5B lane 5). Altogether, these data validate the specificity of our NICD1 N1945-OH antibody.

Using this tool, we observed that hypoxia or DMOG but not 24 h of roxadustat treatment lead to a reduction of asparaginyl-hydroxylated NICD1 in Beko cells (Fig. S5C–E). To exclude that the reduction of modified NICD1 just reflects a reduction in overall NICD1 protein abundance, we shortened the hypoxia incubation and DMOG treatment. We kept Beko cells for 4 hours under hypoxia or we treated the cells for 4 or 6 h with DMOG. Under these conditions, NICD1 protein levels remained unchanged (Fig. 3C, middle panels) while the levels of N1945 hydroxylated NICD1 were diminished (Fig. 3C, upper panels). These data demonstrate that the reduction in NICD1 hydroxylation precedes the reduction in protein levels and raises the possibility that hypoxia reduces NICD1 stability via its altered hydroxylation.

Similarly to Beko cells, DMOG treatment or hypoxia in RPMI-8402, a human T-cell acute lymphoblastic leukemia (T-ALL) line, again results in downregulation of Notch target genes (Fig. S6A, B) associated with decreased NICD1 protein level (S6C, D) and reduced NICD1 hydroxylation (Fig. S6E, F).

To assess whether FIH is implicated in this regulation, we generated cells depleted for FIH using CRISPR/Cas9 (Fig. S7A–D). In FIH-depleted HeLa cells, NICD1 hydroxylation on N1945 was abolished (Fig. 3D, compare lanes 2 and 3 to lane 1). Furthermore, re-expression of wild-type (wt) but not catalytically dead (CD) FIH [40] restored NICD1 hydroxylation (Fig. 3D). In addition, we also analyzed NICD1 mutants harboring mutations of the hydroxylation acceptor sites N1945 and N2012 (Fig. S7E). Overexpression of FIH increases NICD1 N1945-OH when the NICD1 wild-type or N2012A mutant but not NICD1 N1945A or NICD1 NNAA mutants are co-overexpressed (Fig. S7E). Biochemically, the protein stability of the NICD1 NNAA mutant is slightly reduced as shown in CHX assays (Fig. 3E). Importantly, the mutation of N1945A and N2012A did not impact NICD1’s interaction with RBPJ and MAML1 in HEK293 cells (Fig. S8A–D). Similar to the wild-type NICD1, the mutant also localizes to the nucleus in HeLa cells (Fig. S8E). In contrast, transactivation of Notch-dependent luciferase reporters was severely compromised (Fig. 3F). Altogether, these data suggest that the hydroxylation-resistant NICD1 mutant is less stable and less transcriptionally active.

**Fig. 4:**
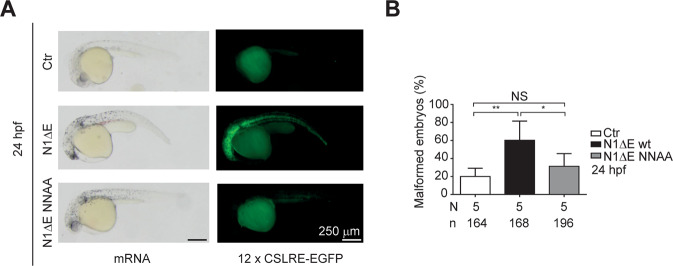
Hydroxylation-resistant NICD1 is functionally less active. **A** The NICD1 NNAA mutant is less active compared to the NICD1 wild-type in zebrafish. Zebrafish embryos were injected with mRNA encoding for the membrane-bound Notch1ΔE wild-type (N1ΔE wt) or NNAA mutant (N1ΔE NNAA). A reporter plasmid where the GFP-encoding gene is under the control of a Notch-dependent promoter was co-injected to monitor the NICD1 activity. **B** Quantification of malformed embryos shown in panel **A**. Shown are the means ± SD of the total number of embryos analyzed (*n*) in five independent experiments (*N* = 5). [*] *P* < 0.05, [**] *P* < 0.01, [NS] not significant (nonparametric Mann–Whitney *U*-test).

To further explore the function of hydroxylation-resistant NICD1 in vivo, we tested the NICD1 NNAA mutant in embryonic development using *Danio rerio* (zebrafish) as well known Notch model system (Fig. 4A, B). We injected mRNA encoding for N1ΔE wt or NNAA mutant in one-cell-stage zebrafish embryos together with a Notch-dependent GFP reporter plasmid (12x CSLRE-EGFP). In control (vector only) embryos Notch-driven gene expression is on background levels. Wild-type (wt) N1ΔE but not the NNAA mutant is able to drive the expression of the Notch reporter (EGFP) (Fig. 4A). In line with this, the number of malformed embryos was much higher in presence of the N1ΔE wt compared to the hydroxylation-deficient NICD1 NNAA mutant (Fig. 4B). Together, these data support the notion that the hydroxylation-defective NICD1 is functionally impaired in vivo in Notch-dependent neurogenesis in *D. rerio*.

### FIH-mediated NICD1 hydroxylation regulates its stability using a ubiquitination-dependent mechanism

**Fig. 5:**
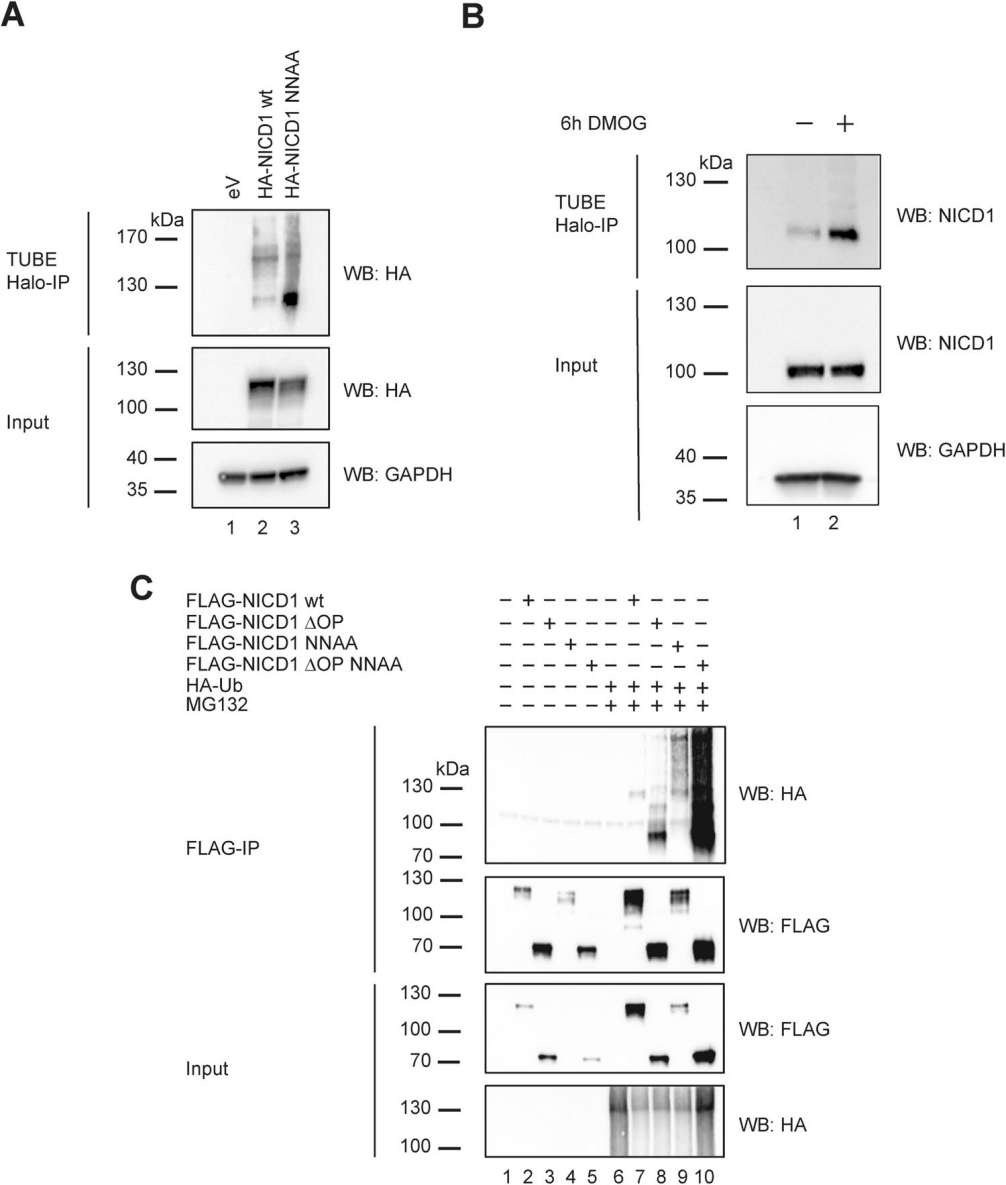
Hypoxia increases NICD1 ubiquitination. **A** NICD1 NNAA mutant is more ubiquitinated compared to the NICD1 wild-type. Phoenix^TM^ cells were transfected with plasmids encoding for HA-tagged NICD1 wild-type (wt), HA-tagged NICD1 NNAA mutant, or an empty vector as a control (eV). After 6 h of treatment with 20 mM of MG132, protein extracts were prepared and subjected to immunoprecipitation (IP) by performing a TUBE assay. Immunoprecipitates were analyzed by Western blotting (WB) versus HA or GAPDH as a loading control. **B** DMOG treatment enhances NICD1 ubiquitination in Beko cells. Beko cells were treated for 24 h with 0.5 mM DMOG or DMSO as a control. After 6 h of treatment with 20 mM of MG132, protein extracts were prepared and subjected to immunoprecipitation (IP) by performing a TUBE assay. Immunoprecipitates were analyzed by WB versus NICD1 or GAPDH as a loading control. **C** The increased ubiquitination of the NICD1 NNAA mutant does not require the C-terminal PEST domain. Phoenix^TM^ cells were transfected with plasmids encoding for FLAG-tagged NICD1 wild-type (wt), FLAG-tagged NICD1 deleted of the OPA and PEST domain (ΔOP), FLAG-tagged NICD1 NNAA mutant, FLAG-tagged NICD1 ΔOP NNAA mutant and/or HA-tagged ubiquitin (HA-Ub). Cells were treated with 20 mM of MG132 for 6 h to block the activity of the proteasome. WCE were subjected to FLAG immunoprecipitation (FLAG-IP) and the immunoprecipitates were analyzed by WB versus FLAG or HA.

Ubiquitination of NICD1 is pivotal to limit Notch responses and is guided by other NICD1 PTMs [11, 32, 33, 41, 42]. Therefore, we investigated whether NICD1 hydroxylation is coupled to ubiquitination. To this end, we used tandem ubiquitin-binding entities (TUBEs) assays [43] to assess ubiquitination patterns of wild-type and NNAA-mutated NICD1. We observed that the NICD1 NNAA mutant shows increased ubiquitination compared to its wild-type counterpart in *Phoenix*^TM^ cells (Fig. 5A, compare lane 3 to 2). Similarly, we observed an increase in ubiquitination of the endogenous NICD1 upon DMOG treatment of Beko cells (Fig. 5B). Interestingly, the NICD1 ubiquitination was also increased in the NICD1 NNAA mutants that lack the destabilizing OPA-PEST domain in *Phoenix*^TM^ cells (Fig. 5C, compare lane 7 with 9 and lane 8 with 10), suggesting that there is additional ubiquitination beside of degradative K48-linked ubiquitination that is known to occur within the PEST domain [44, 45]. While K48-linked ubiquitination plays key roles in proteasomal degradation, ubiquitination through K11 or K63 has important signaling functions [46]. To further unravel the link between NICD1 hydroxylation and ubiquitination, we used ubiquitin (Ub) mutants in which specific lysines were mutated to arginines (K11R, K48R or K63R) or mutants in which all the lysines with the exception of a single one were mutated to arginines (K11 only, K48 only and K63 only). In *Phoenix*^TM^ cells, we observed that the increased ubiquitination of the NICD1 NNAA mutant in comparison to the NICD1 wt was abolished with the K63R Ub mutant (Fig. S9A, compare lane 13 with 12) and slightly reduced with the K11R Ub mutant (Fig. S9A, compare lane 15 with 12) but hardly influenced in presence of the K48R mutant (Fig. S9A, compare lane 14 with 12). In addition, in presence of Ub K63 only, the ubiquitination of the NICD1 NNAA was retained even if slightly reduced (Fig. S9B, compare lane 13 with 12). In presence of the Ub K48 only, the ubiquitination of the NICD1 NNAA mutant was almost completely abolished (Fig. S9B, compare lane 14 with 12) even if still higher than the one of the NICD1 wt in presence of the Ub K48 only (Fig. S9B, compare lane 14 with 10). In presence of the Ub K11 only, the ubiquitination of the NICD1 NNAA mutant was strongly reduced (Fig. S9B, compare lane 15 with 12) but higher than the one of the NICD1 wt in presence of the Ub K11 only (Fig. S9B, compare lane 15 with 11). Altogether, these data suggest that NICD1 hydroxylation prevents its ubiquitination and that the increased ubiquitination of the NICD1 protein is mainly occurring through Ub K63 and to a minor but still significant extent to Ub K11 while there is a minimal increase through Ub K48.

### The interplay between NICD1 hydroxylation and ubiquitination involves the deubiquitinase USP10

To further explore the link between NICD1 hydroxylation and ubiquitination, we focused on the E3 ubiquitin ligase FBXW7, which targets NICD1 for proteasomal degradation [42, 44]. We hypothesized that NICD1 hydroxylation affects the NICD1/FBXW7 interaction and hence impairs NICD1 ubiquitination. We performed co-immunoprecipitation experiments in *Phoenix*^TM^ cells co-expressing FBXW7 as well as Flag-tagged NICD1 wild-type (wt) or NNAA mutant (Fig. S10A). We observed no major changes in the interaction between FBXW7 and NICD1 NNAA when compared to the NICD1 wild-type (Fig. S10A, compare lane 5 with 6), suggesting that the increased NICD1 ubiquitination is not due to enhanced FBXW7 recruitment.

**Fig. 6:**
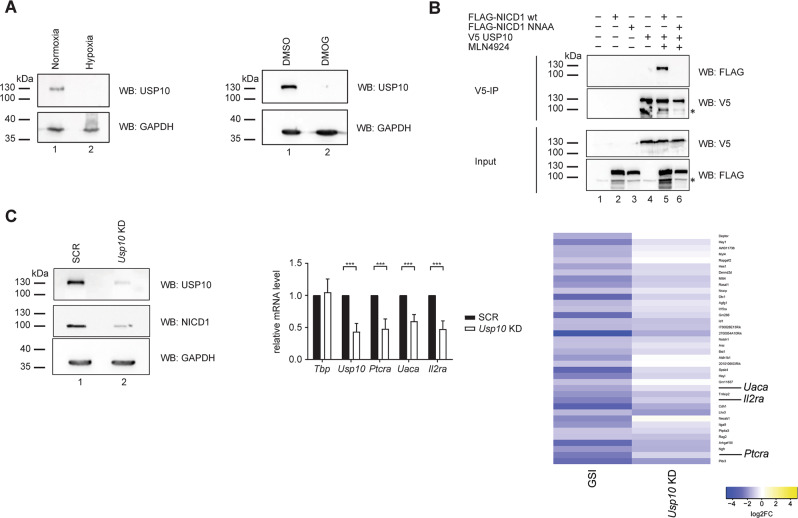
USP10 is downregulated upon hypoxia induction, weakly interacts with the NICD1 NNAA mutant and its depletion leads to reduced expression of Notch target genes. **A** USP10 is downregulated upon hypoxia induction. Beko cells were kept for (left) 12 h in hypoxia (1% O_2_) or normoxia (5% O_2_) as control or, alternatively, (right) treated with 0.5 mM DMOG or with DMSO as a control for 24 h. Whole-cell extracts (WCE) were analyzed by Western blotting (WB) versus USP10 protein or GAPDH as a loading control. **B** USP10 weakly interacts with the NICD1 NNAA mutant. Phoenix^TM^ cells were transfected with plasmids encoding FLAG-tagged NICD1 wt (wt), FLAG-tagged NICD1 NNAA mutant, and/or V5-tagged USP10. Thirty-six hours post-transfection, Phoenix^TM^ cells were treated with 10 μM MLN4924 for 3 h. WCE were subjected to V5 immunoprecipitation (V5-IP) and the immunoprecipitates were analyzed by WB versus FLAG or V5. **C** USP10 depletion leads to reduced NICD1 protein levels and downregulation of Notch target genes. Beko cells were infected with shRNAs directed against *Usp10* (*Usp10 KD*) or scramble (SCR) as a control. (Left) Whole-cell extracts (WCE) were analyzed by Western blotting (WB) versus USP10 protein, NICD1, or GAPDH as a loading control. (Middle) Upon RNA extraction and reverse transcription, cDNAs were analyzed by qPCR using primers specific for *Tbp*, Usp10, *Ptcra*, *Uaca*, or *Il2ra*. Data were normalized to the housekeeping gene *Hypoxanthine Guanine Phosphoribosyltransferase* (*Hprt*). Shown is the mean ± SD of three independent experiments measured twice each (*N* = 6; [***] *P* < 0.001, unpaired Student’s *t*-test). (Right) Heat map showing the effects of *Usp10 KD* on the bona fide Notch target genes defined as downregulated upon GSI treatment and bound by RBPJ.

Protein ubiquitination is reversed by deubiquitinases (DUBs), which cleave ubiquitin (chains) from their substrates. Recently, USP10 has been shown to regulate NICD1 ubiquitination [47]. Of note, we found in our RNA-Seq analysis that the expression of the USP10-encoding gene is downregulated in hypoxic or DMOG-treated Beko cells (Table S2). Similar results were obtained at the protein level in Beko cells (Fig. 6A). However, no expression changes were noted for FBXW7 in Beko cells (Fig. S10B, C). Furthermore, we observed that USP10 interacted less efficiently with NICD1 NNAA than with the NICD1 wild-type in *Phoenix*^TM^ cells (Fig. 6B, compare lanes 5 to 6), suggesting that the hypoxia-induced changes in NICD1 ubiquitination and stability result from impaired deubiquitination. These data suggest a model, whereby FIH-mediated hydroxylation determines the ability of NICD1 to interact with USP10, a DUB, whose expression is downregulated by hypoxia. In addition, we observed that *Usp10* knockdown in Beko cells leads to reduced NICD1 protein levels (Fig. 6C left) and to reduced expression of *Ptcra*, *Uaca*, and *Il2ra* Notch target genes (Fig. 6C middle). In line with that, GSEA analysis upon *Usp10* knockdown in Beko cells followed by RNA-Seq (Fig. S11A and Tables S1,S2) demonstrated that genes linked to the “Notch signaling pathway” were enriched (Fig. S11B and Table S9). In addition, a general downregulation of the Notch response was observed when looking at bona fide Notch target genes in our RNA-Seq analysis (Fig. 6C right, S11C, and Tables S1,S2).

Together, our data strongly suggest that USP10 stabilizes NICD1 in Beko cells through a hydroxylation-sensitive mechanism.

## Discussions

Regarding the interplay between Notch signaling and hypoxia, we have uncovered that downregulation of Notch target genes is due to reduced NICD1 protein stability. In normoxic conditions, FIH hydroxylates the NICD1 affecting ubiquitination regulated by E3 ubiquitin ligase FBXW7 and deubiquitinase USP10 (see also model in Fig. S12). Upon hypoxia, NICD1 hydroxylation ceases and the USP10 protein level is reduced, leading to perturbed NICD1 ubiquitination and subsequent downregulation of Notch target genes.

Seemingly contradictory to our data, several other studies have suggested hypoxia as an inducer of the Notch signaling pathway [20–28]. However, these studies focused only on the Hairy Enhancer of Split family of genes as a readout of Notch activation, which have later been shown to be also regulated by HIF1α and other signaling pathways [12, 26, 28]. For example, it was shown that mutations of the RBPJ binding motif do not prevent the hypoxia-mediated induction of *Hes1* [28], supporting the notion that this upregulation is Notch-independent. In line with this, we observe the binding of HIF1α upon hypoxia at *Hes1* and *Hey1*. Thus, taking a genome-wide approach we disentangle RBPJ/NICD from HIF1α-regulated target genes.

Mechanistically, NICD1 protein stability is affected by hypoxia and postulates a link between NICD1 hydroxylation and ubiquitination. We propose that not only the hypoxia-regulated enzyme FIH but also the deubiquitinase USP10 play a key role in this process resulting in a shifted balance to enhanced NICD1 ubiquitination, in particular, regulatory ubiquitination (see also model in Fig. S12). According to our model, NICD1 hydroxylation provides a docking site for USP10, which in turn deubiquitinates NICD1.

It remains to be determined which E3 ubiquitin ligase counteracts USP10. Apart from FBXW7, known to control degradative ubiquitination, possible candidates are ITCH, MDM2, and/or RNF8, that have been previously linked to NOTCH ubiquitination [48–51].

Our data reveal that hypoxia affects not only HIF1α but also the NICD1 coactivator. In our view, this has both physiological and potentially also pathophysiological implications. Physiologically, NOTCH1 is essential in early T-cell development in the thymus [52, 53], which is known to be highly proliferative with low O_2_ tension [54, 55]. In our view, this hypoxic condition could contribute to the modulation of Notch responses, which is pivotal for subsequent T-cell maturation. Our results showing reduced expression of *Ptcra* and *Il2ra* (encoding for CD25) support this hypothesis. Regarding the pathophysiological context, our findings are particularly relevant to Notch-driven leukemias, T-cell acute lymphoblastic leukemia (T-ALL), and chronic lymphocytic leukemia (CLL), for which *NOTCH1* mutations have been previously described [56, 57]. They could also play a role in the setting of solid cancer such as breast cancer and squamous cell carcinoma, where active Notch signaling has been described [4, 9, 58]. Future genome-wide analyses should not only focus on HIF1α but also on Notch-dependent gene regulation.

Together, our data suggest that NICD1 hydroxylation determines not only the strength but also the duration of Notch responses through an FIH- and USP10-dependent mechanism.

## Supplementary information

Supplementary text

Figure S1

Figure S1 continued

Figure S2

Figure S3

Figure S3 continued

Figure S4

Figure S4 continued

Figure S5

Figure S6

Figure S7

Figure S8

Figure S8 continued

Figure S9

Figure S10

Figure S11

Figure S12

Table S1

Table S2

Table S3

Table S4

Table S5

Table S6

Table S7

Table S8

Table S9

original blots

author checklist

## Data Availability

ChIP-Seq and RNA-Seq developed in the current study have been deposited at GEO under the accession number GSE194003. Detailed Materials and Methods are available in the supplement file.
