# Supplementary material for: Hydroxylation of the NOTCH1 intracellular domain regulates Notch signaling dynamics
Source: Cell Death Dis. 2022 Jul 12;13(7):600. doi: 10.1038/s41419-022-05052-9 (PMC9276811; doi:10.1038/s41419-022-05052-9)
Supplement: Supplementary file 1 — Supplementary text [file 41419_2022_5052_MOESM1_ESM.docx]

**Hydroxylation of the NOTCH1 intracellular domain regulates Notch signaling dynamics**

Francesca Ferrante^1^, Benedetto Daniele Giaimo^1^, Tobias Friedrich^1,2^, Toshiya Sugino^3^, Daniel Mertens^4,5^, Sabrina Kugler^4^, Bernd Martin Gahr^6^, Steffen Just^6^, Leiling Pan^7^, Marek Bartkuhn^2,8^, Michael Potente^3,9,10^, Franz Oswald^7^ and Tilman Borggrefe^1,*^

^1^ Institute of Biochemistry, University of Giessen, Friedrichstrasse 24, 35392 Giessen, Germany

^2^ Biomedical Informatics and Systems Medicine, Science Unit for Basic and Clinical Medicine, Aulweg 128, 35392 Giessen, Germany

^3^ Max Planck Institute for Heart and Lung Research, Angiogenesis and Metabolism Laboratory, Ludwigstr. 43, 61231 Bad Nauheim, Germany

^4^ University Medical Center Ulm, Center for Internal Medicine, Department of Internal Medicine III, Albert-Einstein-Allee 23, 89081 Ulm, Germany

^5^ German Cancer Research Center (DKFZ), Bridging Group Mechanisms of Leukemogenesis, B061, Im Neuenheimer Feld 280, 69120 Heidelberg

^6^ University Medical Center Ulm, Center for Internal Medicine, Molecular Cardiology, Department of Internal Medicine II, Albert-Einstein-Allee 23, 89081 Ulm, Germany

^7^ University Medical Center Ulm, Center for Internal Medicine, Department of Internal Medicine I, Albert-Einstein-Allee 23, 89081 Ulm, Germany

^8^ Institute for Lung Health (ILH), Aulweg 132, 35392 Giessen, Germany

^9^ Berlin Institute of Health (BIH) at Charité-Universitätsmedizin Berlin, Berlin, Germany

^10^ Max Delbrück Center for Molecular Medicine in the Helmholtz Association (MDC), 13125 Berlin, Germany

^*^ To whom the correspondence should be addressed. Tel: ++49-(0)641-99-47400; Fax: ++49-(0)641-9947409; Email: [Tilman.Borggrefe@biochemie.med.uni-giessen.de](mailto:Tilman.Borggrefe@biochemie.med.uni-giessen.de)

**Files in this Data supplement:**

Supplemental Materials and Methods

Supplemental references

Supplemental Figure legends 1, 2, 3, 4, 5, 6, 7, 8, 9, 10, 11 and 12

Supplemental Table legends 1, 2, 3, 4, 5, 6, 7, 8 and 9

Supplemental Tables 10 and 11

**MATERIALS AND METHODS**

**Primary antibodies**

The following antibodies were used: α-Tubulin (Sigma-Aldrich T9026), FBXW7 (abcam ab227677), FIH (Cell Signaling 4426), FLAG (Sigma-Aldrich F3165; Sigma-Aldrich F4042), GAPDH (abcam ab8245), GFP (Roche 11814460001), H3 (abcam ab1791), HA (3F10, Roche 1867423), HIF1α (Abcam 228649; Cell Signaling Technology 36169S; Diagenode C15410234), HIF2α (Abcam ab199), His2Av (Active Motif 61686) MAML1 (abcam ab155786), RBPJ (CosmoBio T6709; Cell Signaling 5313), RNAPII (Santa Cruz sc-899), TBP (Santa Cruz sc-273), USP10 (Cell Signaling 5553), V5 (Invitrogen R960-50) and Val1744 cleaved NICD1 (Cell Signaling 2421).

The NICD1 N1945-OH antibody was produced by BioGenes.

**Cell culture, treatments, transfection, infection**

Mouse leukemia progenitor T cells (Beko) were grown in Iscove's Modified Dulbecco Medium (IMDM, Gibco 21980-065) supplemented with 2% fetal bovine serum (Pan Biotech), penicillin/streptomycin (Gibco), nonessential amino acids (Gibco), 0.3 mg/mL Primatone and 5 mg/L insulin (Sigma-Aldrich). 293T cells, Phoenix^TM^ packaging cells (Orbigen, Inc., San Diego, CA, USA) and HeLa cells (ATCC: CCL2) were grown in Dulbecco’s modified eagle medium (DMEM, Gibco 61965-059) supplemented with 10% fetal bovine serum (Pan Biotech) and penicillin/streptomycin (Gibco). RPMI-8402 cells were grown in RPMI1640 medium (Gibco 61870-010) supplemented with 10% fetal bovine serum (Pan Biotech) and penicillin/streptomycin (Gibco). Cells were grown at 37°C under 5% CO_2_.

*Drosophila melanogaster* Schneider cells were grown in Schneider’s *Drosophila* medium (Gibco 21720024) supplemented with 10% fetal bovine serum (Gibco 10270-106), penicillin/streptomycin (Gibco) and Glutamine (Gibco 25030-024).

Cells were treated with 0.5 mM DMOG (StressMarq SIH-382) for 4, 6 or 24 hours, 5 μM MG132 (Calbiochem 474790) for 6 hours, 10 μM MLN4924 (Cayman 15217) for 3 hours, 1 μM or 10 μM Roxadustat (Selleckchem S1007) for 24 hours or 50 µg/mL cycloheximide (Applichem A0879.0001) for specific time points as indicated in the respective figure. As control, the respective vehicles were used. Cells were kept at 1% O_2_ for 4 or 12 hours to induce hypoxia.

Phoenix^TM^ packaging cells were transfected using the calcium phosphate method. Briefly, 5 x 10^6^ cells were seeded in a 75 cm^2^ culture flask in 10 mL medium and incubated at 37°C for 16-24 hours. 20 μg of DNA were diluted in 860 μL of sterile H_2_O. After adding 120 μL of CaCl_2_, the resulting solution was slowly pipetted dropwise into 1 mL of 2 x HBS (50 mM HEPES pH 7.05, 10 mM KCl, 12 mM glucose, 280 mM NaCl, 1.5 mM Na_2_HPO_4_) while vortexing. After 20 min of incubation at room temperature, the DNA solution was added to the cells that were pre-incubated for 10 min with chloroquine (Sigma-Aldrich C6628-100G). 6-8 hours post-transfection, the medium was replaced with fresh one.

Retroviral infections of Beko cells were performed as follows: Phoenix^TM^ cells were transfected as described above and 24 hours post-transfection the supernatant, containing the retroviral particles, was filtered and supplemented with 2 μg/mL of polybrene (Sigma-Aldrich H9268). The viral suspension was used to infect approximately 5 x 10^5^ cells by centrifugation (1.800 rpm, 45 min, 37°C). The infection was repeated 4 times over a period of 2 days. After infection, cells were analyzed and sorted by FACS or selected with specific antibiotics: puromycin (Serva 33835) and/or histidinol (Sigma-Aldrich H6647).

**Lentiviral shRNA knockdown**

*Usp10* knockdown in Beko cells was performed using the pLK0.1 TRC1 shRNA library (Sigma-Aldrich). Briefly, 293T cells were transfected with 3.3 μg of the desired shRNA construct and the packaging vectors psPAX (2.5 μg) and pMD26 (1 μg) using 14 μl of linear PEI (Polyscience 23966). After 48 hours of incubation at 37°C, the supernatant of the 293T cells was filtered and used for infections of Beko cells. The selection of the positively infected cells was performed by adding 1 μg/ml puromycin (Serva 33835). The sequence of the shRNA is available in Table S10.

**Generation of CRISPR/Cas9 depleted cells**

The CRISPR/Cas9 guides were designed using the online tool available at <http://crispor.tefor.net/>. The desired 5’ overhangs were added and oligos were phosphorylated, annealed and ligated into the px459 v2.0 (HeLa) or px458-GFP (HEK293) predigested with BbsI. The *hHIF1AN* clone number #1 was generated with the combination of hHIF1AN guide #2 and hHIF1AN guide #3, while the hHIF1AN clone number #2 was generated with the combination of hHIF1AN guide #1 and hHIF1AN guide #3. The HEK293-Notch ΔPEST clone was generated with hN1 guide #1. The HEK293-Notch depleted clones #2 and #24 were generated with the combination of hN1 guide #2 and hN1 guide #3 (sequences of the guides in Table S10). HeLa cells were transfected with 10 μg of each px459 v2.0 plasmid and HEK293 cells were transfected with 10 μg of each px458-GFP plasmid together with 40 μl of linear PEI (Polyscience 23966) using standard protocols. Cells were either selected with puromycin (HeLa) or sorted for GFP expression (HEK293) before establishing single cell clones. Genomic DNA (gDNA) was purified as follows: Briefly, after washing twice in PBS, cells were resuspended in gDNA extraction buffer [10 mM Tris–HCl pH 7.5, 10 mM EDTA pH 8.0, 10 mM NaCl, 0.5% *N*-Lauroylsarcosine sodium salt, 1 mg*/*mL proteinase K (Roche 03115852001)] and incubated over night at 37°C. gDNA was precipitated with 100% EtOH in presence of 50 mM NaCl, washed with 70% EtOH, dried and resuspended in TE pH 8.0. gDNA was used for PCR screening with the PCR primers listed in Table S10 and the PCR products were analyzed by agarose gel electrophoresis.

**Constructs**

All oligonucleotides used for cloning purposes are listed in Table S10. PCR products were cloned in the pSC-A-amp/kan (Agilent Technologies 240205-5), digested with the desired restriction enzymes (New England Biolabs) and cloned into the destination vectors accordingly to Table S11. All plasmids were analyzed by sequencing.

The mouse Notch1-specific construct for mRNA synthesis pCS2-N1ΔE (wt) [1], the luciferase based reporter construct pGa981/6 [2] and the reporter plasmid 12 × CSLRE-EGFP [1] were described previously. For construction of the pCS2-N1ΔE (NNAA) plasmid, an *AfeI/EcoRV* fragment from pcDNA3-FLAG-mNICD1 (NNAA) was inserted into the corresponding sites of pCS2-N1ΔE (wt). The pcDNA 3.1 FLAG2 (Invitrogen) was commercially acquired. The pcDNA3-FLAG-mNICD1 wt, pcDNA3.1 FLAG-mNICD1 ΔOP, pcDNA3-FLAG-mNICD1ExB, pcDNA3-FLAG-mNICD1 EhxB, pcDNA3.1 HA, pMT123-HA-8 x –Ubiquitin, pLX304 USP10 V5, pMy BioNICD ΔOP, pMy NCMXH BioFLAG-NICD1 wt pSV40 PURO and pMy Bio NCMXH oligo pSV40 PURO plasmids were previously described [3, 4, 5, 6].

The pMy NCMXH BioFLAG-NICD1 NNAA pSV40 PURO was generated by Acc65I*/*XbaI digestion of the pcDNA3-FLAG-mNICD1 NNAA. The digestion product was blunt ended with T4 DNA polymerase (New England Biolabs M0203S) and ligated into the pMy Bio NCMXH oligo pSV40 PURO predigested with MfeI*/*HindIII.

The expression vector pCMV-6HIS HA-Ub K11 only, pCMV-6HIS HA-Ub K11R, pCMV-6HIS HA-Ub K48 only, pCMV-6HIS HA-Ub K48R, pCMV-6HIS HA-Ub K48 only, pCMV-6HIS HA-Ub K48R were a generous gift of Prof. Dr. L. Schmitz (University of Giessen, Germany). The pcDNA FLAG-hFIH and the pcDNA3-FLAG-mNICD1 NNAA were a generous gift of Roxana Solga. The pcDNA3.1 HA-mNICD1 and the pcDNA3.1-HA-mNICD1 NNAA were a generous gift of Dr. Kerstin Hein. The px459 v2.0 was acquired from Addgene (#62988) [7]. The px458 vector [pSpCas9(BB)-2A-GFP] was a gift from Feng Zhang (MIT, Cambridge, MA) (Addgene plasmid #48138) [7]

The pcDNA HA-hFIH catalytic dead (CD) mutant (D201A/H199A double mutant) was generated by site directed mutagenesis using the QuickChange II XL Site-Directed Mutagenesis Kit (Agilent Technologies 200521–5) with primers listed in Table S10.

**Protein extracts and Western blotting**

Whole cell extracts (WCE) were prepared as follows: Briefly, cells were washed twice with ice-cold PBS and resuspended in WCE buffer (20 mM Tris-HCl pH 8.0, 150 mM NaCl, 1% NP-40, 10% glycerol, 0.5 mM Na_3_VO_4_, 10 mM NaF, 1 mM PMSF, 1x protease inhibitor cocktail mix). After 15 min incubation on ice, lysates were centrifuged (13.200 rpm, 15 min, 4°C). Protein concentrations were determined by Bradford assay (Sigma-Aldrich) and extracts were analyzed by Western blotting.

For Western blotting, proteins were resolved in SDS polyacrylamide gels and transferred to a Hybond-P PVDF membrane (Amersham) by wet blotting. Membranes were blocked in 5% milk / TBST (1x TBS, 0.1% Tween 20) before adding the desired antibody diluted in 5% milk / TBST [1:2000 GAPDH (abcam ab8245); 1:5000 FLAG (Sigma-Aldrich F3165); 1:5000 HA (3F10, Roche 1867423); 1:100 NICD1 N1945-OH, 1:1000 H3 (abcam ab1791); 1:1000 Val1744 cleaved NICD1 (Cell Signaling 2421); 1:1000 USP10 (Cell Signaling 5553); 1:1000 V5 (Invitrogen R960-50); 1:1000 HIF1α (Cell Signaling Technology 36169S); 1:1000 HIF2α (Abcam ab199); 1:1000 TBP (Santa Cruz sc-273); 1:200 RNAPII (Santa Cruz sc-899); 1:1000 GFP (Roche 11814460001); 1:1000 FIH (Cell Signaling 4426); 1:2000 α-Tubulin (Sigma-Aldrich T9026); 1:1000 RBPJ (CosmoBio T6709); 1:1000 MAML1 (abcam ab155786); 1:1000 FBXW7 (abcam ab227677)]. Membranes were washed in TBST and incubated 1 hour at room temperature with secondary antibody diluted 1:5000 in 5% milk / TBST [anti-rat IgG HRP (Jackson ImmunoResearch 112-035-072), anti-mouse IgG HRP (Cell Signaling 7076S) or anti-rabbit IgG HRP (Cell Signaling 7074S)]. Membranes were finally washed in TBST.

All membranes were incubated at room temperature with ECL solution and chemiluminescence was detected with a light sensitive film or with a Vilber Fusion FX7 system.

**Co-immunoprecipitation of Notch co-activator components**

HEK293 cells were transfected with expression constructs for NICD variants, RBPJ and MAML1 as indicated. 24 h after transfection, cells were lysed in 600 μl CHAPS lysis buffer [10 mM 3-[(3-Cholamidopropyl)-dimethylammonio]-1-propanesulfonate hydrate (CHAPS, Merck, C3023), 50 mM Tris-HCl (pH7.8), 150 mM NaCl, 5 mM NaF, 0.5 mM Phenylmethanesulfonyl fluoride (PMSF) (Merck, P-7626) and 40 μl/ml cOmplete protease inhibitor cocktail (Roche, 13539320)]. Extracts were incubated with FLAG-M2 affinity gel (Sigma-Aldrich, Merck A2220) at 4°C overnight. After 6 to 8 times washing with CHAPS lysis buffer precipitates were resuspended in 1x SDS-polyacrylamide gel loading buffer. Samples were analyzes by Western blotting as described above.

**Ubiquitination assay**

To detect ubiquitination of NICD1, Phoenix^TM^ cells were transfected with pcDNA3-FLAG-mNICD1 wt or pcDNA3-FLAG-mNICD1 NNAA plasmids with or without pMT123-HA-8x–Ub, pCMV-6HIS HA-Ub K11 only, pCMV-6HIS HA-Ub K11R, pCMV-6HIS HA-Ub K48 only, pCMV-6HIS HA-Ub K48R, pCMV-6HIS HA-Ub K63 only or pCMV-6HIS HA-Ub K63R using the calcium phosphate method as described above. 36 hours post-transfection, 20 μM MG132 were added and after 6 hours, cells were washed twice with PBS. Cells were lysed in 300 μL of denaturing lysis buffer (20 mM Tris-HCl pH 7.4, 5 mM EDTA, 2% SDS, 10 mM DTT) and, after incubation for 15 min at 99°C, samples were diluted ten times in IP-buffer (50 mM Tris-HCl pH 7.4, 300 mM NaCl, 5 mM EDTA, 1% NP-40, 1 mM PMSF, 1x protease inhibitor cocktail mix). FLAG-tagged proteins were immunoprecipitated with FLAG-M2 affinity gel (Sigma-Aldrich, Merck A2220) and beads were washed with IP-buffer and PBS. Immunoprecipitates were analyzed by Western blotting using HA (3F10, Roche 1867423) and FLAG (Sigma-Aldrich F3165) antibodies. Alternatively, Phoenix^TM^ cells were transfected with pcDNA3-FLAG-mNICD1 wt or pcDNA3-FLAG-mNICD1 NNAA plasmids, using the calcium phosphate method. 36 hours post-transfection, 20 μM MG132 were added and after 6 hours, cells were washed twice with PBS. Extracts were prepared using WCE buffer and boiled 5 min at 95°C in SDS loading buffer. Samples were analyzed by Western blotting as described above.

**TUBE Assay**

To detect endogenously ubiquitinated NICD1, HEK293T cells were transfected with the pcDNA3.1 HA-mNICD1, the pcDNA3.1-HA-mNICD1 NNAA or the pcDNA3.1 HA as a control. 36 hours post-transfection, 20 μM MG132 were added and after 6 hours, cells were washed twice with PBS. After resuspending the cells in Cell Lysis Buffer (50 mM Tris-HCl pH 7.5, 1% Triton-X-100, 1 mM EGTA, 1 mM EDTA, 0.27 M sucrose, 1 mM benzamidine, 1 mM PMSF, 10 mM sodium 2-glycerophosphate, 20mM NEM, 50 mM NaF, 5 mM Na_4_P_2_O_7_, 1 mM sodium orthovanadate, Protease Inhibitor Cocktail), the cell extracts were incubated at 4°C for 1 hour. 3 mg of cell extract were incubated with 30 μL TUBE Halo affinity beads at 4°C for 4 hours. Extracts were washed using IP-Wash buffer (500 mM NaCl, 50 mM Tris-HCl pH 7.5, 1% Triton-X-100, 1 mM EGTA, 1 mM EDTA, 0.27 M sucrose, 1 mM benzamidine, 1 mM PMSF, 10 mM sodium 2-glycerophosphate, 20 mM NEM, 50 mM NaF, 5 mM Na4P2O7, 1 mM sodium orthovanadate, Protease Inhibitor Cocktail), and boiled 5 min at 95°C in SDS loading buffer. Ubiquitinated NICD1 was detected by Western blotting as described above using an HA antibody or a GAPDH antibody as a loading control.

To detect endogenous ubiquitinated NICD1 in Beko cells, cells were treated for 6 hours with 0.5 mM DMOG or DMSO as a control in combination with 5 μM MG132. The TUBE assay was performed as described above and analyzed by Western blotting as described above using an NICD1 antibody or a GAPDH antibody as a loading control.

**Cycloheximide Chase (CHX) Assay**

To perform the cycloheximide (CHX) assay in Phoenix^TM^ cells overexpressing the HA-NICD1 wt or HA-NICD1 NNAA mutant, one million of cells per time point were seeded in a 6 cm^2^ culture dish, treated with 150 µg/mL CHX, collected at the indicated time points and WCE were prepared as described above. Proteins were analyzed by Western blotting as described above and quantification was done using ImageJ 1.48v. NICD1 protein abundance was normalized to the loading control GAPDH.

**Luciferase assay**

HeLa cells (2.25 × 10^4^ cells/well) were seeded in 48-well plates. Cell transfection was performed using the Lipofectamine 2000 transfection reagent (Invitrogen, #11668019). Reporter plasmid (0.25 µg/well) was transfected alone or together with 10 ng of NICD1 expression plasmid (wild-type or NNAA mutant). After 24 hours, luciferase activity was determined from at least four independent experiments with 10 μl of cleared lysate in an Centro LB 960 Microplate luminometer (Berthold) by using the Dual-Glo® Luciferase Assay System from Promega (#E2980).

# Fluorescence Microscopy

# HeLa cells were seeded (1 x 10^5^ cells/cm^2^) on chamber coverslips (Nunc-Lab-Tek, #155380). After 18 hours, cells were transfected with 150 ng of NICD1 specific expression plasmids. 24 hours after transfection, cells were rinsed with PBS, fixed [0.4% PFA (Merck)] and permeabilized with 0.1 % Triton X-100. Nonspecific immunostaining was blocked by incubating the cells in 0.2 % fish-skin gelatine (Sigma-Aldrich) in PBS with 0.1 % TWEEN-20. To detect the FLAG-tagged NICD variants the following antibodies were used: anti-FLAG, mouse monoclonal IgG, (Sigma-Aldrich F4042); 1:10000, secondary antibody, Alexa-Fluor-568 coupled goat anti-mouse IgG (Invitrogen A11004); 1:1000. For detection of NICD1-GFP variants no incubation with antibodies was needed. Specimens were embedded in ProLong© Gold antifade reagent (Invitrogen) supplemented with 2-(4-carbamimidoylphenyl)-1H-indol-6-carbox-imidamide (DAPI) and stored at 4°C overnight. Images were taken using a fluorescence microscope (IX71, Olympus) equipped with a digital camera (C4742, Hamamatsu) and a 100-W mercury lamp (HBO 103W/2, Osram). The following filtersets were used: GFP detection: excitation, HQ470/40; emission, HQ525/50. Alexa Fluor 568 detection: excitation, HQ545/30; emission, HQ610/75. DAPI detection: D360/50; emission: D460/50. The images were acquired with Simple PCI software (Hamamatsu) and edited with the "ImageJ" software (<https://imagej.nih.gov/ij/>).

**RNA extraction, reverse transcription, quantitative PCR (qPCR), libraries and sequencing**

Total RNA was purified using Trizol reagent (Ambion, 15596018) accordingly to manufacturer’s instructions. 1 μg of RNA was retro-transcribed in cDNA using random hexamers and M-MuLV reverse transcriptase (NEB). qPCRs were assembled with Absolute QPCR ROX Mix (Thermo Scientific AB-1139), gene-specific oligonucleotides and double-dye probes (Table S10) and analyzed using the StepOnePlus^TM^ sequence detector system (Applied Biosystem). Data were normalized to the housekeeping gene *Hypoxanthine Phosphoribosyltransferase 1* (*HPRT*).

# For RNA-Seq purposes, total RNA was purified using the RNeasy Mini Kit (Qiagen #74104), the QIAshredder (Qiagen #79654) and the DNase I (Qiagen #79254) accordingly to manufacturer´s instructions. Libraries were prepared and sequenced at Centro de Análisis Genómico (CNAG-CRG) Spain or at Novogene.

**Chromatin immunoprecipitation (ChIP), libraries and sequencing**

ChIP-Seq experiments were performed essentially as previously described [3, 8] using chromatin from *Drosophila melanogaster* Schneider cells for spike-in purposes (each 25 μg of mouse chromatin, 25 ng of *Drosophila* chromatin were used). 2 μg of anti-His2Av (Active Motif 61686) were added to each immunoprecipitation for spike-in purposes in combination with the RBPJ antibody (Cell Signaling 5313) or the HIF1α antibody (Abcam 228649; Diagenode C15410234). Libraries were prepared using the Diagenode MicroPlex Library Preparation kit v2 (Diagenode) following manufacturer’s instructions with few modifications. Libraries were purified with Agencourt AMPure XP Beads (Beckman Coulter, #A63881), quantified, analyzed on a Tapestaion device (Agilent) and pooled. Finally, sequencing was performed by Centro de Análisis Genómico (CNAG-CRG), Spain.

The STREPTAVIDIN ChIP was essentially performed as previously described [1].

ChIP experiments were analyzed on a StepOnePlus^TM^ sequence detector system (Applied Biosystem) using specific oligonucleotides and double-dye probes (Table S10).

**Bioinformatics analysis**

ChIP-Seq and RNA-Seq data were jointly analyzed with the previously published RBPJ ChIP-Seq (GEO: GSE119797) and RNA-Seq data (GEO: GSE106361) [3]. All primary genomics analyses were performed within R v. 4.0.2 [9] using a custom adapted version of the systemPipeR R/BioConductor package [10, 11]. Raw sequencing reads were adapter and quality trimmed using trimGalore v. 0.6.5 [12] with standard parameters. The effects of the trimming were evaluated by visual inspection of the FASTQ files using systemPipeR’s *seeFastq* function. Both trimmed ChIP-Seq and RNA-Seq reads were aligned against the mouse genome (*mm9*) using bowtie2 v. 2.3.5.1 [13], tophat v 2.1.1 using the parameters “-g 1 --segment-length 25 -i 30 -I 3000” [14] or hisat2 v. 2.2.1 [15] with parameter “—min-intronlen 30 –max-intronlen 3000”. The resulting sequence alignment maps were directly converted into binary alignment maps (BAM) using the systemPipeR’s *runCommandline* function with the parameter “make_bam=TRUE“. The quality of those BAM files was validated using the systemPipeR’s *alignStats* function. PCR duplicates for both ChIP-Seq and RNA-Seq BAM files were removed using picard tools v. 2.21.9 available at http://broadinstitute.github.io/picard/.

Peak calling for the 5 RBPJ DMSO ChIP-Seq BAM files was performed using peakranger v. 1.18 [16]. MSPC v. 4.0.0 [17] with parameters “-r bio -w 1e-6 -s 1e-10” was used to evaluate the called peaks and determine the “true positive peaks”. Only “true positive peaks” that were conserved in 3 out of 5 replicates were used as the final set of binding sites. Association of those binding sites to their potential target genes was performed using the *annotateRanges* function of the PETRA package. The *annotate Ranges* function uses a comparable annotation strategy as GREAT’s (<http://great.stanford.edu/public/html/>) basal plus extension model.

Peak calling for the HIF1α ChIP-Seq BAM files was performed using MACS2 v. 2.2.7. (PMID:18798982). Only peaks that are conserved in 2 out of 4 samples were used as the final set of HIF1α binding sites. Motif discovery was performed using MEME suite v. 5.0.5 (PMID:19458158).

RNA-Seq data were analyzed as follows: A gene count table, based on the RNA-Seq BAM files, was created using the *summarizeOverlaps* function [18] along with a mouse *mm9*-specific GTF file (Illumina’s IGenomes). Normalization and identification of differentially expressed genes based on the gene expression matrix was done using DESeq2 v. 1.28.1 [19].

Genes with a log2[Fold change(FC)] > 1 or < -1 and an adjusted p-value < 0.05 were identified as significantly deregulated. *Bona fide* Notch target genes were defined as those genes that are significant downregulated upon GSI treatment and associated with an RBPJ binding site. We used the clusterProfiler package [20] for the over-representation and gene set enrichment analyses of KEGG and GO categories. We defined the universe as all genes that are at least expressed in at least one sample. DESeq2’s Wald-test statistic was used as the input for GSEA. Plots were generated using the *ggplot2 [https://cran.r-project.org/web/packages/ggplot2/citation.html]*, *boxplot* and *heatmap.2* functions.

**Zebrafish strains, RNA microinjections and injection procedures**

Care and breeding of zebrafish (*D. rerio*) were performed as described previously (Just et al., 2011). The study was performed after securing appropriate institutional approvals (Tierforschungszentrum Ulm University, No. 0183) and conformed with the EU Directive 2010/63/EU. The TüAB wild-type strain was used for all injection procedures. Sense-capped mRNA was synthesized as follows: After linearization of the respective pCS2 vector with *Kpn*I (NEB) mouse N1ΔE mRNAs (wild-type and NNAA mutant) were obtained using the mMESSAGE mMACHINE SP6 Kit (Invitrogen AM1340) following the manufacturer’s protocol. The mRNA was diluted (20 ng/µl in 0.2 M KCl) and co-microinjected with reporter plasmid (12 × CSLRE-EGFP; 10 ng/µl in 0.2 M KCl) into one-cell-stage embryos. Siblings from the same pool were injected using only the reporter DNA as a control. Pictures were recorded at 24 hpf with an Olympus SZX16 stereomicroscope.

**SUPPLEMENTAL REFERENCES**

1. Hein K, Mittler G, Cizelsky W, Kuhl M, Ferrante F, Liefke R*, et al.* Site-specific methylation of Notch1 controls the amplitude and duration of the Notch1 response. *Sci Signal* 2015, **8**(369)**:** ra30.

2. Oswald F, Liptay S, Adler G, Schmid RM. NF-kappaB2 is a putative target gene of activated Notch-1 via RBP-Jkappa. *Mol Cell Biol* 1998, **18**(4)**:** 2077-2088.

3. Ferrante F, Giaimo BD, Bartkuhn M, Zimmermann T, Close V, Mertens D*, et al.* HDAC3 functions as a positive regulator in Notch signal transduction. *Nucleic Acids Res* 2020, **48**(7)**:** 3496-3512.

4. Oswald F, Tauber B, Dobner T, Bourteele S, Kostezka U, Adler G*, et al.* p300 acts as a transcriptional coactivator for mammalian Notch-1. *Mol Cell Biol* 2001, **21**(22)**:** 7761-7774.

5. Lim R, Sugino T, Nolte H, Andrade J, Zimmermann B, Shi C*, et al.* Deubiquitinase USP10 regulates Notch signaling in the endothelium. *Science* 2019, **364**(6436)**:** 188-193.

6. Giaimo BD, Ferrante F, Vallejo DM, Hein K, Gutierrez-Perez I, Nist A*, et al.* Histone variant H2A.Z deposition and acetylation directs the canonical Notch signaling response. *Nucleic Acids Res* 2018, **46**(16)**:** 8197-8215.

7. Ran FA, Hsu PD, Wright J, Agarwala V, Scott DA, Zhang F. Genome engineering using the CRISPR-Cas9 system. *Nat Protoc* 2013, **8**(11)**:** 2281-2308.

8. Dieguez-Hurtado R, Kato K, Giaimo BD, Nieminen-Kelha M, Arf H, Ferrante F*, et al.* Loss of the transcription factor RBPJ induces disease-promoting properties in brain pericytes. *Nat Commun* 2019, **10**(1)**:** 2817.

9. R-Core-Team. R: A language and environment for statistical computing. R Foundation for Statistical Computing. *Vienna, Austria* 2018.

10. Reimers M, Carey VJ. Bioconductor: an open source framework for bioinformatics and computational biology. *Methods Enzymol* 2006, **411:** 119-134.

11. TW HB, Girke T. systemPipeR: NGS workflow and report generation environment. *BMC Bioinformatics* 2016, **17:** 388.

12. Krueger F. Trim galore. A wrapper tool around Cutadapt and FastQC to consistently apply quality and adapter trimming to FastQ files. 2015.

13. Langmead B, Salzberg SL. Fast gapped-read alignment with Bowtie 2. *Nat Methods* 2012, **9**(4)**:** 357-359.

14. Trapnell C, Pachter L, Salzberg SL. TopHat: discovering splice junctions with RNA-Seq. *Bioinformatics* 2009, **25**(9)**:** 1105-1111.

15. Kim D, Langmead B, Salzberg SL. HISAT: a fast spliced aligner with low memory requirements. *Nat Methods* 2015, **12**(4)**:** 357-360.

16. Feng X, Grossman R, Stein L. PeakRanger: a cloud-enabled peak caller for ChIP-seq data. *BMC Bioinformatics* 2011, **12:** 139.

17. Jalili V, Matteucci M, Masseroli M, Morelli MJ. Using combined evidence from replicates to evaluate ChIP-seq peaks. *Bioinformatics* 2015, **31**(17)**:** 2761-2769.

18. Lawrence M, Huber W, Pages H, Aboyoun P, Carlson M, Gentleman R*, et al.* Software for computing and annotating genomic ranges. *PLoS Comput Biol* 2013, **9**(8)**:** e1003118.

19. Love MI, Huber W, Anders S. Moderated estimation of fold change and dispersion for RNA-seq data with DESeq2. *Genome Biol* 2014, **15**(12)**:** 550.

20. Yu G, Wang LG, Han Y, He QY. clusterProfiler: an R package for comparing biological themes among gene clusters. *OMICS* 2012, **16**(5)**:** 284-287.

**SUPPLEMENTAL FIGURE LEGENDS**

**Fig. S1 Hypoxia and DMOG treatment have a similar gene expression profile.** Beko cells were treated with 0.5 mM DMOG or DMSO as a control for 24 hours. Alternatively, cells were kept for 12 hours in hypoxia (1% O_2_) or normoxia (5% O_2_) as a control. **A and B** Whole cell extracts (WCE) were analyzed by Western blotting (WB) versus the endogenous HIF1α, HIF2α or GAPDH as a loading control. **C-H** RNA was purified and analyzed by deep sequencing or qPCR. **C** Heat map showing the sample wise correlation of the different RNA-Seq samples in Beko cells. Shown is the Pearson correlation coefficient (PCC) based on the union of all significantly deregulates genes (log2FC > 1 or < -1 and adjusted p-value < 0.05) upon DMOG/DMSO and hypoxia/normoxia. **D** Heat map showing the gene expression profile of DMOG treatment or hypoxia in Beko cells. Shown are log2FC of the union of all significantly deregulated genes by DMOG/DMSO or hypoxia/normoxia. **E** Venn diagram of significantly deregulated genes upon hypoxia or DMOG treatment in Beko cells show a significant (Fisher’s exact test: P < 0.001) set of 780 genes that are commonly deregulated between both conditions. **F and G** Box plots showing the effects of DMOG treatment or hypoxia in Beko cells for **F** genes upregulated or downregulated upon hypoxia or, alternatively, for **G** genes upregulated or downregulated upon DMOG treatment. Wilcoxon rank sum tests (****P <* 0.001). **H** Gene over-representation analysis based on the KEGG database for genes commonly upregulated by hypoxia and DMOG treatment in Beko cells. **I and J** Validation of the RNA-Seq experiments by qPCR for hypoxia target genes. *Vegfa*, *Smad3* and *Tgfb3* are upregulated upon **I** hypoxia or **J** DMOG treatment in Beko cells. Upon RNA extraction and reverse transcription, cDNAs were analyzed by qPCR using primers specific for *Tbp*, *Vegfa*, *Smad3* or *Tgfb3*. Data were normalized to the housekeeping gene *Hypoxanthine* *Guanine* *Phosphoribosyltransferase* (*Hprt*). Shown is the mean ± SD of five independent experiments (N=5; [*] P < 0.05, [**] P < 0.01, [***] P < 0.001 unpaired Student’s *t*-test).

**Fig. S2 Hypoxia downregulates the Notch-dependent gene expression program.** Beko cells were treated with 0.5 mM DMOG or with DMSO as a control for 24 hours. Alternatively, cells were kept for 12 hours in hypoxia (1% O_2_) or normoxia (5% O_2_) as a control. RNA was purified and analyzed by deep sequencing or qPCR. **A and B** GSEA plot of the term “Notch signaling pathway” using DESeq2’s Wald-test statistic for **A** DMOG/DMSO or **B** hypoxia/normoxia. **C and D** Validation of the RNA-Seq experiment by qPCR. *Ngfr* and *Necab1* are downregulated upon **C** hypoxia or **D** DMOG treatment in Beko cells. Upon RNA extraction and reverse transcription, cDNAs were analyzed by qPCR using primers specific for *Ngfr* and *Necab1*. Data were normalized to the housekeeping gene *Hypoxanthine* *Guanine* *Phosphoribosyltransferase* (*Hprt*). Shown is the mean ± SD of five independent experiments (N=5; [*] P < 0.05, unpaired Student’s *t*-test).

**Fig. S3 Characterization of the genomic binding of HIF1α and RBPJ upon hypoxia in Beko cells.** Genome-wide occupancy of HIF1α and RBPJ was investigated by ChIP-Seq in Beko cells kept under normoxia or under hypoxia for 12 hours. **A** Heat map shows increased HIF1α binding in Beko cells upon hypoxia as detected using two different antibodies but **B** no changes are observed on the genomic occupancy of RBPJ. **C** De novo motif analysis of the HIF1α ChIP-Seq identifies the hypoxia responsive element (HRE). **D** Heat map showing the list of genes deregulated upon DMOG treatment or hypoxia in Beko cells and bound by HIF1α. Shown are log2FC of the union of all significantly deregulated genes by DMOG/DMSO and hypoxia/normoxia. **E-F** Snapshot representing the binding of HIF1α and RBPJ at the **E** *Hes1* and **F** *Hey1*.

**Fig. S4 Interaction of FIH and NICD1.** **A** Beko cells were treated with 1 μM or 10 μM Roxadustat or with DMSO as a control for 24 hours. Whole cell extracts (WCE) were analyzed by Western blotting versus the endogenous cleaved NICD1 protein or TBP as a loading control. **B and C** NICD1 and FIH interact to each other in Beko cells. Beko cells were infected with plasmids encoding GFP-tagged NICD1 ΔOP mutant or empty vector as a control (GFP). **B** WCE were analyzed for expression of the proteins of interest by Western blotting versus GFP or RNA polymerase II (RNAPII) as a loading control. (C) WCE were subjected to GFP immunoprecipitation (GFP-IP) and the immunoprecipitates were analyzed by Western blotting versus GFP or endogenous FIH. **D and E** The NICD1/FIH interaction depends on the ankyrin (ANK) repeats of the NICD1. Phoenix^TM^ cells were transfected with plasmids encoding for FLAG-tagged NICD1 wild-type (wt), FLAG-tagged NICD1 deleted of the OPA and PEST domains (ΔOP), FLAG-tagged NICD1 ExB mutant, FLAG-tagged NICD1 EhxB mutant and/or HA-tagged FIH. WCE were subjected to FLAG immunoprecipitation (FLAG-IP) and the immunoprecipitates were analyzed by Western blotting versus FLAG or HA. **F and G** NICD1 and FIH co-localize at the enhancers of Notch target genes in Beko cells. STREPTAVIDIN ChIP experiments showing the occupancy of F NICD1 and **G** FIH at the enhancers of *Ptcra* and *Uaca* Notch target genes in Beko cells, expressing the biotin ligase BirA and infected with plasmids encoding BioNICD1 ΔOP, BioFIH or empty vector as a control (Bio). *Chrom X* was used as a negative control. The distance relative to the transcription starting site (TSS) is indicated for each enhancer. Shown is the mean ± SD of three independent experiments (N=3; [*] P < 0.05, [NS] not significant, unpaired Student’s *t*-test). **H** DMOG treatment destabilizes the NICD1 protein. Beko cells were treated for 24 hours with 0.5 mM DMOG or DMSO as a control and, after the first 16 hours, protein synthesis was blocked by adding 50 µg/mL cycloheximide (CHX). Samples were collected at the indicated time points. WCE was analyzed by WB versus endogenous cleaved NICD1 or GAPDH as a loading control. Quantification of the NICD1 levels normalized to GAPDH is shown on the right. The experiment was repeated independently three times.

**Fig. S5 Validation of the NICD1 N1945-OH antibody.** **A** Validation of HEK293 cells depleted for the *NOTCH1* gene or of only the sequence encoding for its C-terminal PEST domain. Whole cell extracts (WCE) were analyzed by Western blotting versus NICD1 or tubulin as a loading control. “Ctr” indicates wild-type HEK293 cells. **B** The NICD1 N1945-OH antibody specifically recognizes the NICD1 in HEK293 cells. HEK293 cells depleted of the *NOTCH1* gene (clone *NOTCH1* KO #2) were transfected with plasmids encoding for FLAG-tagged NICD1 wild-type (wt), FLAG-tagged NICD1 deleted of the OPA and PEST domains (ΔOP), FLAG-tagged NICD1 ΔOP NNAA mutant, FLAG-tagged NICD1 ΔOP N1945A mutant, FLAG-tagged NICD1 ΔOP N2012A mutant or empty vector as a control (eV). WCE were subjected to FLAG immunoprecipitation (FLAG-IP) and the immunoprecipitates were analyzed by Western blotting versus NICD1 N1945-OH or FLAG antibody. “Ctr” indicates wild-type HEK293 cells transfected with FLAG-tagged NICD. **C and D** The NICD1 N1945-OH antibody specifically recognizes the NICD1 in Beko cells. Beko cells were kept for **C** 12 hours in hypoxia (1% O_2_) or normoxia (5% O_2_) as a control or, alternatively, **D** treated with 0.5 mM DMOG or with DMSO as a control for 24 hours. WCE were analyzed by Western blotting versus the endogenous NICD1 N1945-OH or TBP as a loading control. **E** Roxadustat does not influence the levels of hydroxylated NICD1. Beko cells were treated with 1 μM or 10 μM Roxadustat or with DMSO as a control for 24 hours. WCE were analyzed by Western blotting versus the endogenous NICD1 N1945-OH or TBP as a loading control.

**Fig. S6 Hypoxia downregulates the Notch signalling pathway in RPMI-8402 cells.** **A and B** RPMI-8402 cells were treated with 0.5 mM DMOG or with DMSO as a control for 24 hours. Alternatively, cells were kept for 12 hours in hypoxia (1% O_2_) or normoxia (5% O_2_) as a control. RNA was purified and analyzed by qPCR using primers versus *TBP*, *HES4* or *HEYL*. *JMJD1A* was used as a positive control for the induction of the hypoxia response. Data were normalized to the housekeeping gene *Hypoxanthine* *Guanine* *Phosphoribosyltransferase* (*Hprt*). Shown is the mean ± SD of **A** three independent experiments measured twice each and **B** four independent experiments measured twice each (**A**: N=6; **B**: N=8; [*] P < 0.05, [***] P < 0.001, [NS] not significant unpaired Student’s *t*-test). **C and D** RPMI-8402 cells were kept for **C** 12 hours in hypoxia (1% O_2_) or normoxia (5% O_2_) as a control or, alternatively, **D** treated with 0.5 mM DMOG or with DMSO as a control for 24 hours. Whole cell extracts (WCE) were analyzed by Western blotting (WB) versus the endogenous cleaved NICD1 protein or TBP as a loading control. **E and F** The NICD1 N1945-OH antibody specifically recognizes the NICD1 in RPMI-8402 cells. RPMI-8402 cells were kept for **E** 12 hours in hypoxia (1% O_2_) or normoxia (5% O_2_) as a control or, alternatively, **F** treated with 0.5 mM DMOG or with DMSO as a control for 24 hours. WCE were analyzed by Western blotting versus the endogenous NICD1 N1945-OH or TBP as a loading control.

**Fig. S7 FIH hydroxylates NICD1. A-D** Generation of FIH depleted HeLa cells. **A** Scheme of the targeting strategy via the CRISPR/Cas9 system in HeLa cells. **B** Agarose gel analysis of two independent FIH depleted HeLa cell clones. The expected band at 370 bp is not visible in both FIH depleted clones. “Ctr” indicates HeLa cells transfected with the CRISPR/Cas9 plasmid lacking of sgRNA. **C** FIH protein is not detectable in FIH depleted HeLa cell clones. WCE from control (Ctr) or FIH depleted HeLa cell clones were analyzed by Western blotting versus FIH or H3 as a loading control. **D** FIH depletion leads to abolishment of NICD1 hydroxylation. WCE from control (Ctr) or FIH depleted HeLa cell clones were analyzed by Western blotting versus the endogenous NICD1 N1945-OH or TBP as a loading control. **E** FIH hydroxylates NICD1 at N1945. Phoenix^TM^ cells were transfected with plasmids encoding for FLAG-tagged NICD1 wild-type (wt), FLAG-tagged NICD1 NNAA mutant, FLAG-tagged NICD1 N1945A mutant, FLAG-tagged NICD1 N2012A mutant and/or HA-tagged FIH. After 6 hours of treatment with 20 μM of MG132 to block the activity of the proteasome, WCE were prepared and subjected to FLAG immunoprecipitation (FLAG-IP). The immunoprecipitates were analyzed by Western blotting versus FLAG, HA or NICD1 N1945-OH and reblotted (RB) versus the FLAG-tagged NICD1 proteins.

**Fig. S8 The hydroxylation-defective NICD1 mutants interact with RBPJ and MAML1.** **A and B** NICD1 wt and NICD1 NNAA mutant similarly interact with endogenous RBPJ. **A** HEK293 cells were transfected with plasmids encoding for FLAG-tagged NICD1 deleted of the OPA and PEST domains (ΔOP) wild-type (wt), FLAG-tagged NICD1 ΔOP NNAA mutant, FLAG-tagged NICD1 ΔOP N1945A mutant or FLAG-tagged NICD1 ΔOP N2012A mutant. “Ctr” indicates lysates from HEK293 cells as positive controls. WCE were subjected to FLAG immunoprecipitation (FLAG-IP) and the immunoprecipitates were analyzed by Western blotting using antibodies against RBPJ or FLAG. **B** HEK293 cells were transfected with plasmids encoding for FLAG-tagged NICD1 wild-type (wt), FLAG-tagged NICD1 NNAA mutant, FLAG-tagged NICD1 deleted of the OPA and PEST domains (ΔOP) wild-type (wt) or FLAG-tagged NICD1 ΔOP NNAA mutant. “Ctr” indicates lysates from HEK293 cells as positive controls. WCE were subjected to FLAG immunoprecipitation (FLAG-IP) and the immunoprecipitates were analyzed by Western blotting versus RBPJ or FLAG. **C and D** NICD1 wt and NICD1 NNAA mutant similarly form a coactivator complex with RBPJ and MAML1. **C** HEK293 cells were transfected with plasmids encoding for FLAG-tagged NICD1 wild-type (wt), FLAG-tagged NICD1 NNAA mutant, FLAG-tagged NICD1 N1945A mutant or FLAG-tagged NICD1 N2012A mutant together with FLAG-tagged RBPJ and/or MAML1. WCE were subjected to FLAG immunoprecipitation (FLAG-IP) and the immunoprecipitates were analyzed by Western blotting with MAML1 or FLAG antibody. **D** HEK293 cells were transfected with plasmids encoding for FLAG-tagged NICD1 deleted of the OPA and PEST domains (ΔOP) wild-type (wt), FLAG-tagged NICD1 ΔOP NNAA mutant, FLAG-tagged NICD1 ΔOP N1945A mutant or FLAG-tagged NICD1 ΔOP N2012A mutant together with FLAG-tagged RBPJ and/or MAML1. WCE were subjected to FLAG immunoprecipitation (FLAG-IP) and the immunoprecipitates were analyzed by Western blotting versus MAML1 or FLAG. **E** NICD1 wt and NICD1 NNAA mutant predominantly localize to the nucleus of HeLa cells. HeLa cells were transfected with plasmids encoding for NICD1-DOP wild-type (wt), NICD1-DOP (NNAA), NICD1 (wt) NICD1 (NNAA) either FLAG-tagged (left) or GFP-tagged (right). Flag-tagged NICD protein were detected by using an anti-Flag antibody (left) GFP-tagged NICD1 proteins were detected by direct fluorescence microscopy. Nuclei were labelled by DAPI staining. Scale bar: 20 µm.

**Fig. S9 Hypoxia increases both degradative and regulatory NICD1 ubiquitination.** **A** Phoenix^TM^ cells were transfected with plasmids encoding for FLAG-tagged NICD1 wild-type (wt), FLAG-tagged NICD1 NNAA mutant, and/or HA-tagged ubiquitin (HA-Ub), HA-tagged ubiquitin mutated on lysine 63 to arginine (HA-Ub K63R), HA-tagged ubiquitin mutated on lysine 48 to arginine (HA-Ub K48R) or HA-tagged ubiquitin mutated on lysine 11 to arginine (HA-Ub K11R). Cells were treated with 20 μM of MG132 for 6 hours to block the activity of the proteasome. WCE were subjected to FLAG immunoprecipitation (FLAG-IP) and the immunoprecipitates were analyzed by Western blotting versus FLAG or HA. **B** Phoenix^TM^ cells were transfected with plasmids encoding for FLAG-tagged NICD1 wild-type (wt), FLAG-tagged NICD1 NNAA mutant, and/or HA-tagged ubiquitin (HA-Ub), HA-tagged ubiquitin mutated on all lysines to arginines with exception of lysine 63 (HA-Ub K63 only), HA-tagged ubiquitin mutated on all lysines to arginines with exception of lysine 48 (HA-Ub K48 only) or HA-tagged ubiquitin mutated on all lysines to arginines with exception of lysine 11 (HA-Ub K11 only). Cells were treated with 20 μM of MG132 for 6 hours to block the activity of the proteasome. WCE were subjected to FLAG immunoprecipitation (FLAG-IP) and the immunoprecipitates were analyzed by Western blotting versus FLAG or HA.

**Fig. S10 Relationship between hypoxia and FBXW7.** **A** Reduced interaction of the NICD1 NNAA mutant with FBXW7. Phoenix^TM^ cells were transfected with plasmids encoding FLAG-tagged NICD1 wild-type (wt), FLAG-tagged NICD1 NNAA mutant and/or HA-tagged FBXW7. After 3 hours of treatment with 10 μM MLN4924 to block protein NEDDylation as a tool to stabilize the proteins, whole cell extract (WCE) were prepared and subjected to FLAG immunoprecipitation (FLAG-IP). Immunoprecipitates were analyzed by Western blotting versus FLAG or HA. **B and C** FBXW7 protein expression is not influenced by hypoxia induction. Beko cells were kept for **B** 12 hours in hypoxia (1% O_2_) or normoxia (5% O_2_) as a control or, alternatively, **C** treated with 0.5 mM DMOG or with DMSO as a control for 24 hours. WCE were analyzed by Western blotting versus FBXW7 or GAPDH as a loading control.

**Fig. S11 USP10 knockdown (KD) leads to downregulation of Notch target genes in Beko cells.** Beko cells were infected with shRNAs directed against *Usp10* (*Usp10* *KD*) or scramble (SCR) as a control. Upon RNA extraction, samples were analysed by deep sequencing. **A** Heat map showing the sample wise correlation of the different RNA-Seq samples in Beko cells. Shown is the Pearson correlation coefficient (PCC) based on the union of all significantly deregulates genes (log2FC > 1 or < -1 and adjusted p-value < 0.05) upon USP10 KD/scrambled. **B** GSEA plot of the term “Notch signaling pathway” using DESeq2’s Wald-test statistic for USP10 KD/scramble. **C** Box plot showing the quantification of the heat map shown in Fig. 6F. *Bona fide* Notch target genes are significantly downregulated upon USP10 knockdown (KD; KD/scramble control). Wilcoxon rank sum tests (****P <* 0.001, NS = not significant).

**Fig. S12 Model depicting the role of FIH-mediated NICD1 hydroxilation.** **A** Under normoxic conditions, FIH hydroxylates NICD1 and this allows recruitment of USP10 which keeps the ubiquitination of the NICD1 occurring via K11, K48 and K63 under control. This would finally promote stability of the NICD1 protein and expression of Notch target genes. **B** Under hypoxia, the inactivation of FIH leads to reduced NICD1 hydroxylation. In addition, downregulation of USP10 and its reduced interaction with the NICD1 lead to a strong increase in NICD1 ubiquitination mainly via K63 but also via K11 and K48, resulting in decreased NICD1 stability and reduced gene expression. “REG-Ub” indicates the regulatory ubiquitination via K11 and K63; “DEG-Ub” indicates the degradative ubiquitination via K48.

**SUPPLEMENTARY TABLES**

**Table S1. Alignment statistics of the RNA-Seq and ChIP-Seq data analyzed in this study.**

Alignment statistic of the individual RNA-Seq and ChIP-Seq files showing the number of reads, number of aligned reads and percentage of alignment per file.

**Table S2. Differential gene expression profile as identified from the RNA-Seq data analyzed in this study.** Beko cells were treated with 10 μg/mL GSI [3], 0.5 mM DMOG or with DMSO as a control for 24 hours. Alternatively, cells were kept for 12 hours in hypoxia (1% O_2_) or normoxia (5% O_2_) as a control. Alternatively, Beko cells were infected with shRNAs directed against *Usp10* (*Usp10* KD) or scramble (SCR) as a control. RNA was purified and analyzed by deep sequencing. Tables showing the DESeq2 results for GSI/DMSO, DMOG/DMSO, hypoxia/normoxia, USP10 KD/scrambled and the normalized read counts per gene.

**Table S3. KEGG analysis of genes commonly upregulated by hypoxia or DMOG treatment in Beko cells.** Gene over-representation analysis based on the KEGG database showing for each significant (adjusted p-value < 0.05) enriched term the ID, Description, GeneRatio, BgRatio, p-value, adjusted p-value, geneIDs and the counts.

**Table S4. GO analysis of genes commonly upregulated by hypoxia or DMOG treatment in Beko cells.**

Gene over-representation analysis based on the GO database “Biological Pathways” showing for each significant (adjusted p-value < 0.05) enriched term the ID, Description, GeneRatio, BgRatio, p-value, adjusted p-value, geneIDs and the counts.

**Table S5. GSEA analysis of genes deregulated by DMOG treatment in Beko cells.**

Gene set enrichment analysis based on the GO database “Biological Pathways” showing for each significant (adjusted p-value < 0.05) enriched term the ID, Description, setSize, enrichmentScore, NES, p-value, adjusted p-value, q-value, rank, leading edge and core enrichment.

**Table S6. GSEA analysis of genes deregulated by hypoxia treatment in Beko cells.**

Gene set enrichment analysis (GSEA) based on the GO database “Biological Pathways” showing for each significant (adjusted p-value < 0.05) enriched term the ID, Description, setSize, enrichmentScore, NES, p-value, adjusted p-value, q-value, rank, leading edge and core enrichment.

**Table S7. ChIP-Seq versus RBPJ in Beko cells.**

Genomic coordinates (chromosome, start & end) of RBPJ or HIF1α binding sites.

**Table S8. HIF1a target genes in Beko cells.**

List of genes deregulated upon treatment with DMOG or deregulated upon exposure to hypoxia (1% O_2_) and bound by HIF1α in Beko cells. Beko cells were treated with 0.5 mM DMOG or DMSO as a control for 24 hours. Alternatively, cells were kept for 12 hours in hypoxia (1% O_2_) or normoxia (5% O_2_) as a control.

**Table S9. GSEA analysis of genes deregulated by USP10 knockdown (KD) in Beko cells.**

Gene set enrichment analysis (GSEA) based on the GO database “Biological Pathways” showing for each significant (adjusted p-value < 0.05) enriched term the ID, Description, setSize, enrichmentScore, NES, p-value, adjusted p-value, q-value, rank, leading edge and core enrichment.

**Table S10. Primers used for cloning purposes, CRISPR/Cas9, qPCR, ChIP experiments and sequence of the shRNA.**

| **Cloning** |  | |
| --- | --- | --- |
| hFIH NotI fw_2 | 5’- GCG GCC GCG CGG CGA CAG CGG CGG AGG CTG -3’ | |
| hFIH XbaI rv | 5’- TCT AGA CTA GTT GTA TCG GCC CTT GAT C -3’ | |
| hFIH NotI fw_1 | 5’- GCG GCC GCT ATG GCG GCG ACA GCG GCG -3’ | |
| hFIH NotI rv | 5’- TGG AGC GGC CGC CTA GTT GTA TCG GCC CTT G -3’ | |
| FBXW7 NotI fw | 5’- GCG GCC GCA ATC AGG AAC TGC TCT CTG TGG -3’ | |
| FBXW7 EcoRI rv | 5’- GAA TTC TCA TTT CAT GTC CAC ATC -3’ | |
|  |  | |
| **CRISPR/Cas9 guides** |  | |
| hHIF1AN guide #1 | 5’- CTC TGA CTC AGA CGC GGA AT -3’ | |
| hHIF1AN guide #2 | 5’- GGA AGC TAT AAC TGC GCA AC -3’ | |
| hHIF1AN guide #3 | 5’- CAG CGT GCA ATA CTA GCG CT -3’ | |
| hN1 guide #1 | 5´- CCC ACG TCG CTG CCA TCC TCG CT -3’ | |
| hN1 guide #2 | 5´- GGG AGG CAT GCC GCC GCT CC -3’ | |
| hN1 guide #3 | 5´- GCG GCG GCA TGC CTC CCC AC -3’ | |
|  |  | |
| **CRISPR/Cas9 screening** |  | |
| hHIF1AN screen2 fw | 5’- TCC GGA ATA GGC GGA GCT TCC -3’ | |
| hHIF1AN screen rv | 5’- TAT CTC CTC GCC TTT CTC TCC -3’ | |
| hN1 screen fw | 5´- ACT CTG AGC CTC ACT AGT GC -3’ | |
| hN1 screen rv | 5´- GCT TTT CCC TCT CCA TGC TG -3’ | |
|  |  | |
| **Mutagenesis** |  | |
| hFIH D201A fw | 5’- GTG ACA CCT GCT CAC TAT GCC GAG CAG CAG AAC -3’ | |
| hFIH D201A rev | 5’- GTT CTG CTG CTC GGC ATA GTG AGC AGG TGT CAC -3’ | |
| hFIH D201A_H199A fw | 5’- GGA AAT GTG ACA CCT GCT GCC TAT GCC GAG CAG CAG AAC -3’ | |
| hFIH D201A_H199A rev | 5’- GTT CTG CTG CTC GGC ATA GGC AGC AGG TGT CAC ATT TCC -3’ | |
|  |  | |
| **shRNA** |  | |
| Usp10 ShRNA | 5’- GCA CAG CCT ACC TCC TAT ATT -3’ | |
|  |  | |
| **Real time PCR** |  | |
|  | *Mus musculus* | Probe |
| Hprt fw | 5’- GGA GCG GTA GCA CCT CCT -3’ | 69 |
| Hprt rv | 5’- AAC CTG GTT CAT CAT CGC TAA -3’ |  |
| Il2ra fw | 5’- CAA TGG AGT ATA AGG TAG CAG TGG -3’ | 89 |
| Il2ra rv | 5’- CAT CTG TGT TGC CAG GTG AG -3’ |  |
| Necab1 fw | 5’- CCA GTG GAT GAC CCA GAT AAA -3’ | 109 |
| Necab1 rv | 5’- AGG GGT TCA AGT TTC AGA TCC -3’ |  |
| Ngfr fw | 5’- ACT GAG CGC CAG TTA CGC -3’ | 26 |
| Ngfr rv | 5’- CGT AGA CCT TGT GAT CCA TCG -3’ |  |
| Ptrca fw | 5’- CAG CTC TCC TTG CCT TCT GA -3’ | 45 |
| Ptrca rv | 5’- CCT GGC TGT CGA AGA TTC C -3’ |  |
| Smad3 fw | 5’- GCC ACT GTC TGC AAG ATC C -3’ | 64 |
| Smad3 rv | 5’- AGC TAG GAG GGC AGC AAA T -3’ |  |
| Tbp fw | 5’- GGG GAG CTG TGA TGT GAA GT -3’ | 97 |
| Tbp rv | 5’- CCA GGA AAT AAT TCT GGC TCA T -3’ |  |
| Tgfb3 fw | 5’- CCC TGG ACA CCA ATT ACT GC -3’ | 25 |
| Tgfb3 rv | 5’- TCA ATA TAA AGG GGG CGT ACA -3’ |  |
| Uaca fw | 5’- TGT CAA TTC CAG AGA CAA ACA AA -3’ | 26 |
| Uaca rv | 5’- GGC ATC TCT GCA ACC GTA CT -3’ |  |
| Usp10 fw | 5’- ATA CGC ACA GTC CAG GAT GC -3’ | 49 |
| Usp10 rv | 5’- TCC TGC TTG GTT TTG GTG GT -3’ |  |
| Vegfa fw | 5’- AAA AAC GAA AGC GCA AGA AA -3’ | 1 |
| Vegfa rv | 5’- TTT CTC CGC TCT GAA CAA GG -3’ |  |
|  | *Homo sapiens* | Probe |
| HPRT fw | 5’- TGA CCT TGA TTT ATT TTG CAT ACC -3’ | 73 |
| HPRT rv | 5’- CAT CTC GAG CAA GAC GTT CA -3’ |  |
| TBP fw | 5’- TTT GCA GTG ACC CAG CAG -3’ | 67 |
| TBP rv | 5’- CGC TGG AAC TCG TCT CAC TA -3’ |  |
| HES4 fw | 5’- GCT CAG CTC AAA ACC CTC AT -3’ | 78 |
| HES4 rv | 5’- CTC ACG GTC ATC TCC AGG AT -3’ |  |
| HEYL fw | 5’- TCC CCA CTG CCT TTG AGA -3’ | 78 |
| HEYL rv | 5’- TTT CAA GTG ATC CAC CGT CA -3’ |  |
| JMJD1A fw | 5’- CCA GCC TCA AAG GAA GAC CT -3’ | 78 |
| JMJD1A rv | 5’- ACT GCA CCA AGA GTC GGT TT -3’ |  |
|  |  | |
| **ChIP** |  | |
|  | *Mus musculus* | Probe |
| Chrom X fw | 5’- GAG TTC CAG GGA CTG TCA CG -3’ | 68 |
| Chrom X rv | 5’- ATG GTG TCT ACT TGT AAG CCC AGT -3’ |  |
| Gapdh fw | 5’- GGG TTC CTA TAA ATA CGG ACT GC -3’ | 68 |
| Gapdh rv | 5’- CTG GCA CTG CAC AAG AAG A -3’ |  |
| Ptrca -4kb fw | 5’- CTG CAC TGT GGT CGC AGA -3’ | 60 |
| Ptrca -4kb rv | 5’- GGA GGC AGG TGT CCC TAA C -3’ |  |
| Uaca -25kb fw | 5’- GCA GGA AAC CCT CCT ACT CTT C -3’ | 73 |
| Uaca -25kb rv | 5’- GGC CAA CAG AAC GTG GAA -3’ |  |

**Table S11. Constructs cloned by PCR.**

| **Plasmid** | **Template** | **Primer Forward** | **Primer Reverse** | **Enzymes** | **Ligation** |
| --- | --- | --- | --- | --- | --- |
| pcDNA3.1 HA-hFIH | pcDNA FLAG-hFIH | hFIH NotI fw_2 | hFIH XbaI rv | NotI/XbaI | pcDNA3.1 HA |
| pMys BioFIH IRES GFP | pcDNA FLAG-hFIH | hFIH NotI fw_1 | hFIH NotI rv | NotI/NotI | pMy BioDdx5 |
| pcDNA3.1 HA-FBXW7 | pcDNA FLAG-FBXW7 | FBXW7 NotI fw | FBXW7 EcoRI rv | NotI/EcoRI | pcDNA3.1 HA |
