# Supplementary figures and images for: Hydroxylation of the NOTCH1 intracellular domain regulates Notch signaling dynamics
Source: Cell Death Dis. 2022 Jul 12;13(7):600. doi: 10.1038/s41419-022-05052-9 (PMC9276811; doi:10.1038/s41419-022-05052-9)

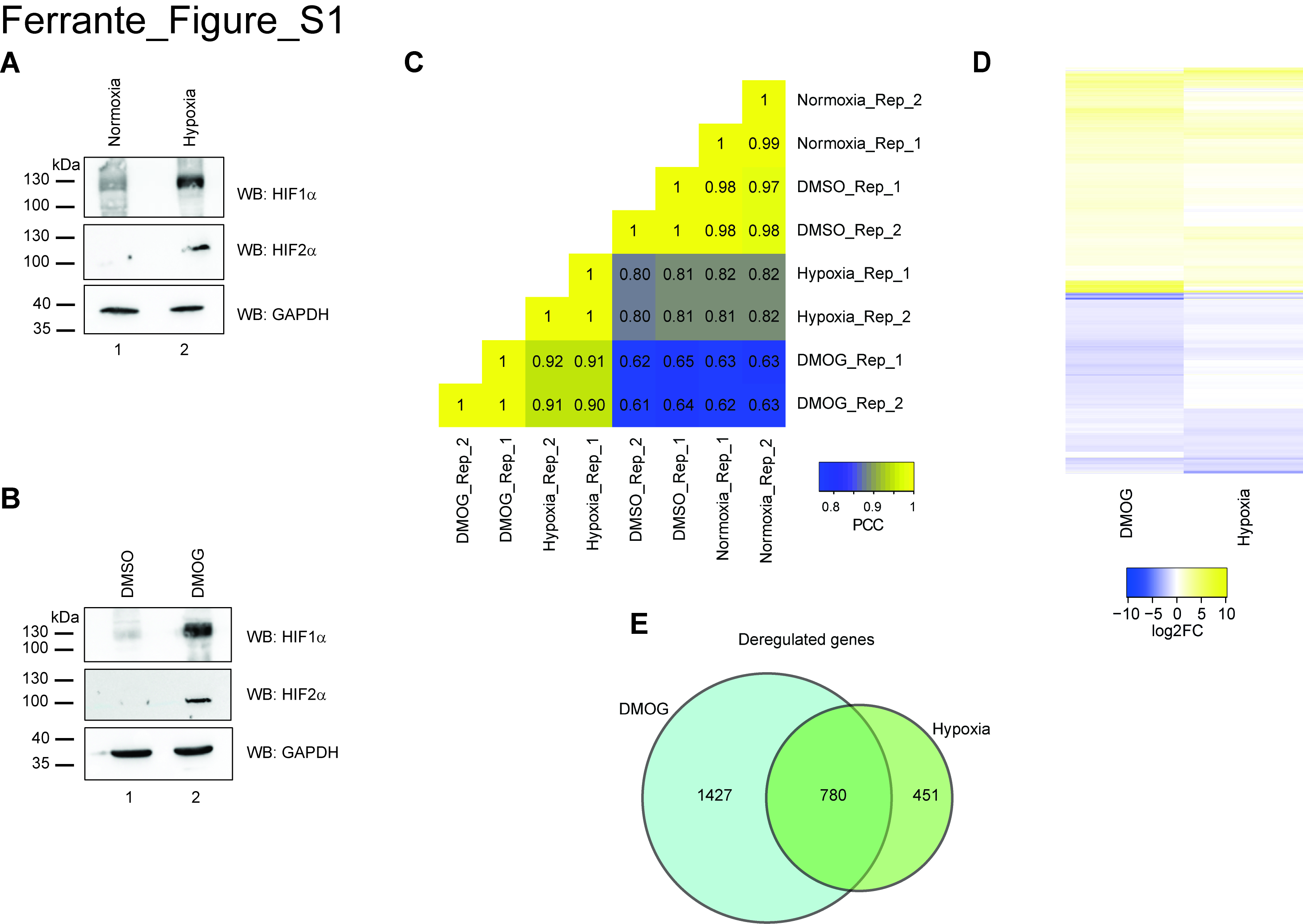

Supplement: Supplementary file 2 — Figure S1 [file 41419_2022_5052_MOESM2_ESM.tif]

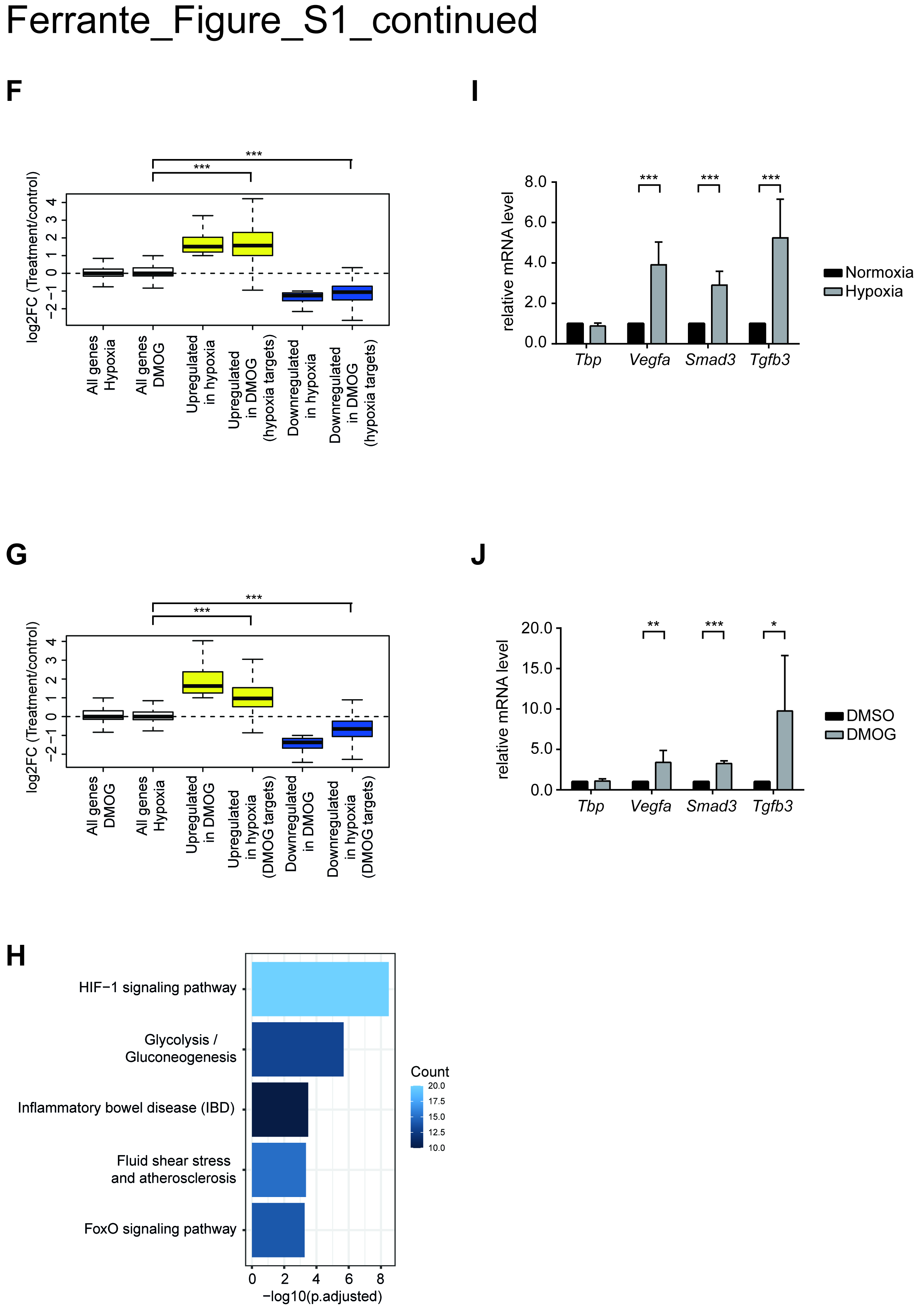

Supplement: Supplementary file 3 — Figure S1 continued [file 41419_2022_5052_MOESM3_ESM.tif]

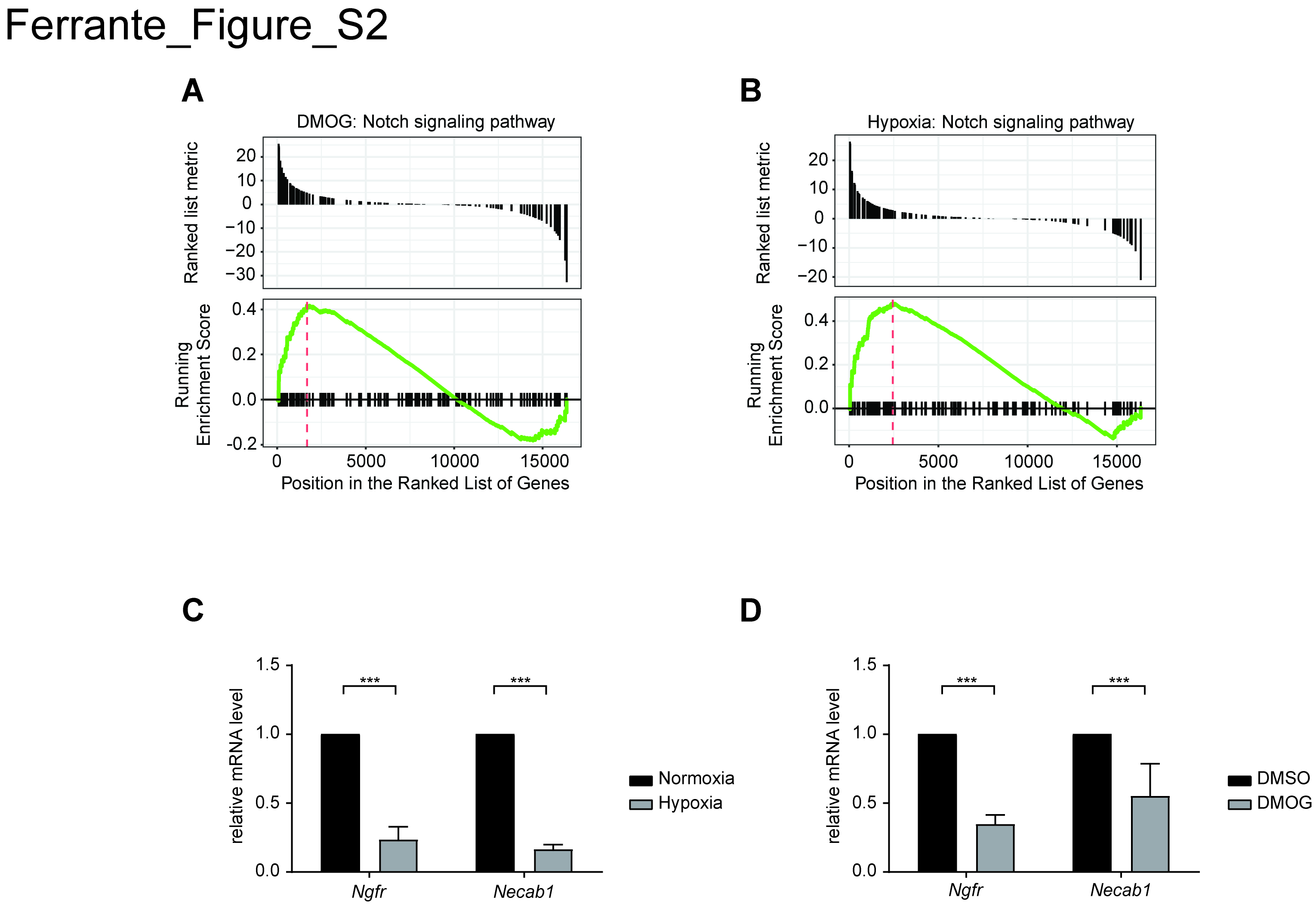

Supplement: Supplementary file 4 — Figure S2 [file 41419_2022_5052_MOESM4_ESM.tif]

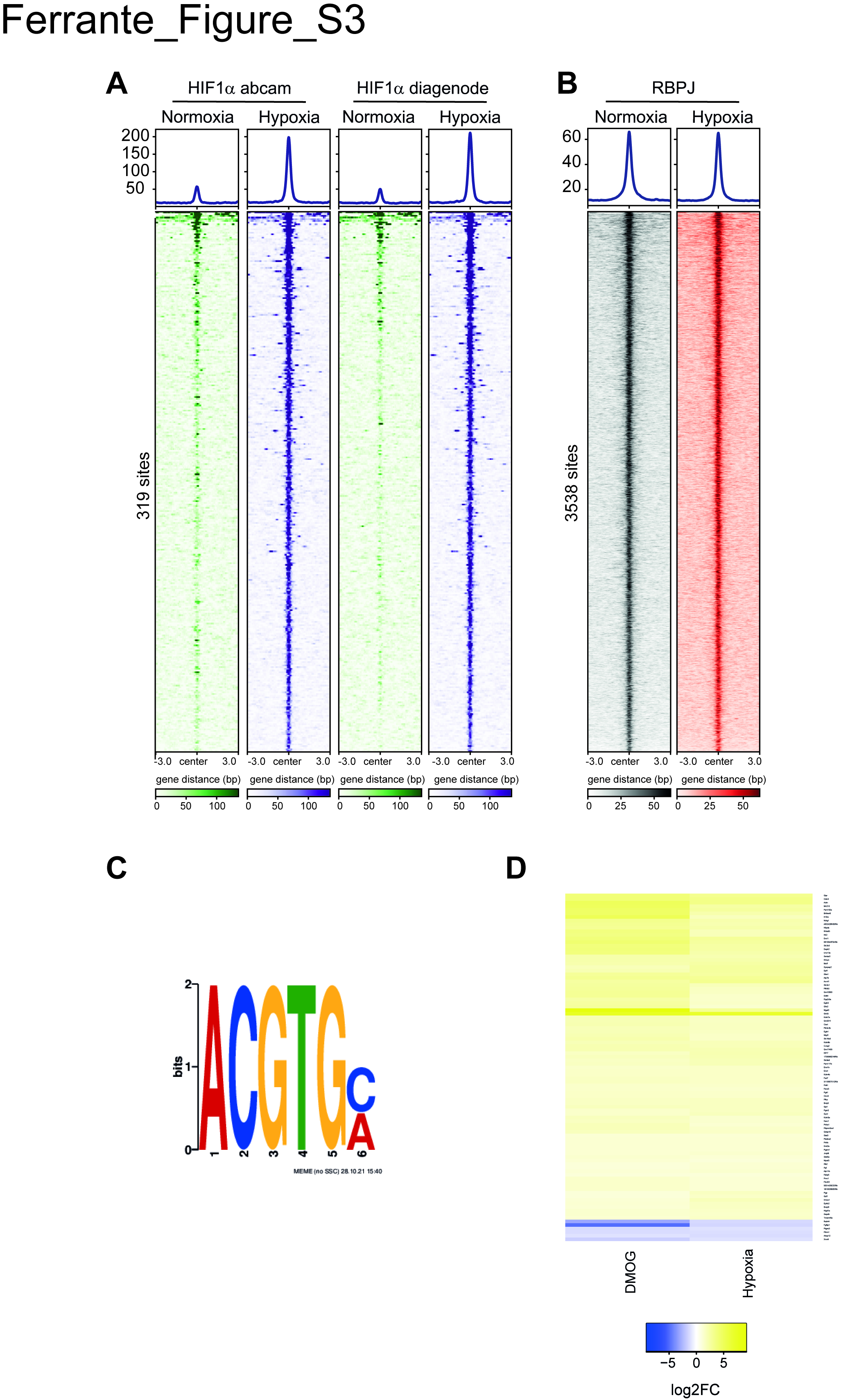

Supplement: Supplementary file 5 — Figure S3 [file 41419_2022_5052_MOESM5_ESM.tif]

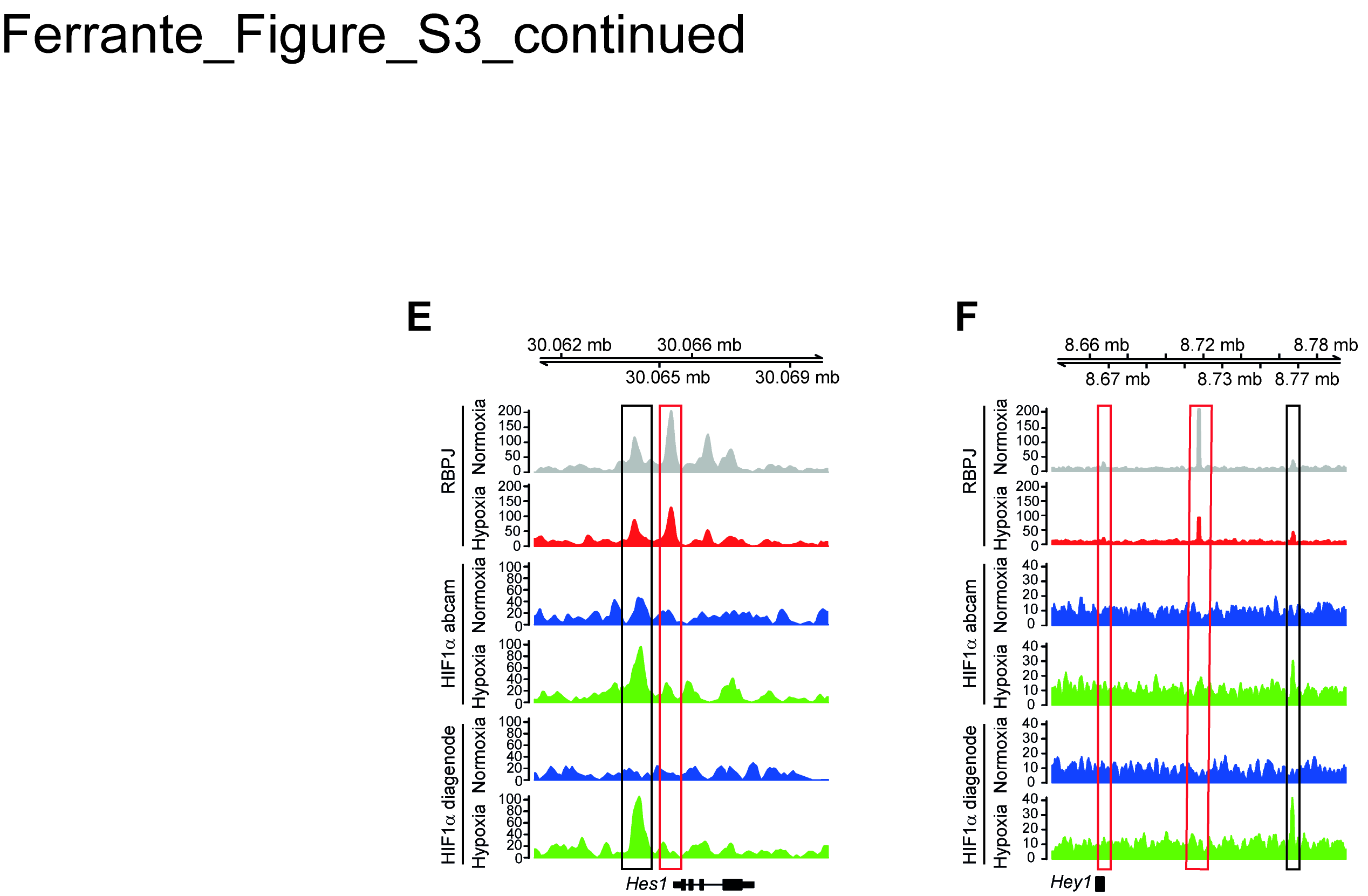

Supplement: Supplementary file 6 — Figure S3 continued [file 41419_2022_5052_MOESM6_ESM.tif]

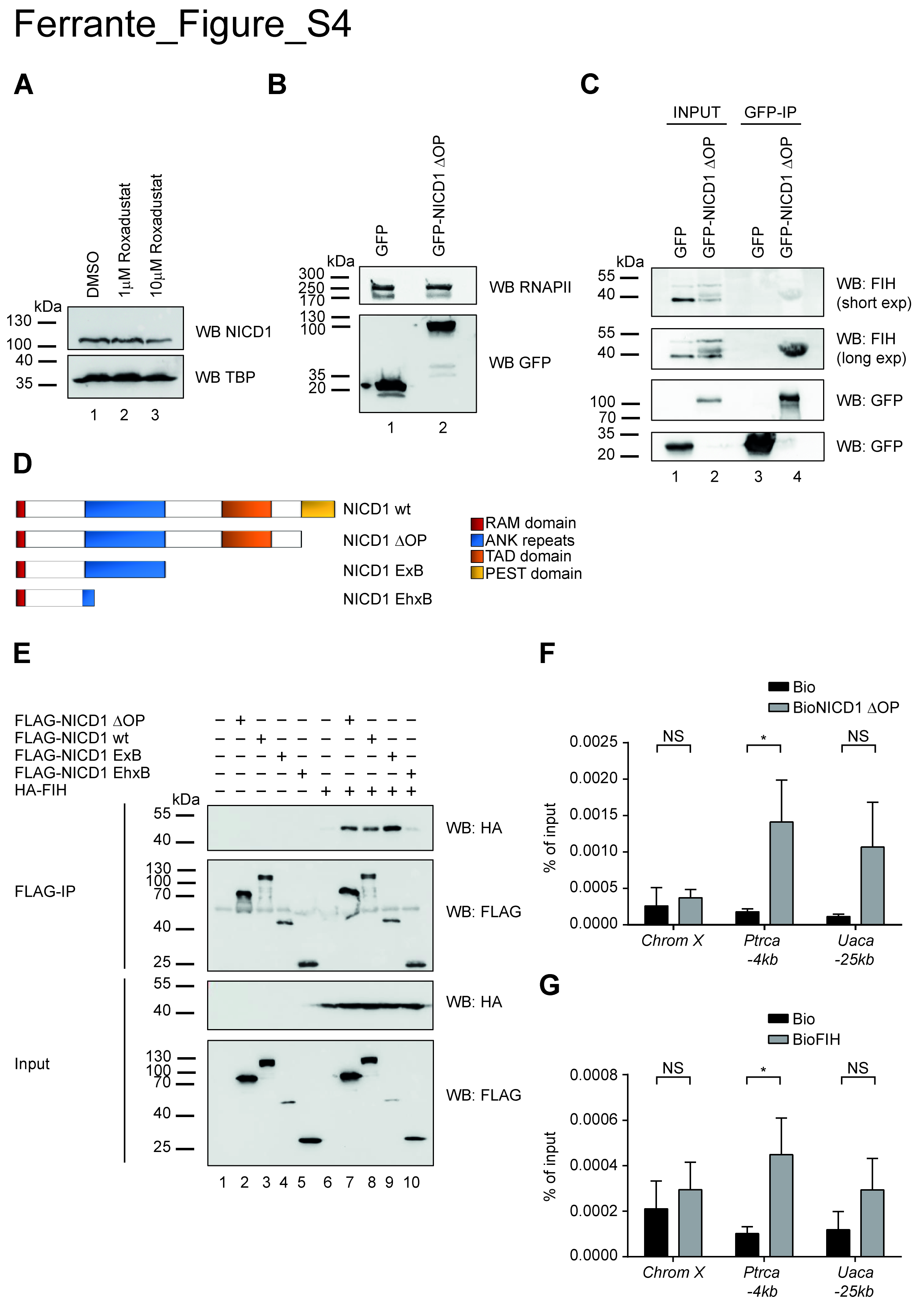

Supplement: Supplementary file 7 — Figure S4 [file 41419_2022_5052_MOESM7_ESM.tif]

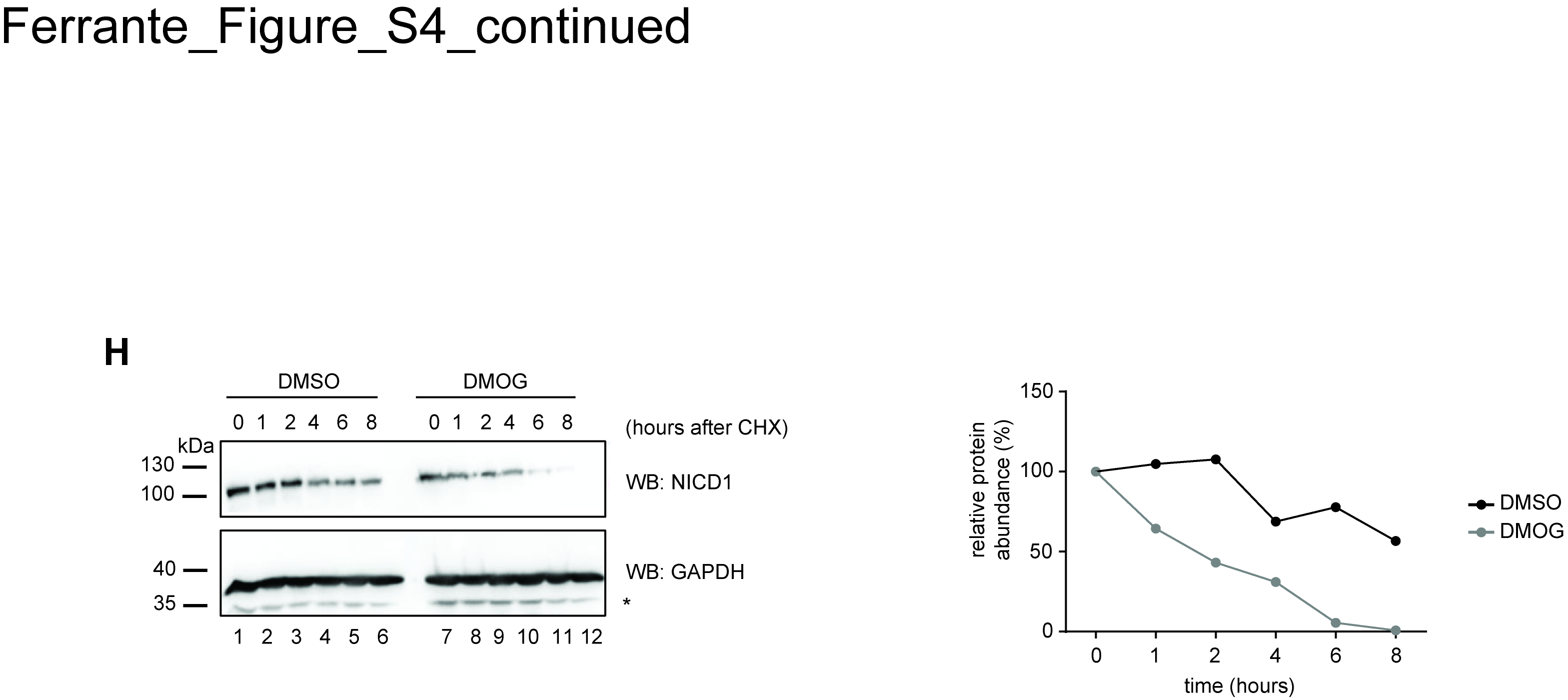

Supplement: Supplementary file 8 — Figure S4 continued [file 41419_2022_5052_MOESM8_ESM.tif]

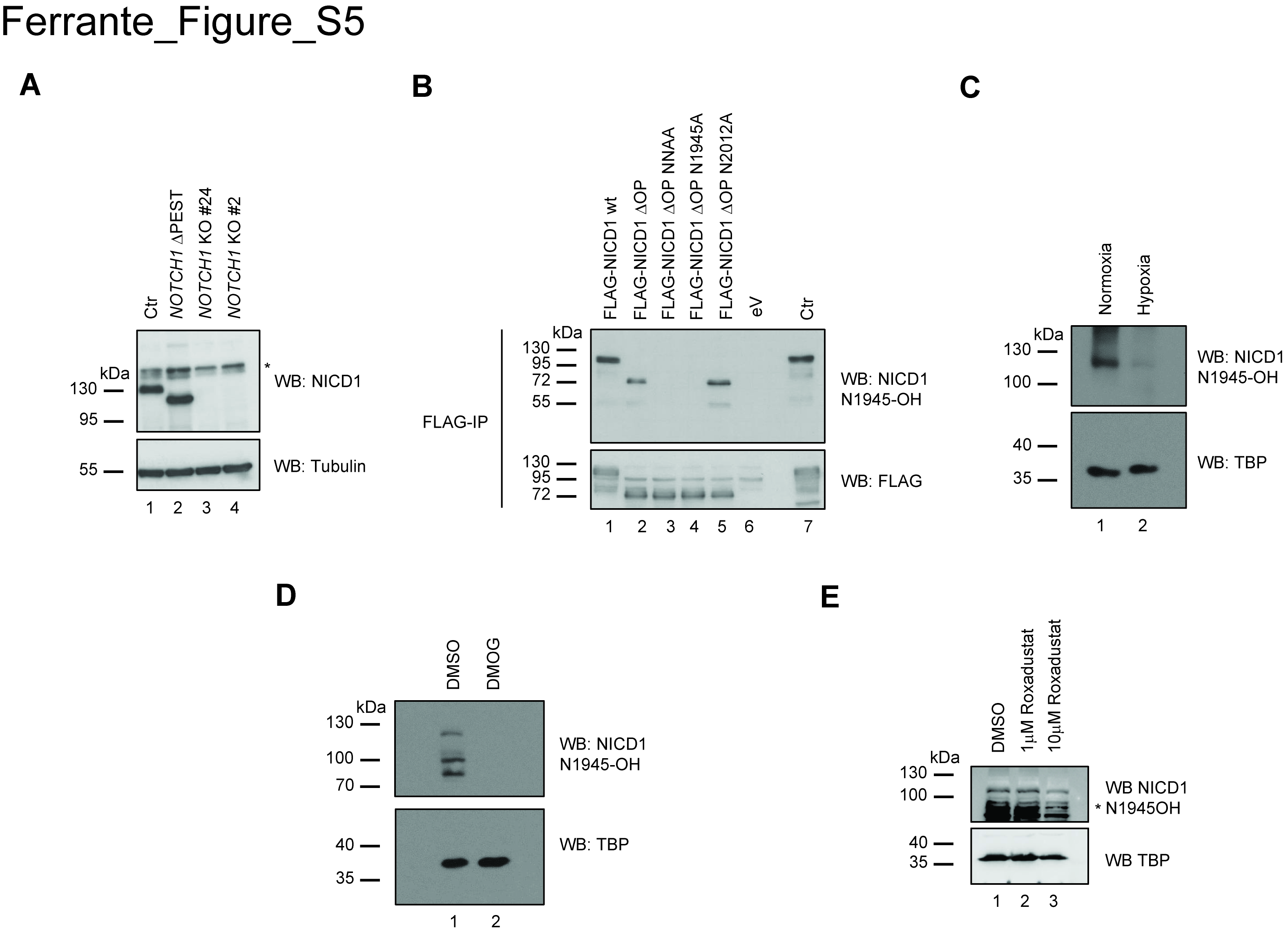

Supplement: Supplementary file 9 — Figure S5 [file 41419_2022_5052_MOESM9_ESM.tif]

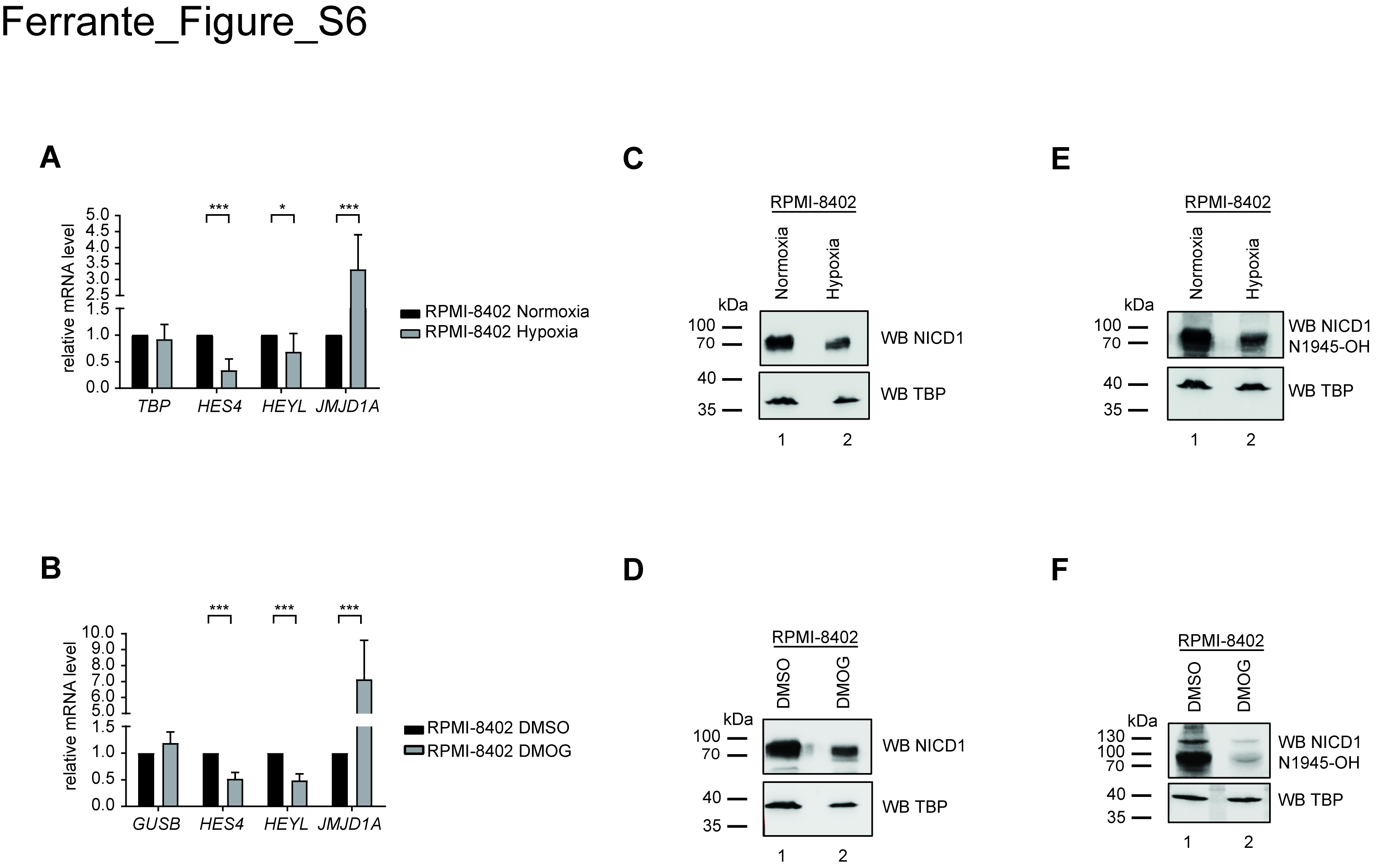

Supplement: Supplementary file 10 — Figure S6 [file 41419_2022_5052_MOESM10_ESM.tif]

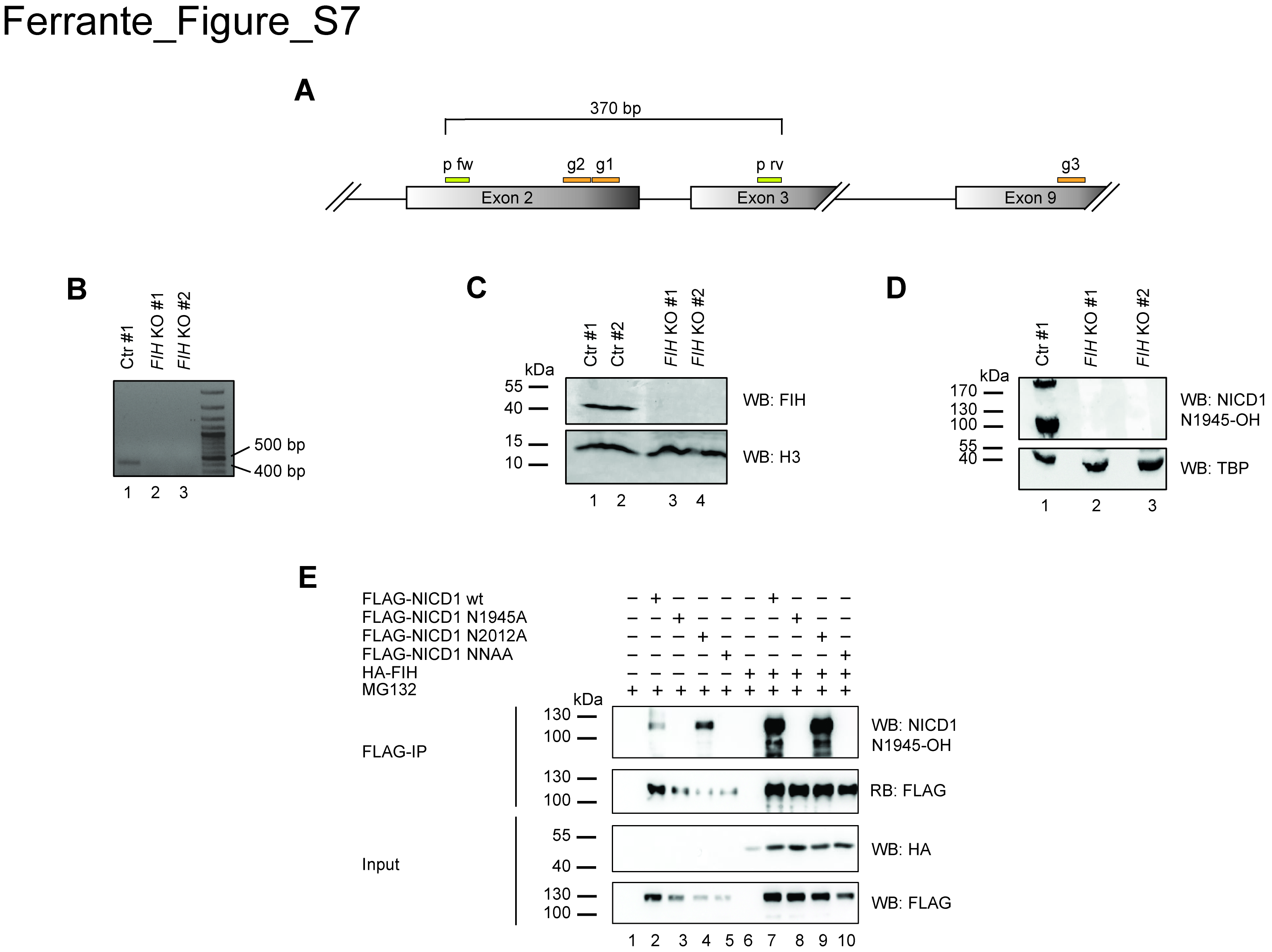

Supplement: Supplementary file 11 — Figure S7 [file 41419_2022_5052_MOESM11_ESM.tif]

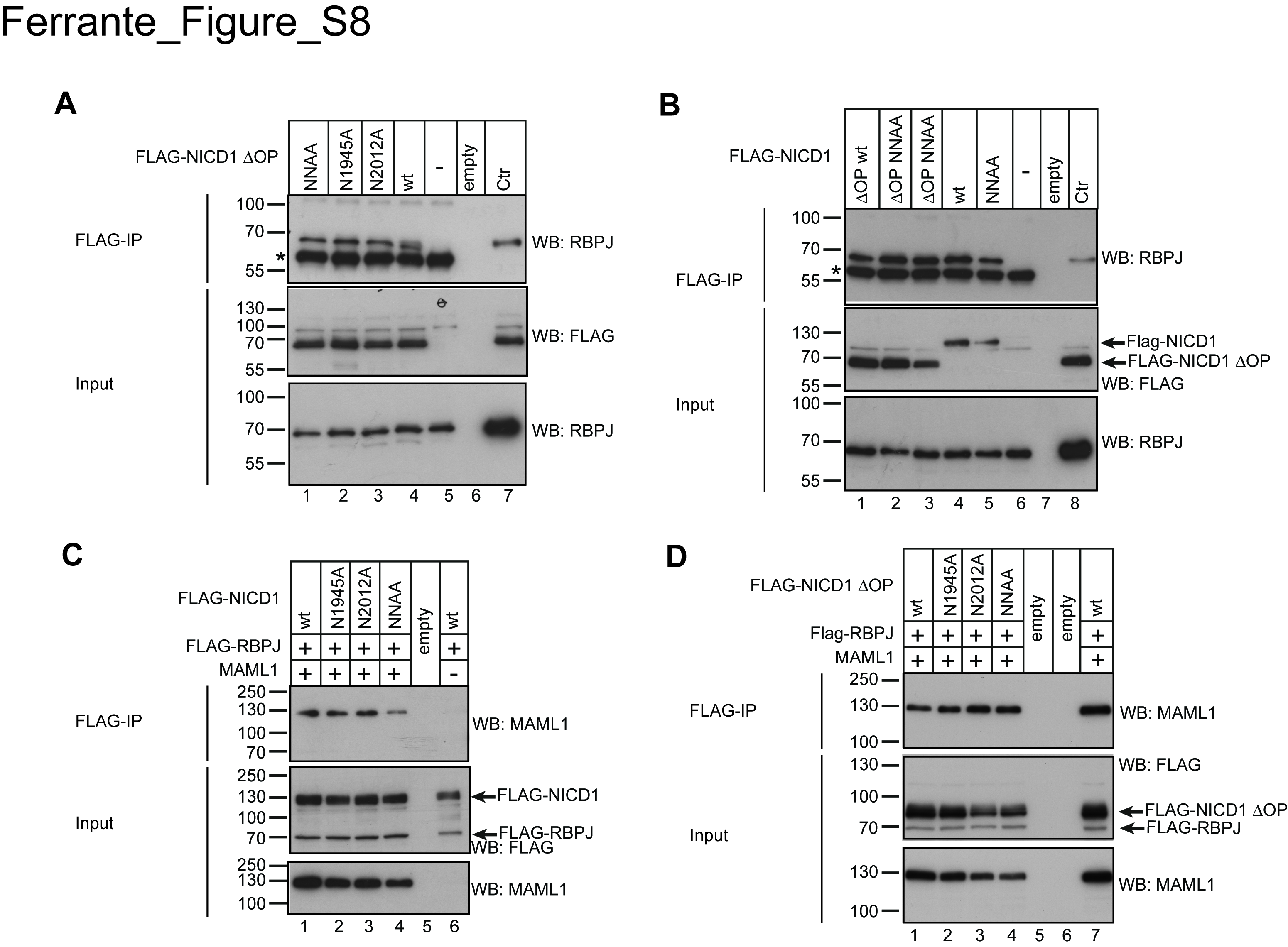

Supplement: Supplementary file 12 — Figure S8 [file 41419_2022_5052_MOESM12_ESM.tif]

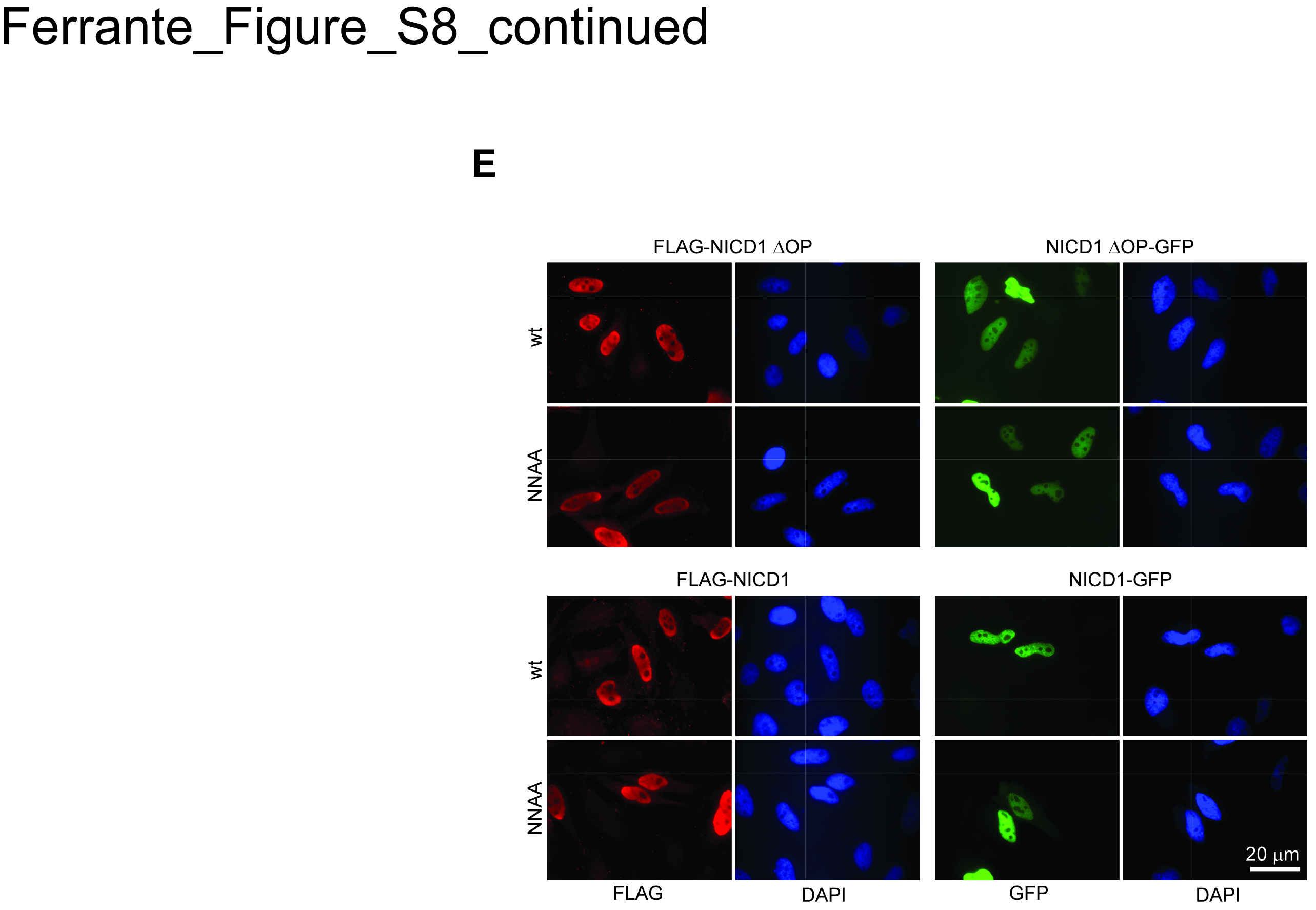

Supplement: Supplementary file 13 — Figure S8 continued [file 41419_2022_5052_MOESM13_ESM.tif]

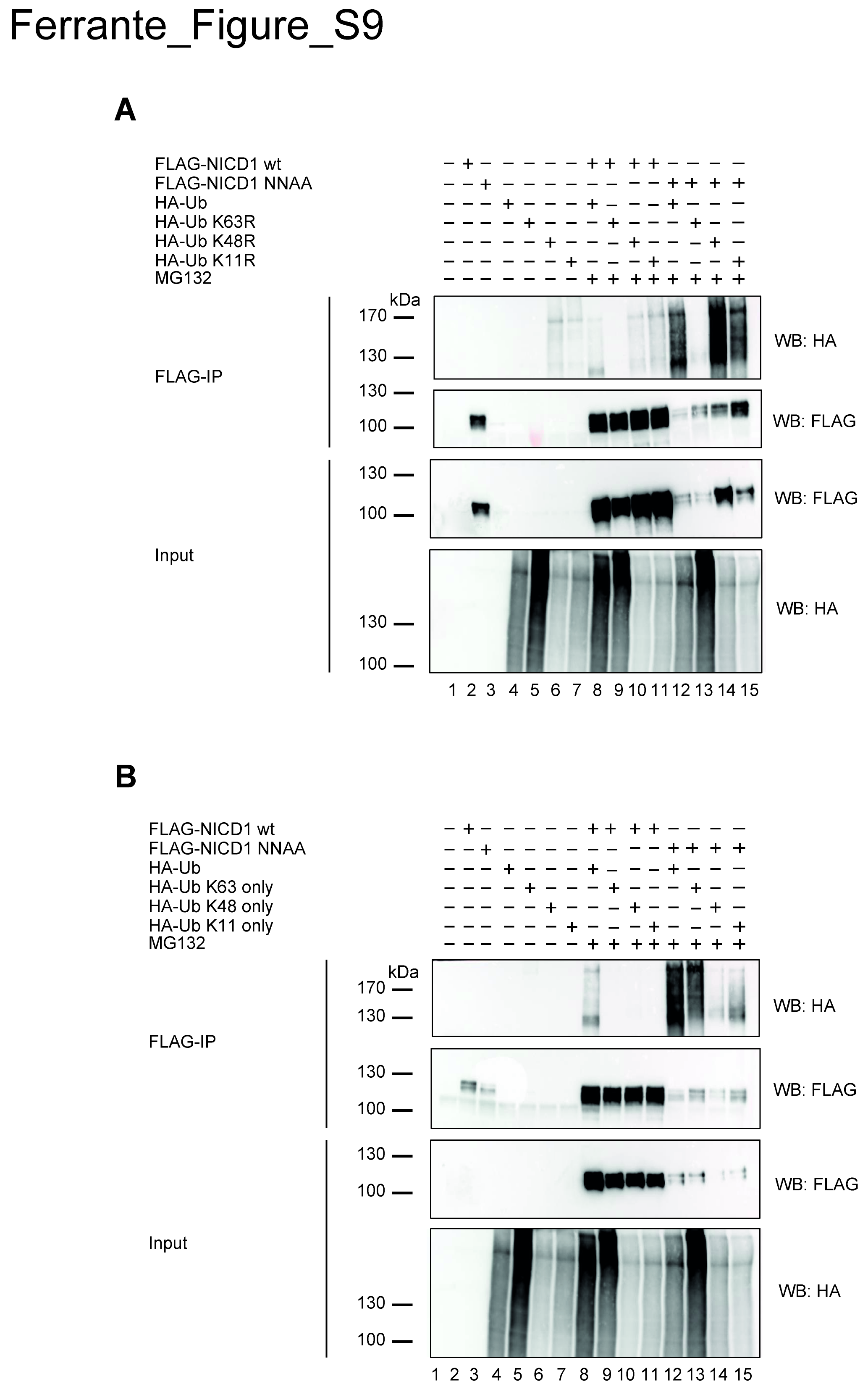

Supplement: Supplementary file 14 — Figure S9 [file 41419_2022_5052_MOESM14_ESM.tif]

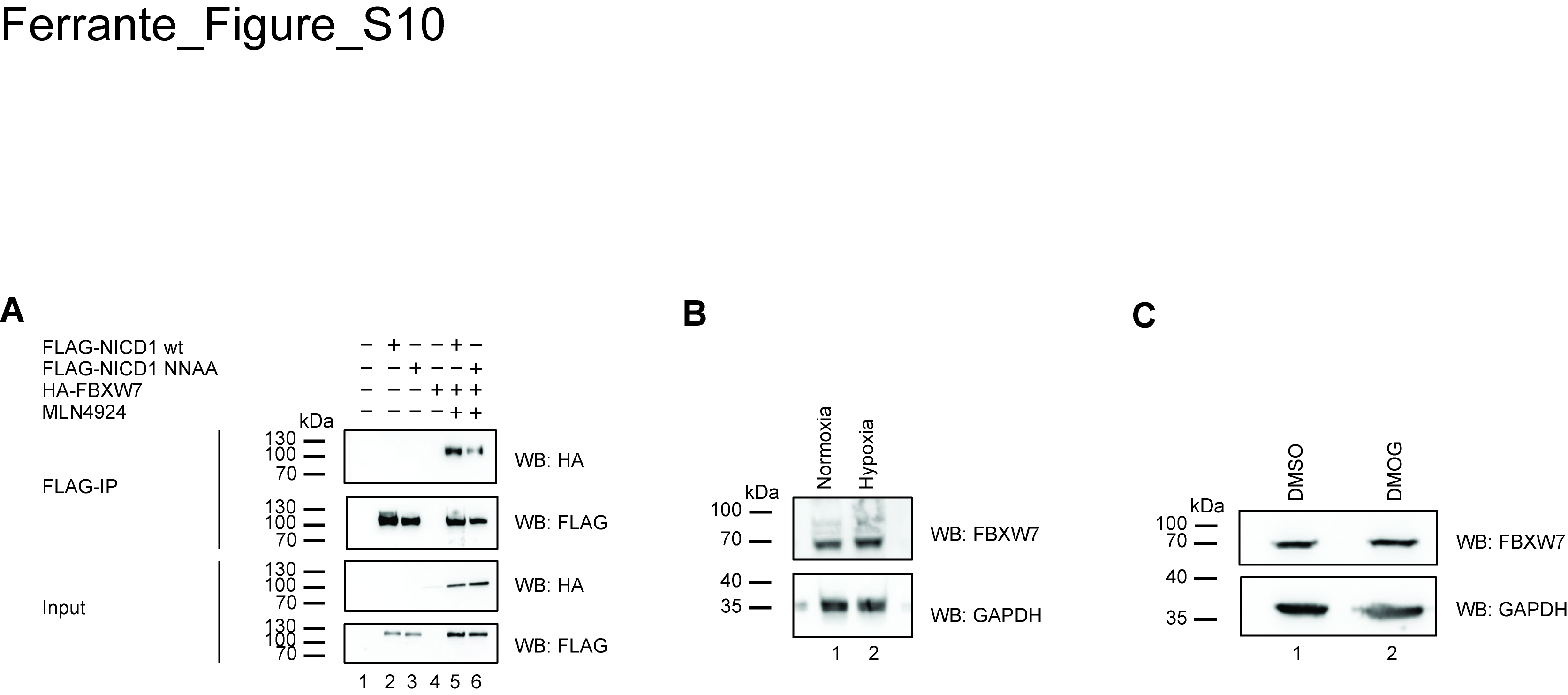

Supplement: Supplementary file 15 — Figure S10 [file 41419_2022_5052_MOESM15_ESM.tif]

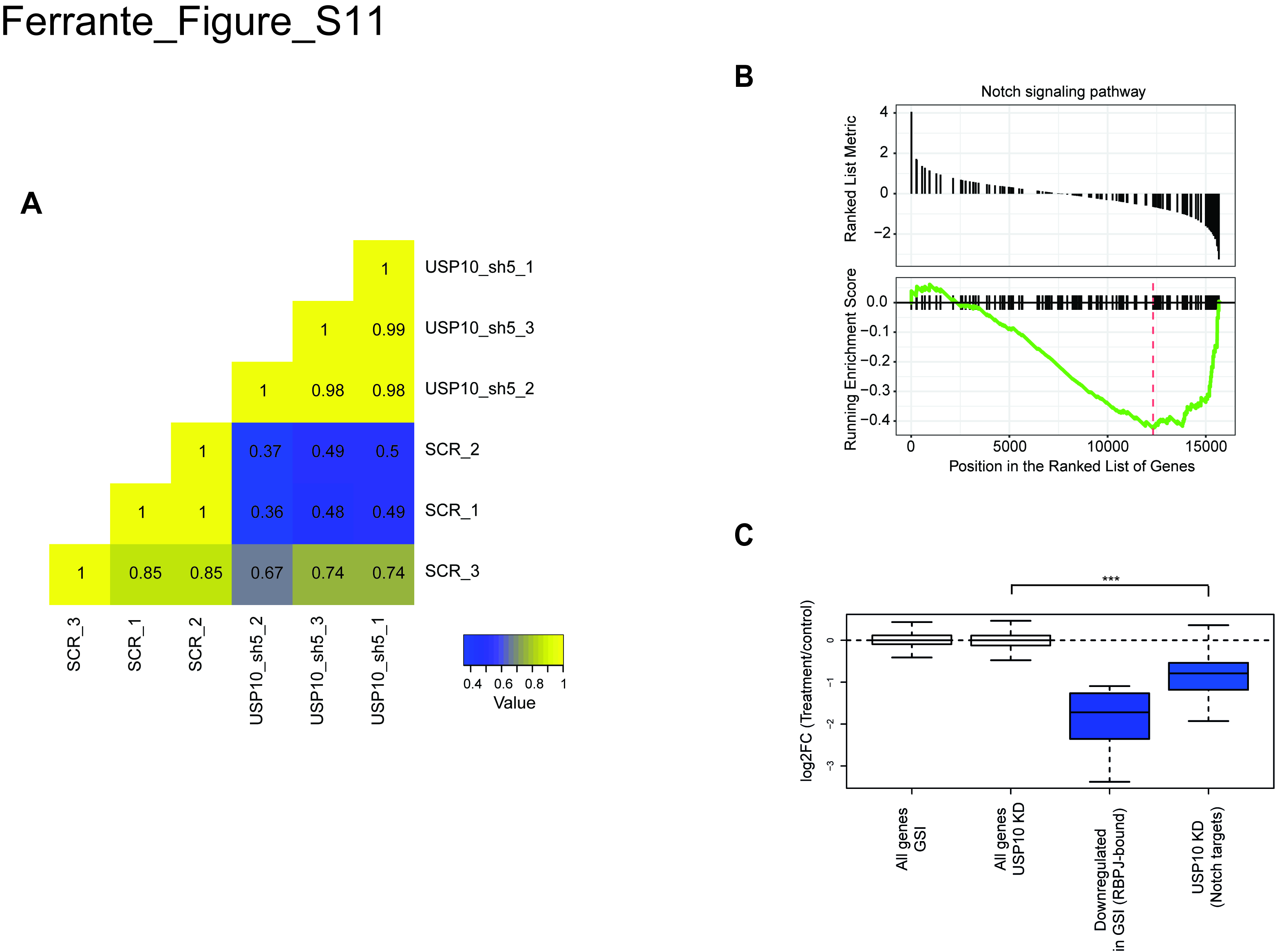

Supplement: Supplementary file 16 — Figure S11 [file 41419_2022_5052_MOESM16_ESM.tif]

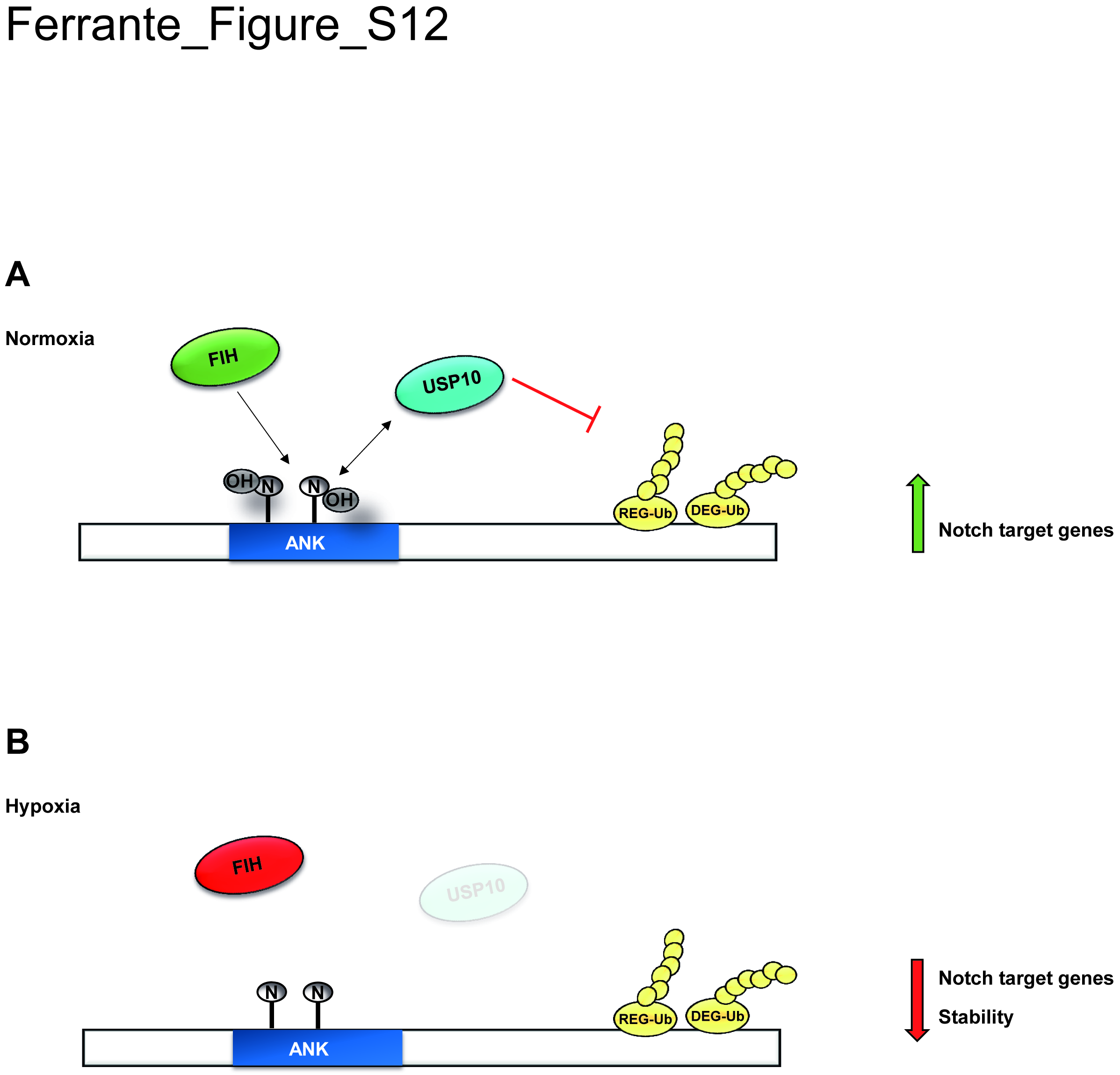

Supplement: Supplementary file 17 — Figure S12 [file 41419_2022_5052_MOESM17_ESM.tif]
