# Supplementary material for: Hydroxylation of the NOTCH1 intracellular domain regulates Notch signaling dynamics
Source: Cell Death Dis. 2022 Jul 12;13(7):600. doi: 10.1038/s41419-022-05052-9 (PMC9276811; doi:10.1038/s41419-022-05052-9)

Fig2A

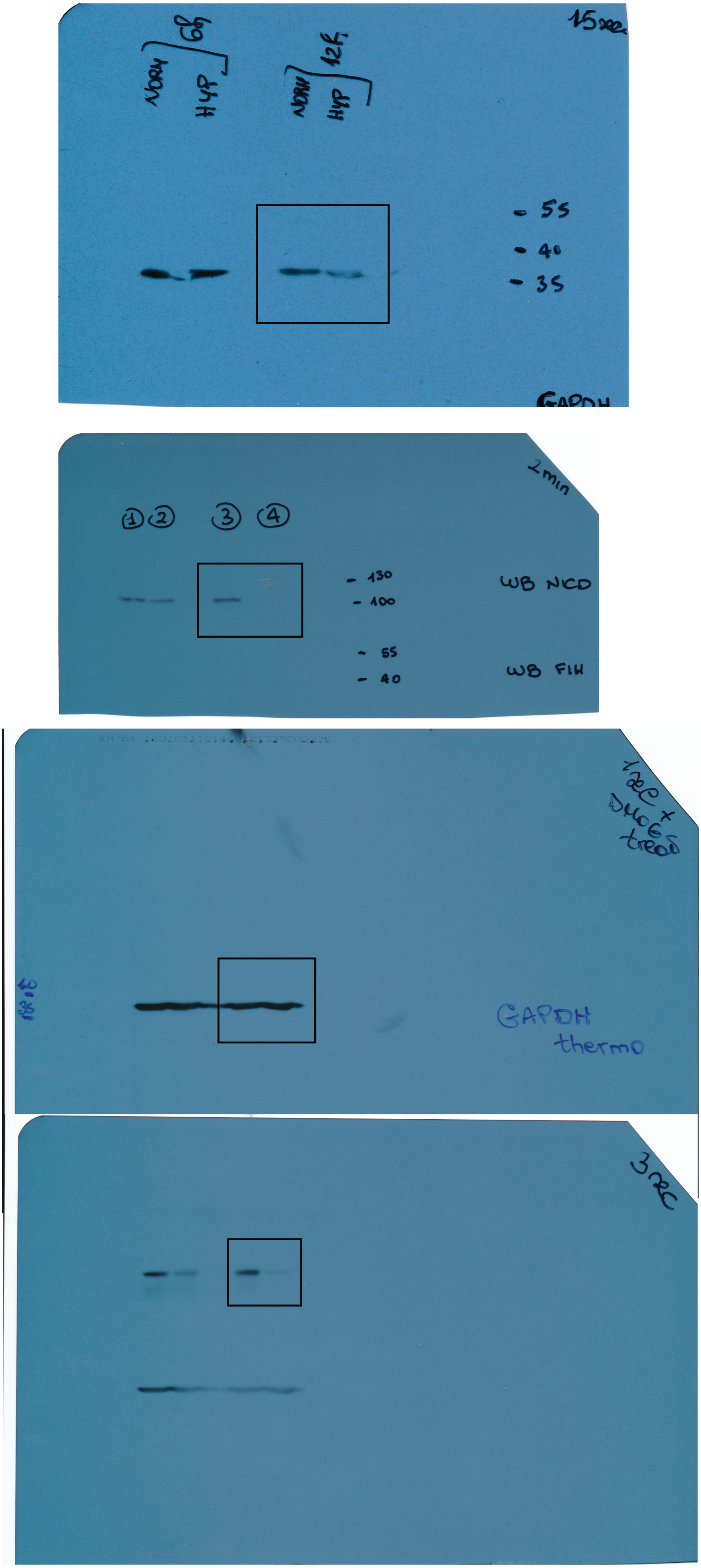
15sec
NORM
HYP
6h
NORM
HYP
12h
55
40
35
GAPDH
2min
1 2 3 4
130
100
WB NCD
55
40
WB FIH
GAPDH
thermo
3sec

Fig. new 2 B

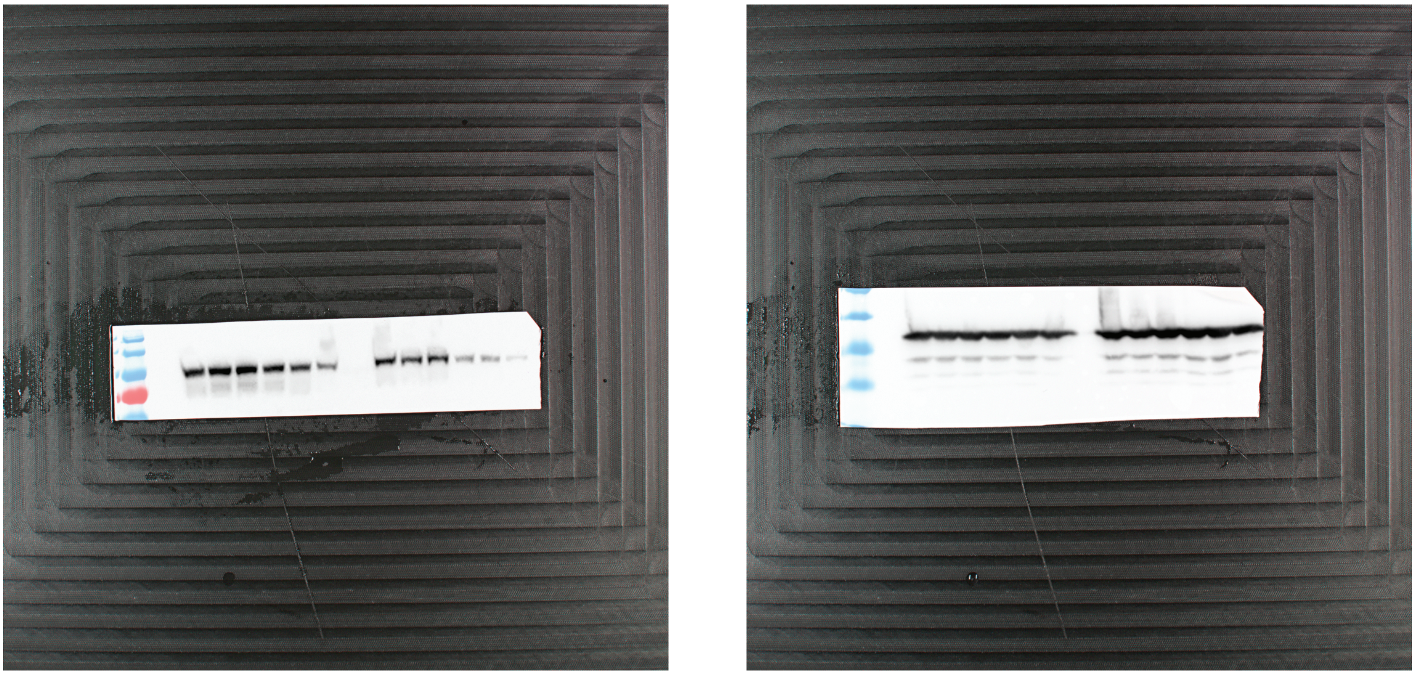

Fig2C

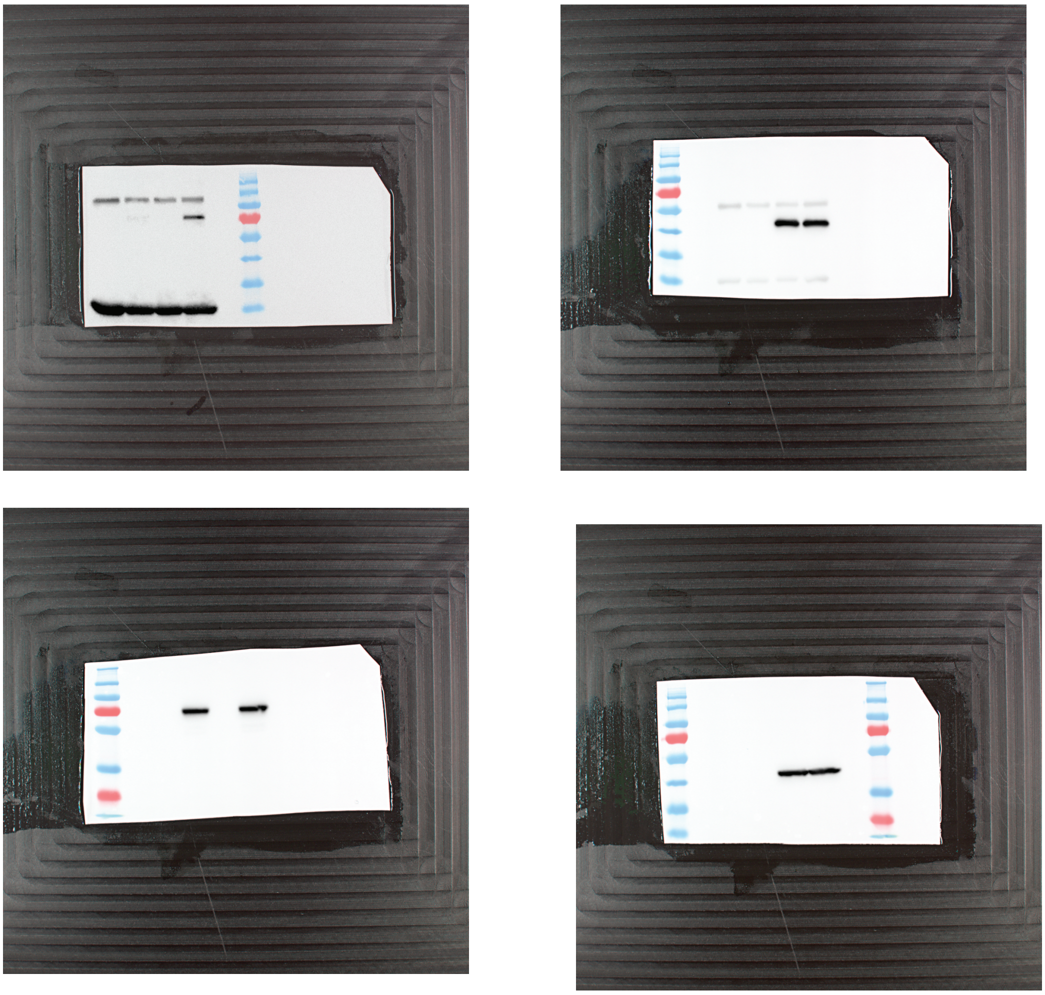

Fig3A

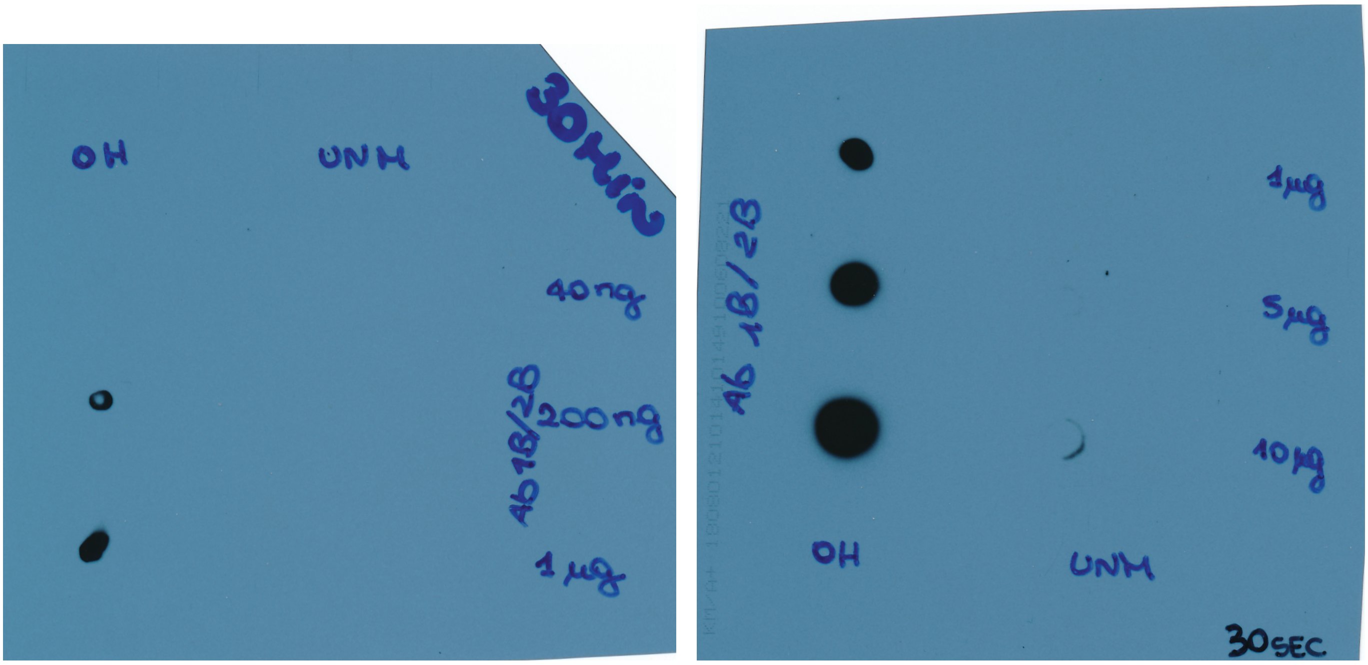
OH
UNM
30 min
40 ng
Ab 1B/2B
200 ng
1 µg
Ab 1B/2B
1 µg
5 µg
10 µg
OH
UNM
30 SEC

Fig3B

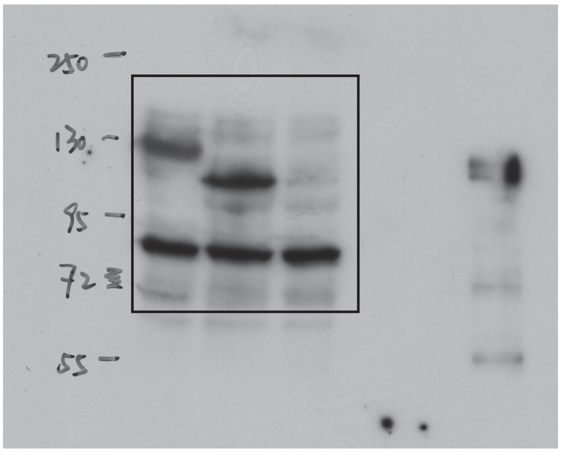
250
130
95
72
55

Fig3C

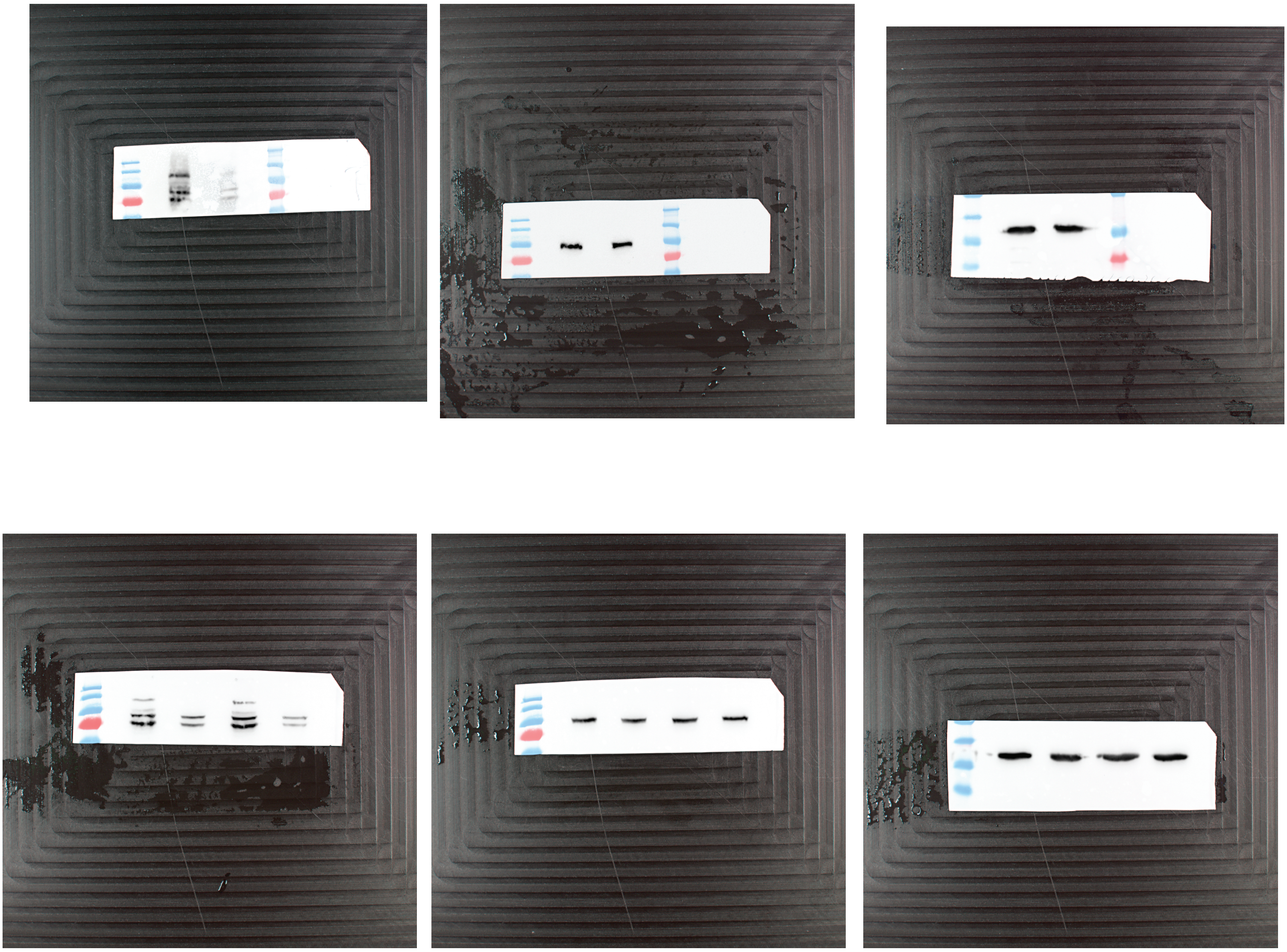

Fig3D

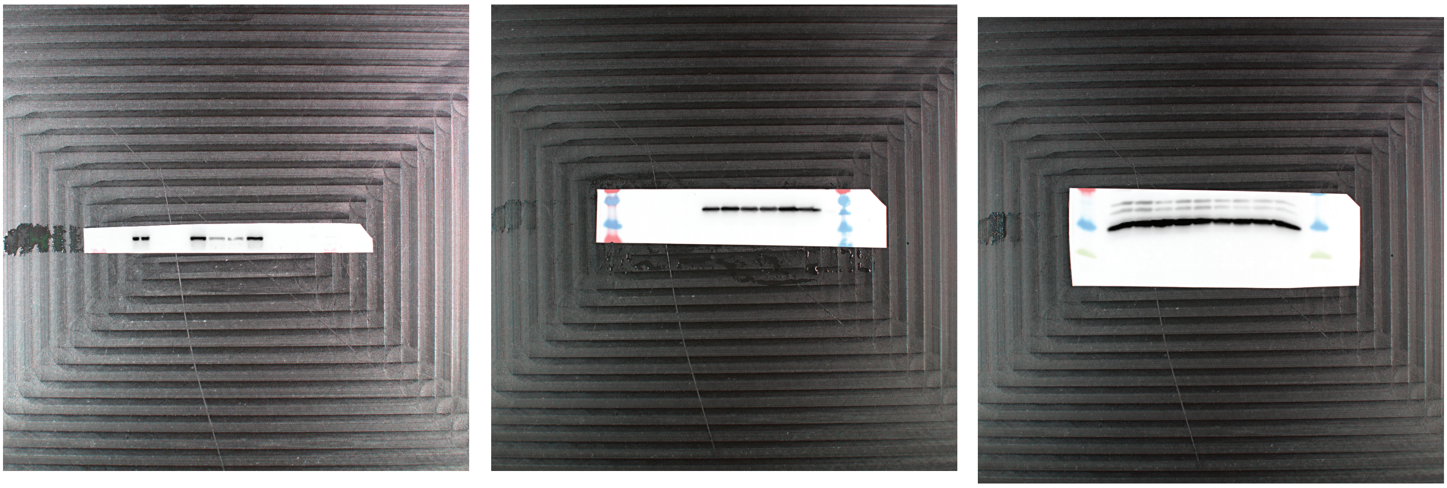

Fig3E

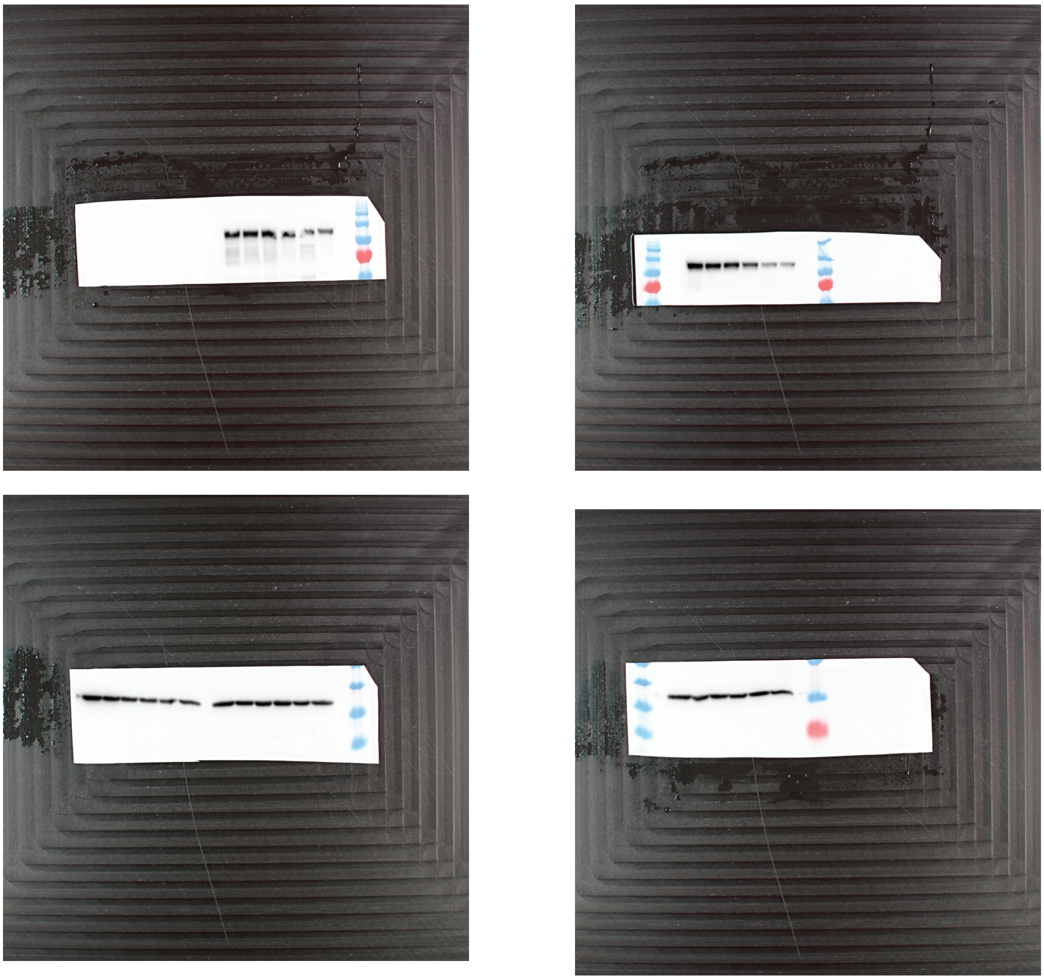

Fig5A

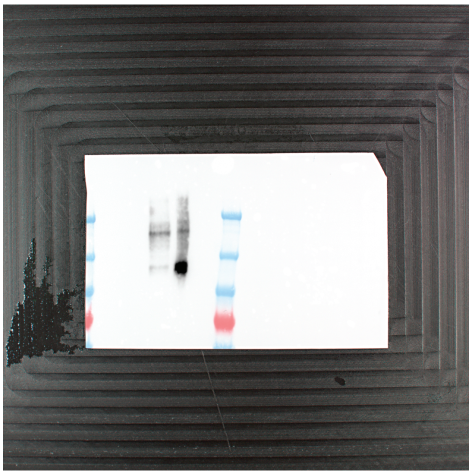

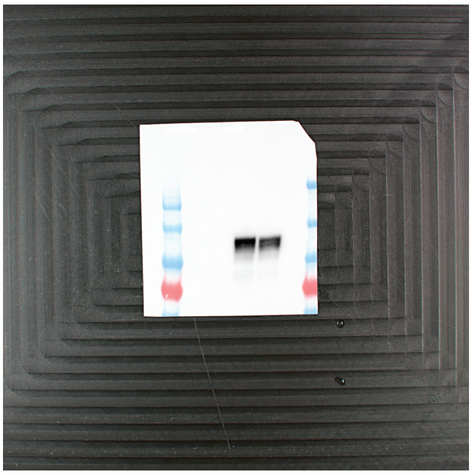

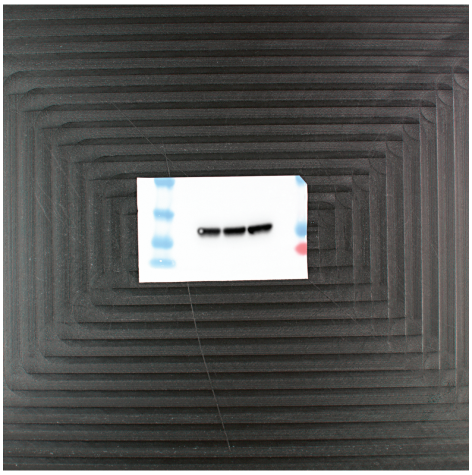

Fig5B

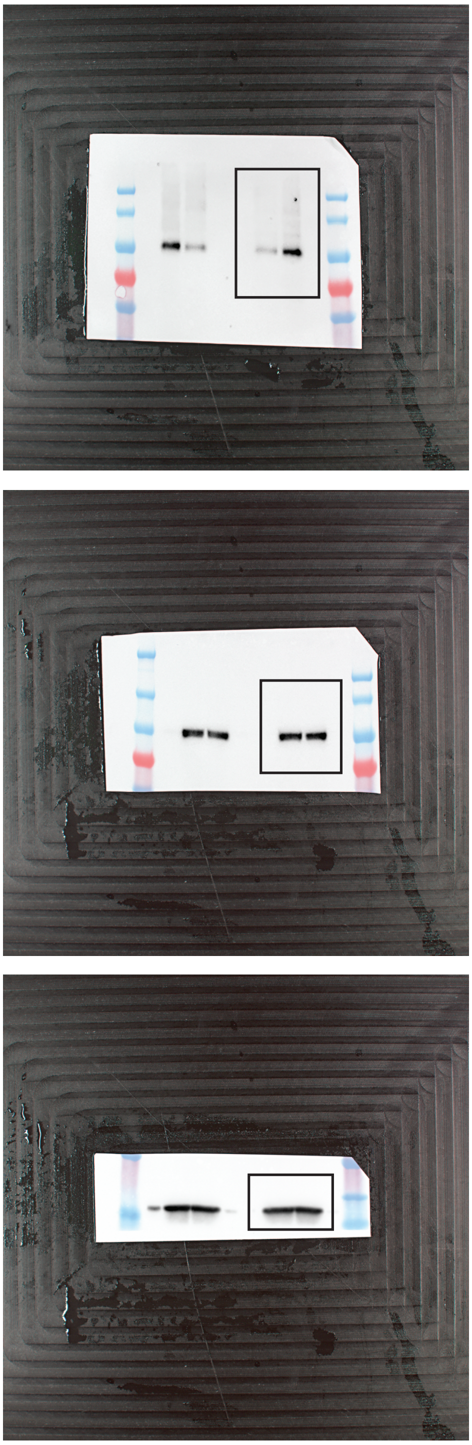

Fig5C

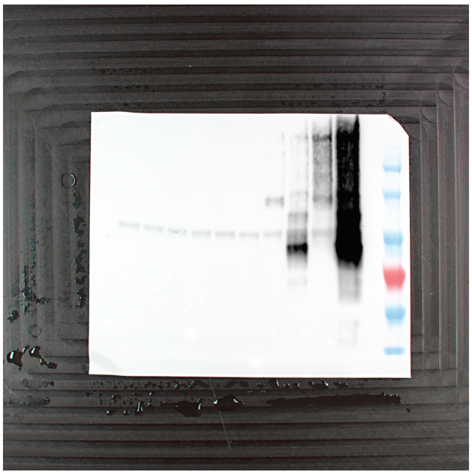

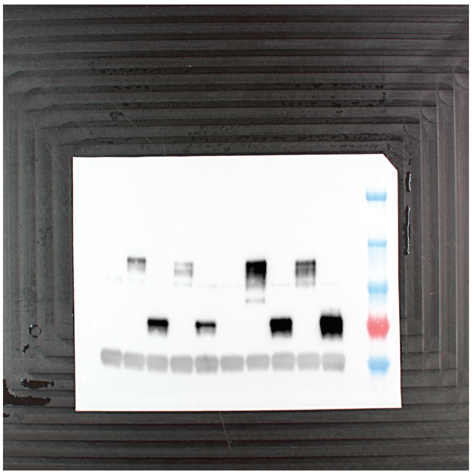

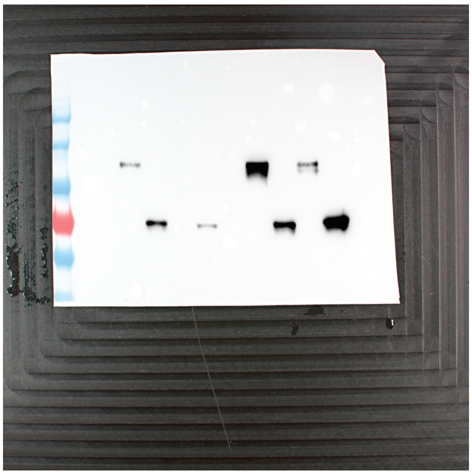

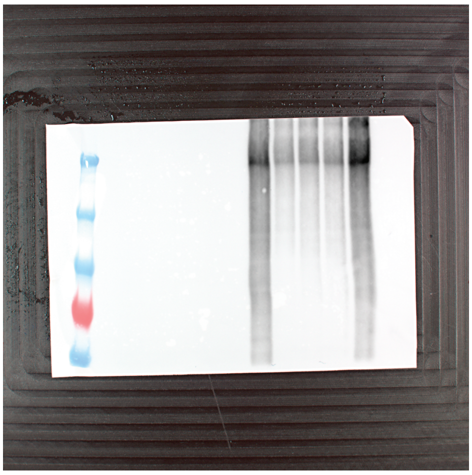

Fig6A

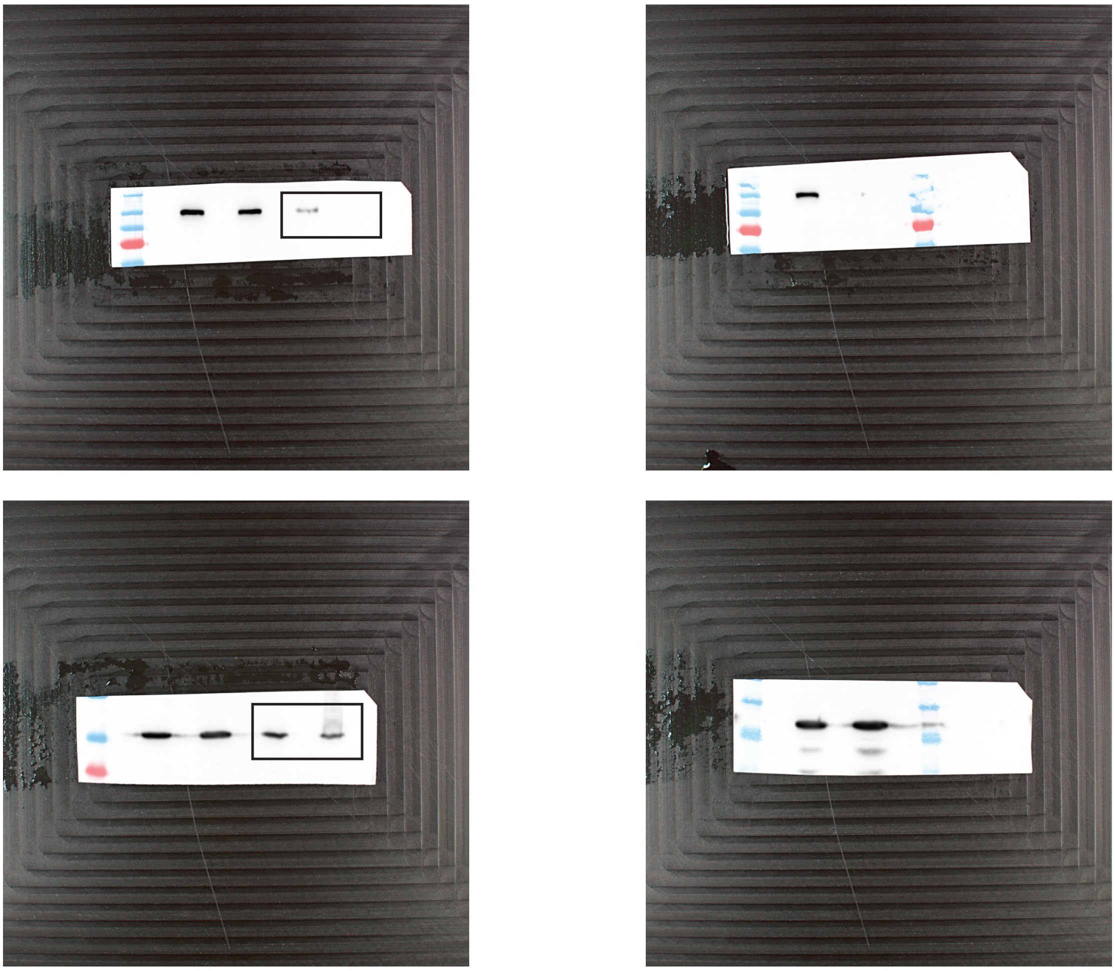

Fig6B

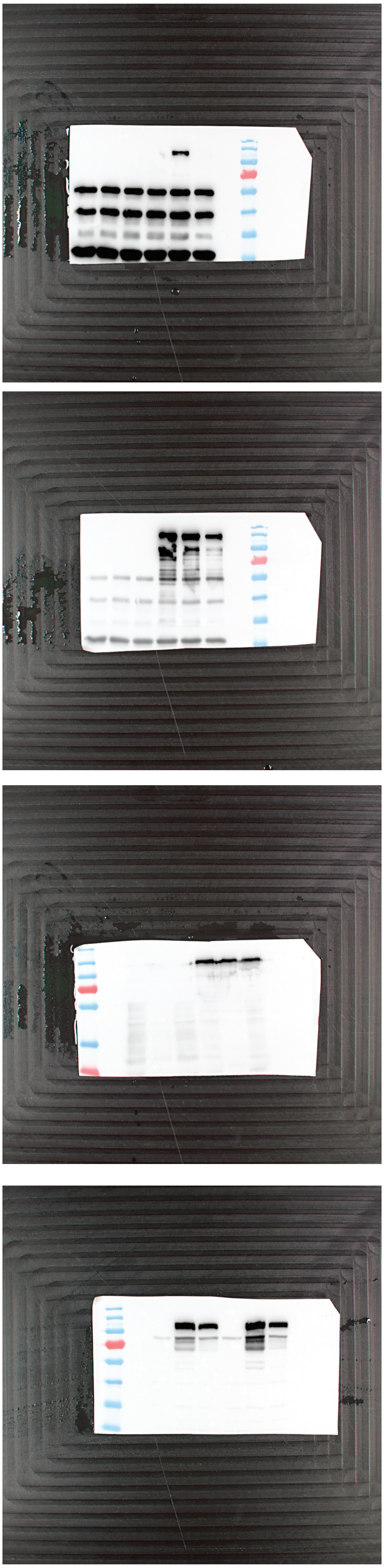

Fig6C

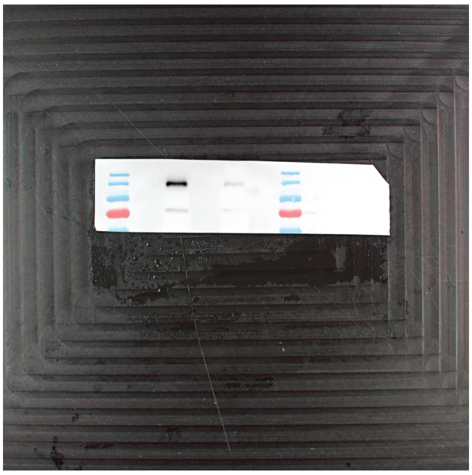

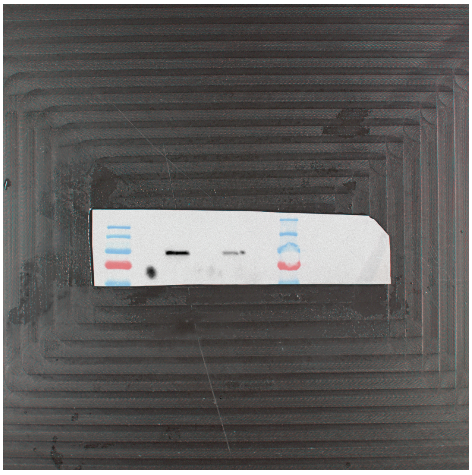

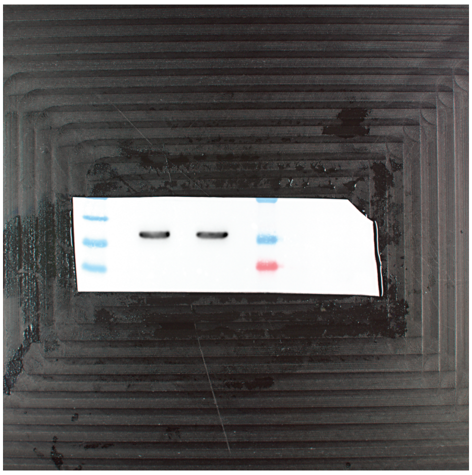

Fig. S1 A

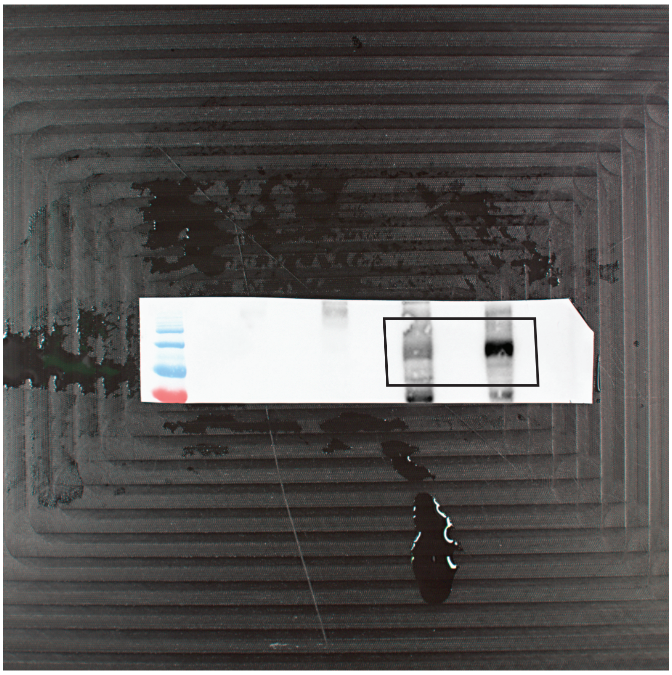

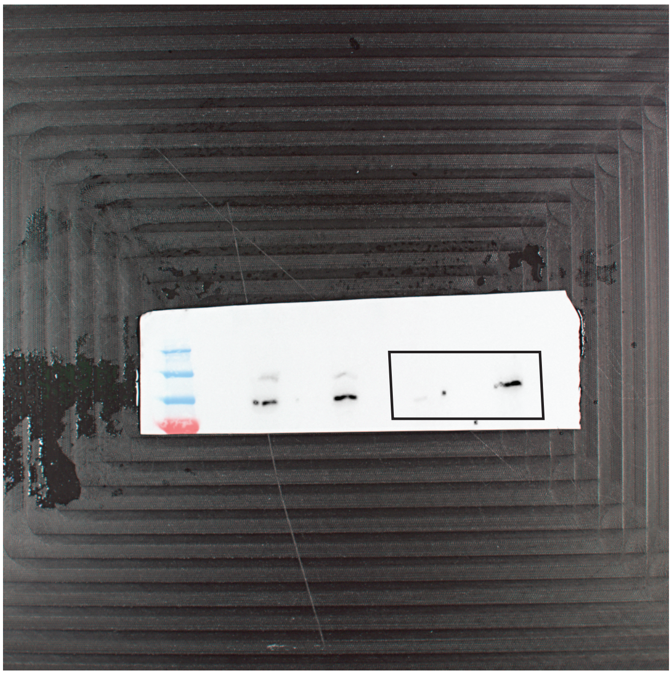

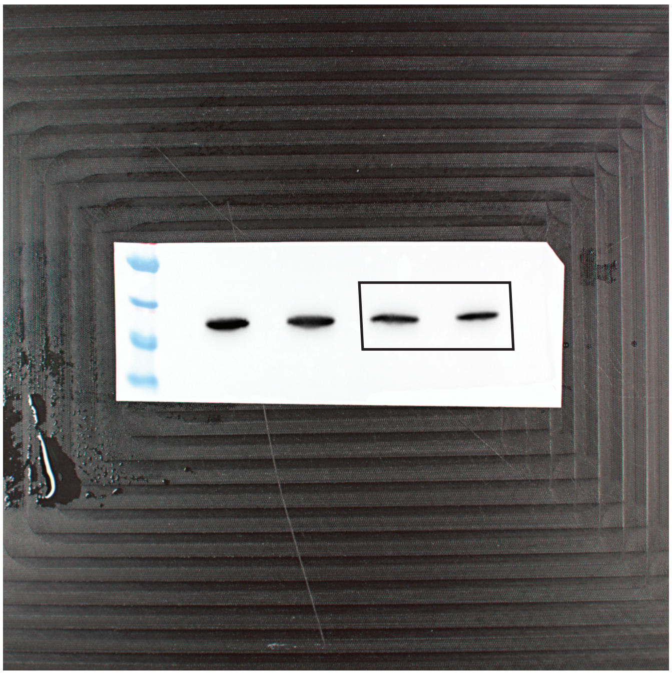

Fig. S1 B

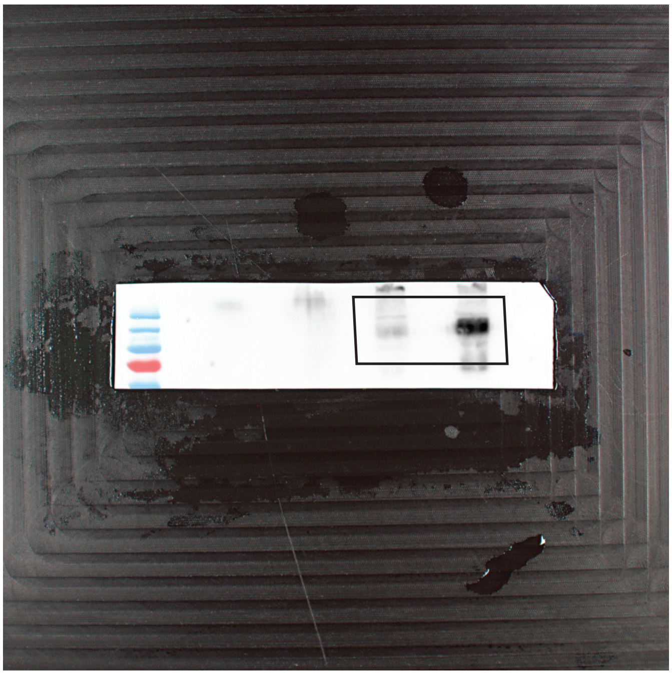

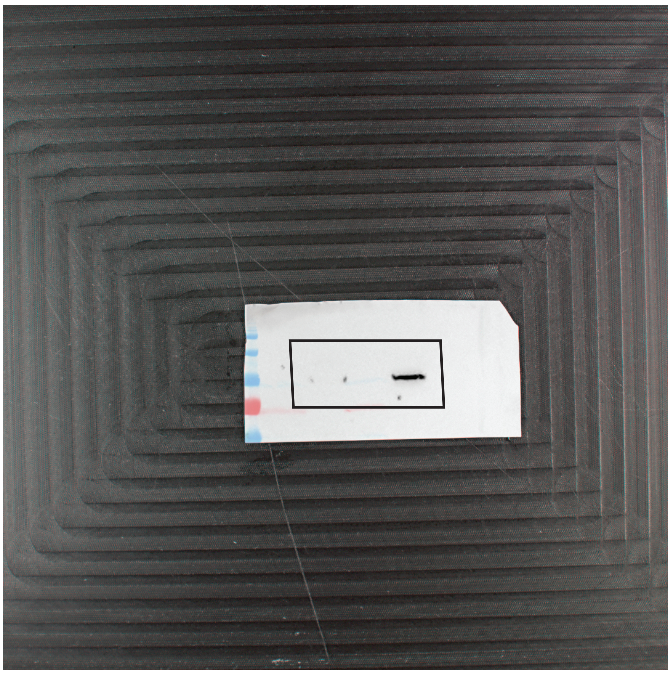

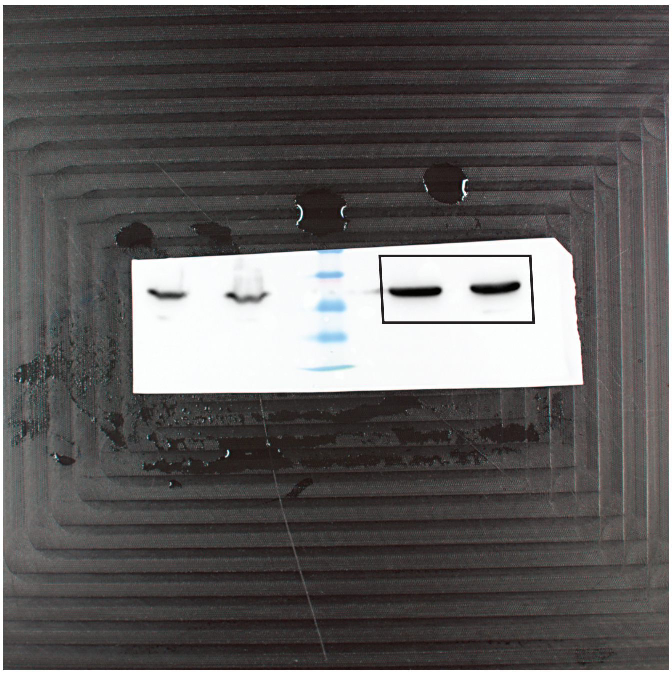

FigS4A

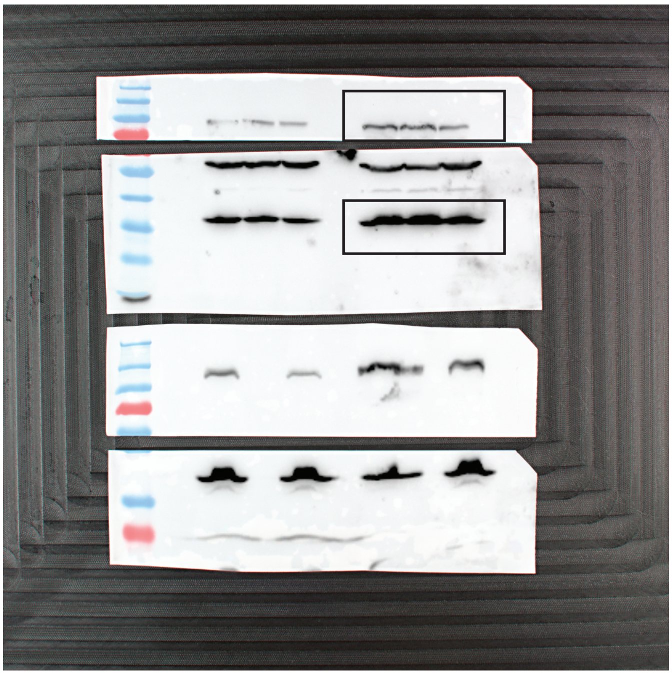

FigS4B

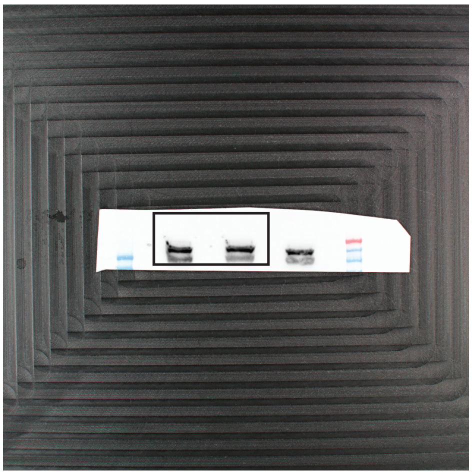

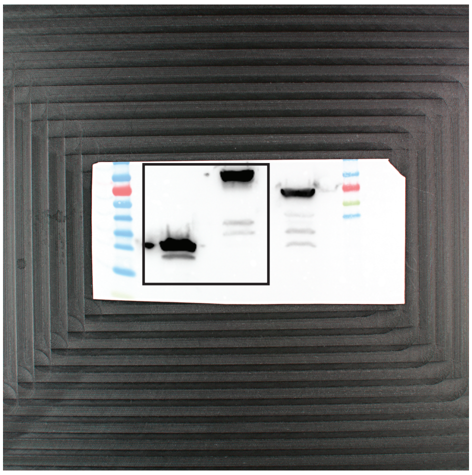

FigS4C

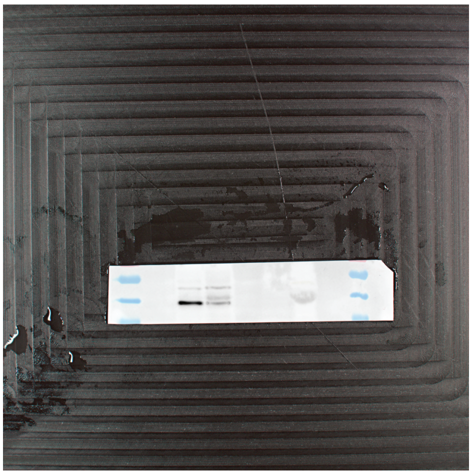

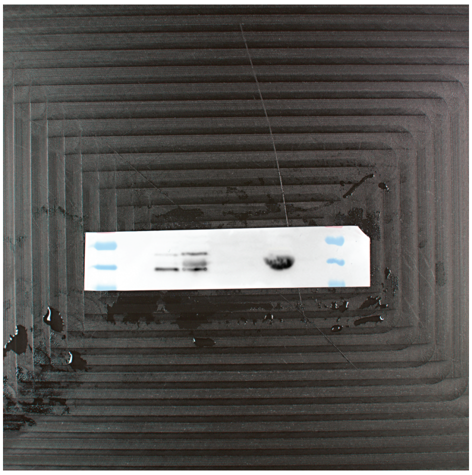

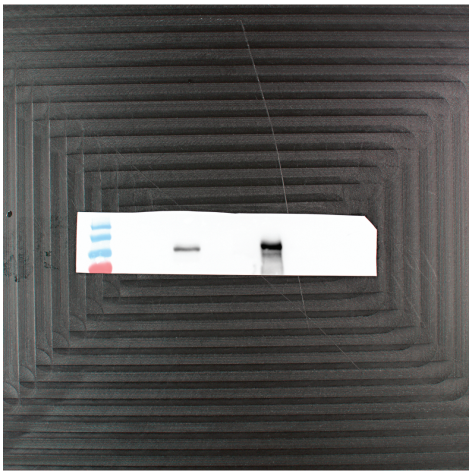

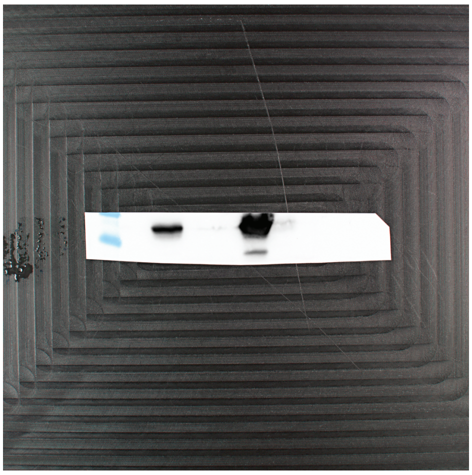

FigS4continued

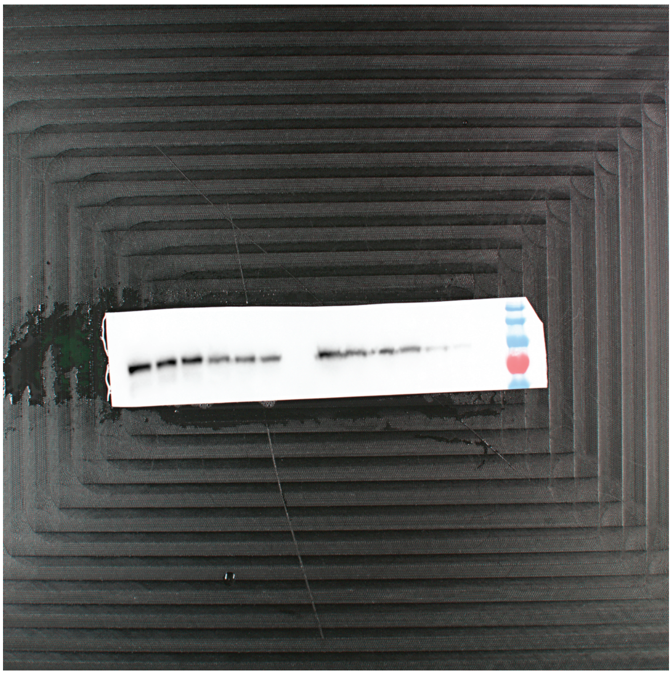

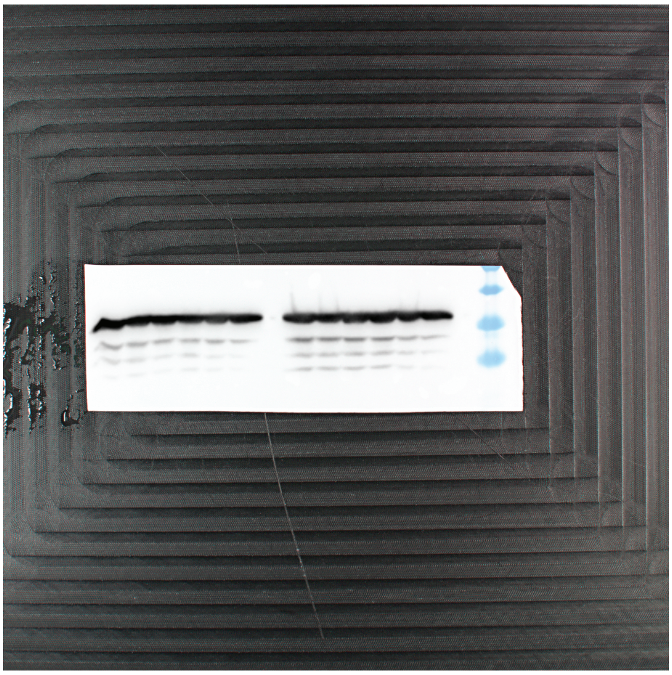

FigS4E

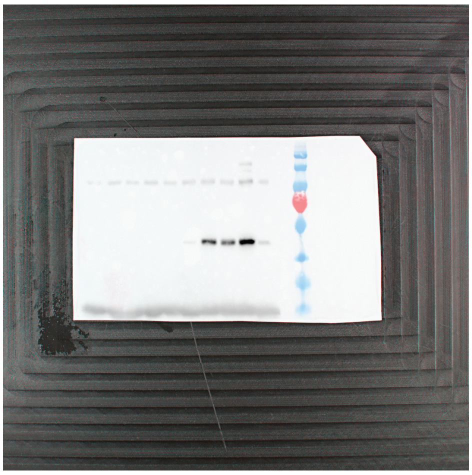

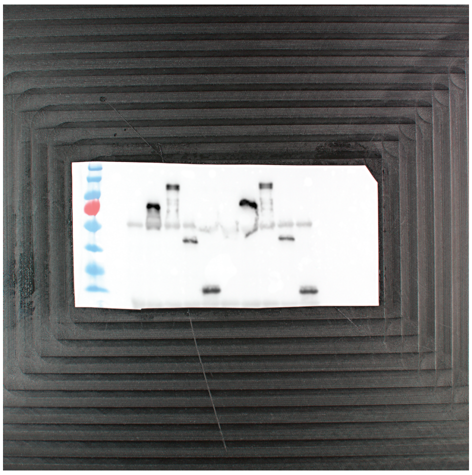

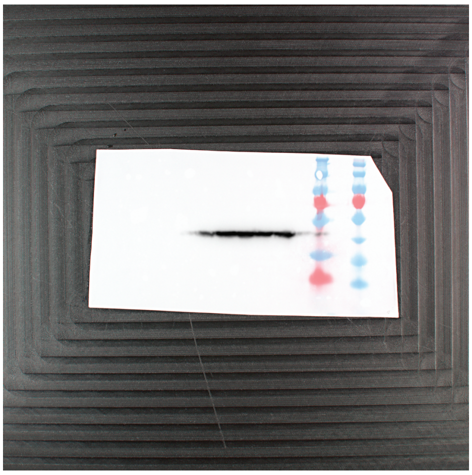

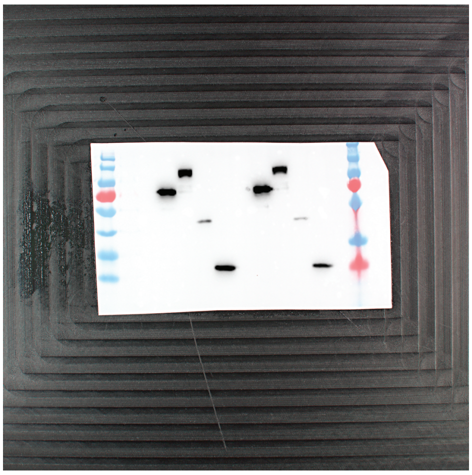

FigS5A

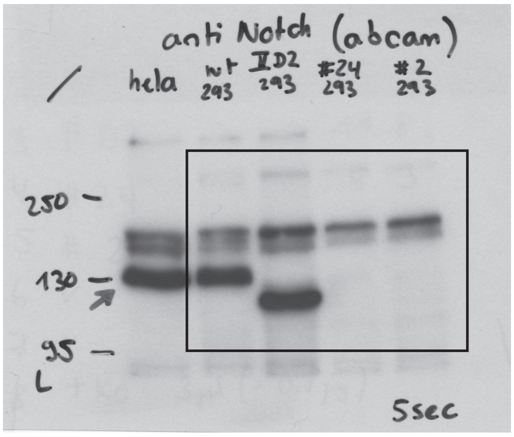

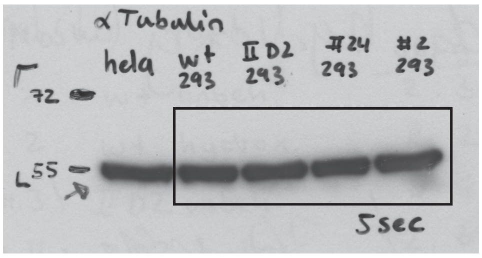

FigS5B

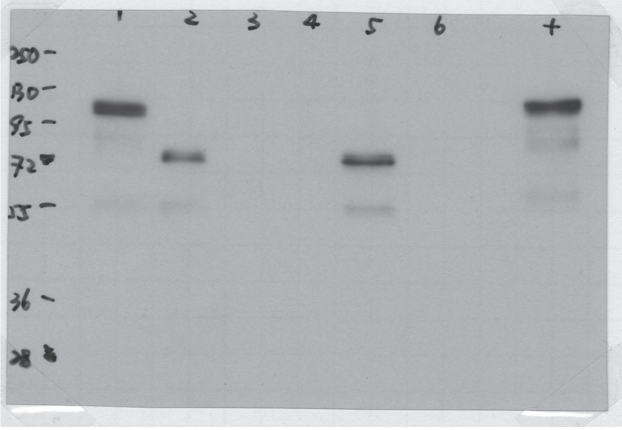

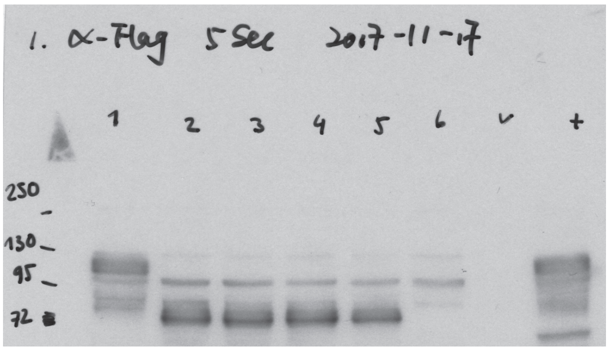

FigS5C

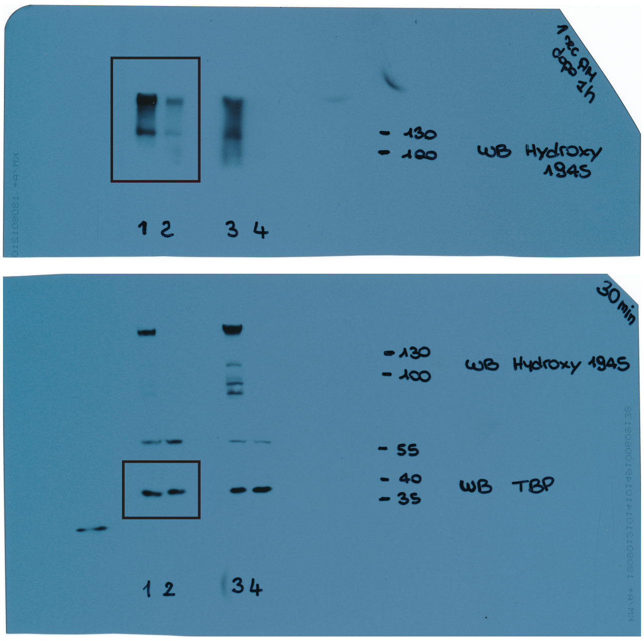

FigS5D

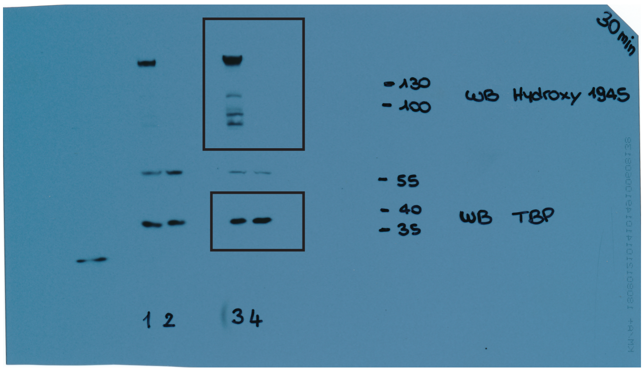

FigS5E

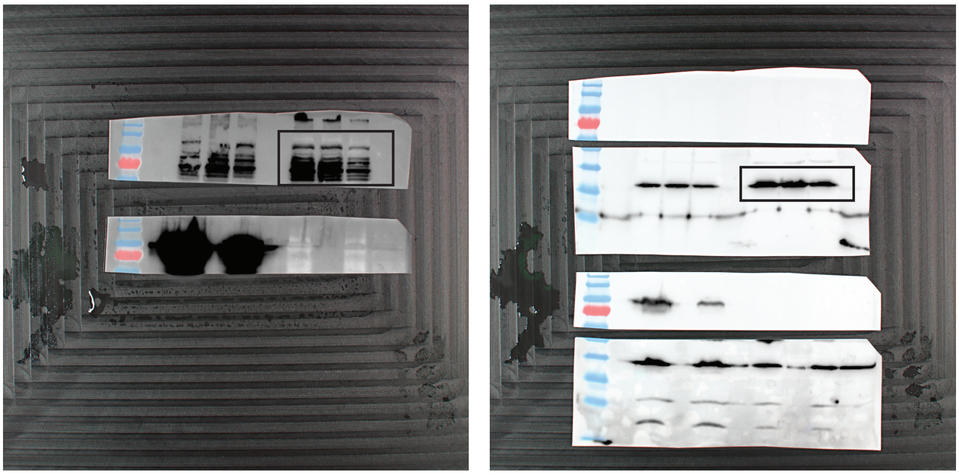

FigS6C

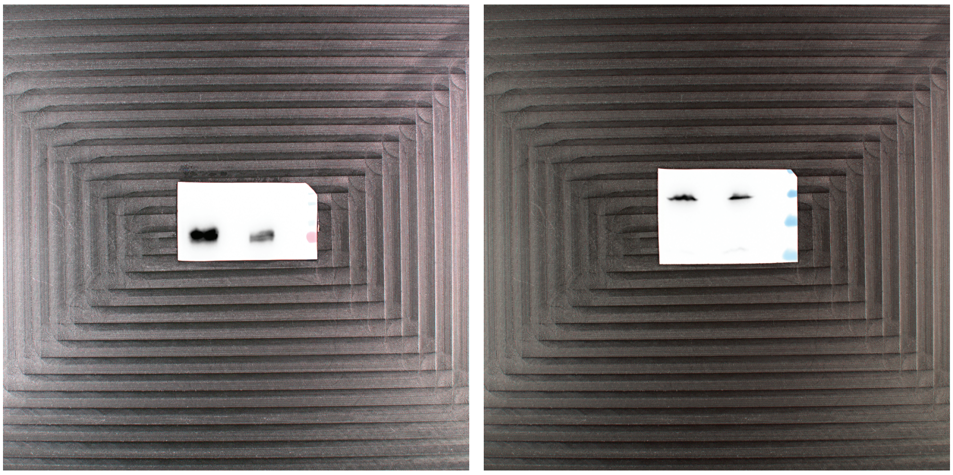

FigS6D

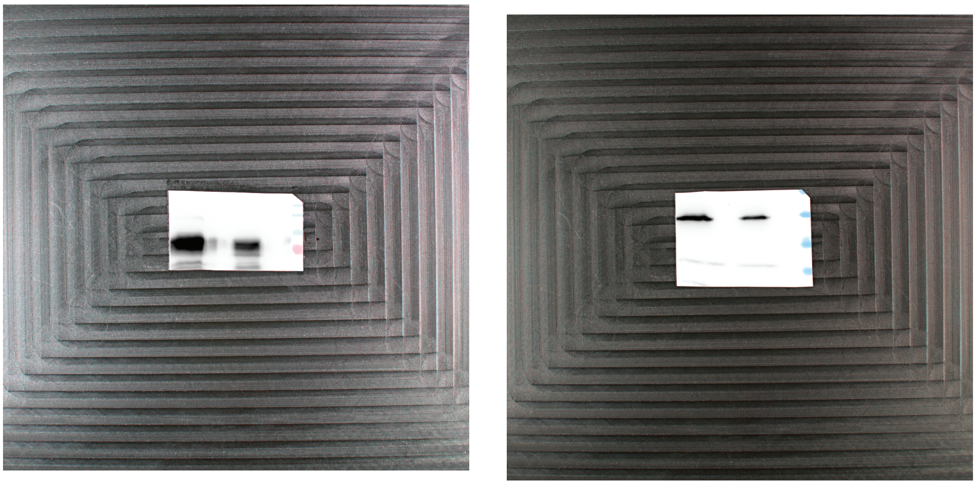

FigS6E

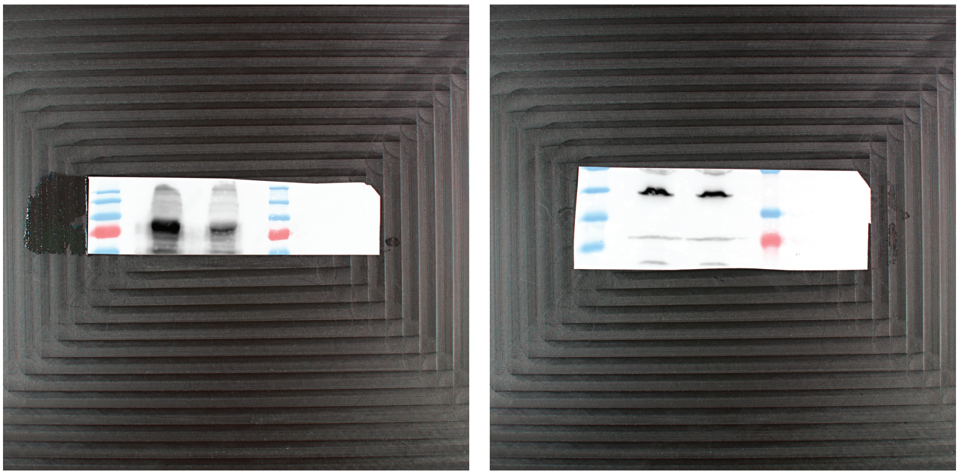

FigS6F

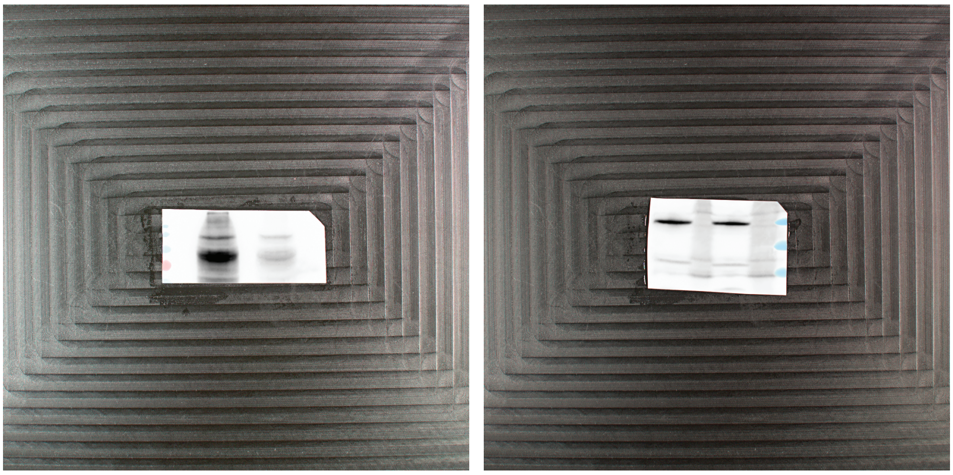

FigS7B

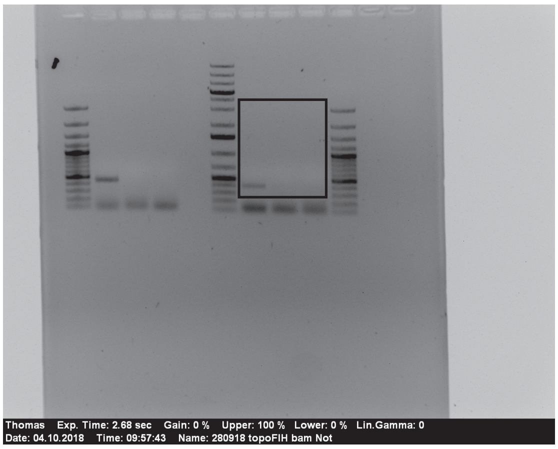

FigS7C

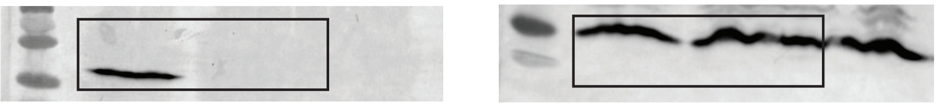

FigS7D

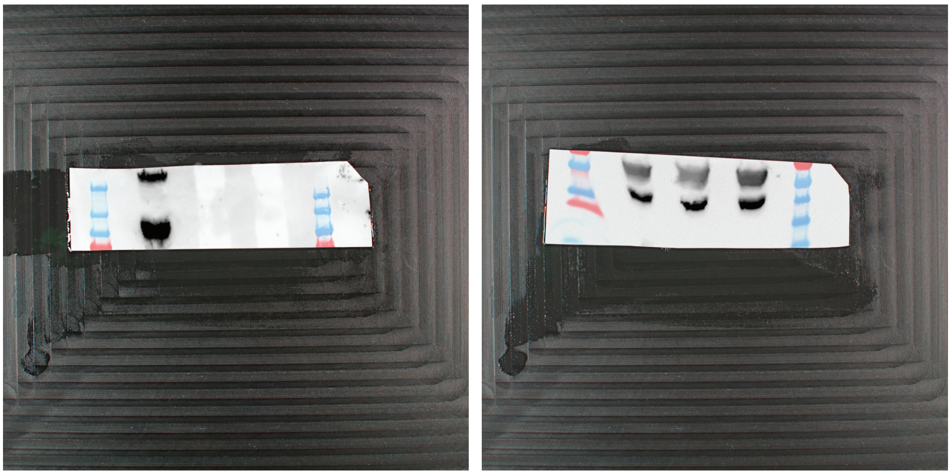

FigS7E

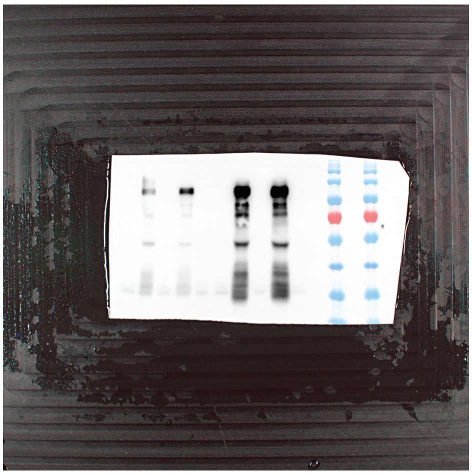

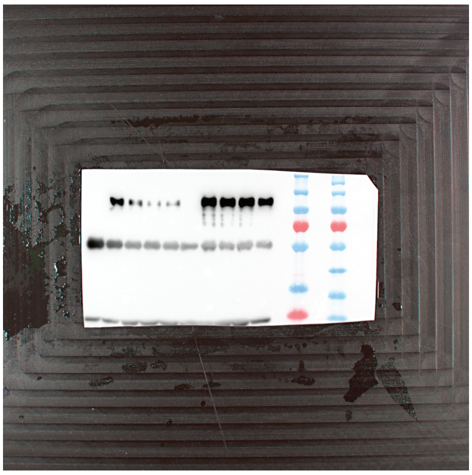

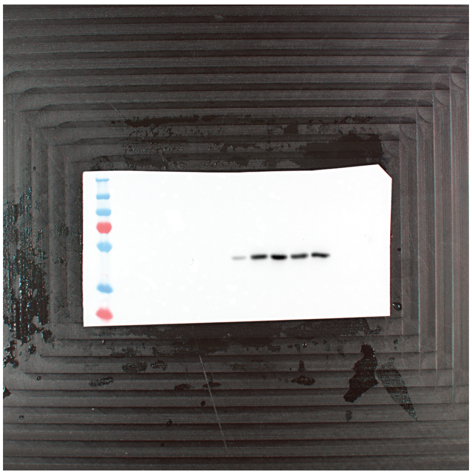

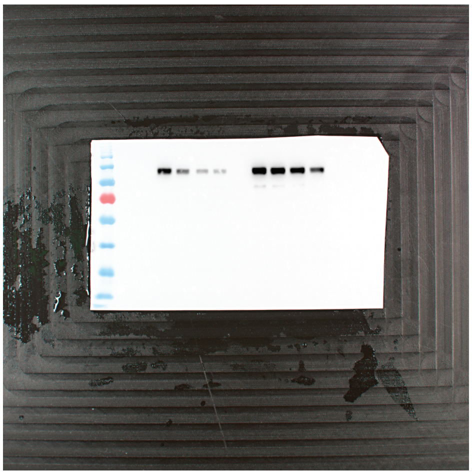

FigS8A

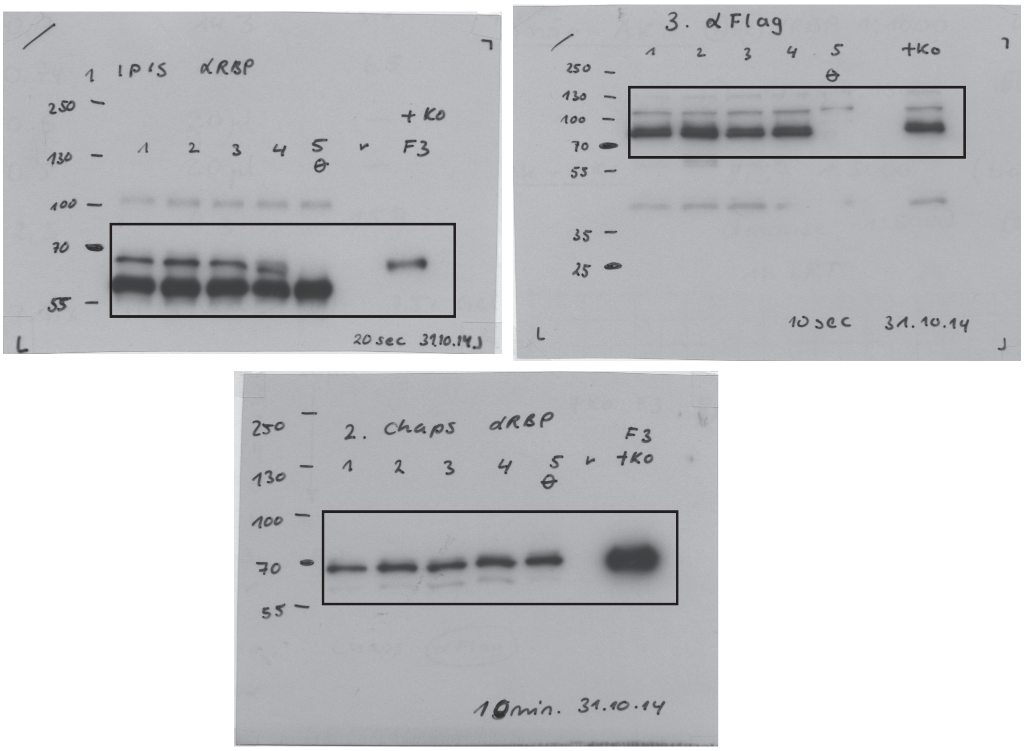

FigS8B

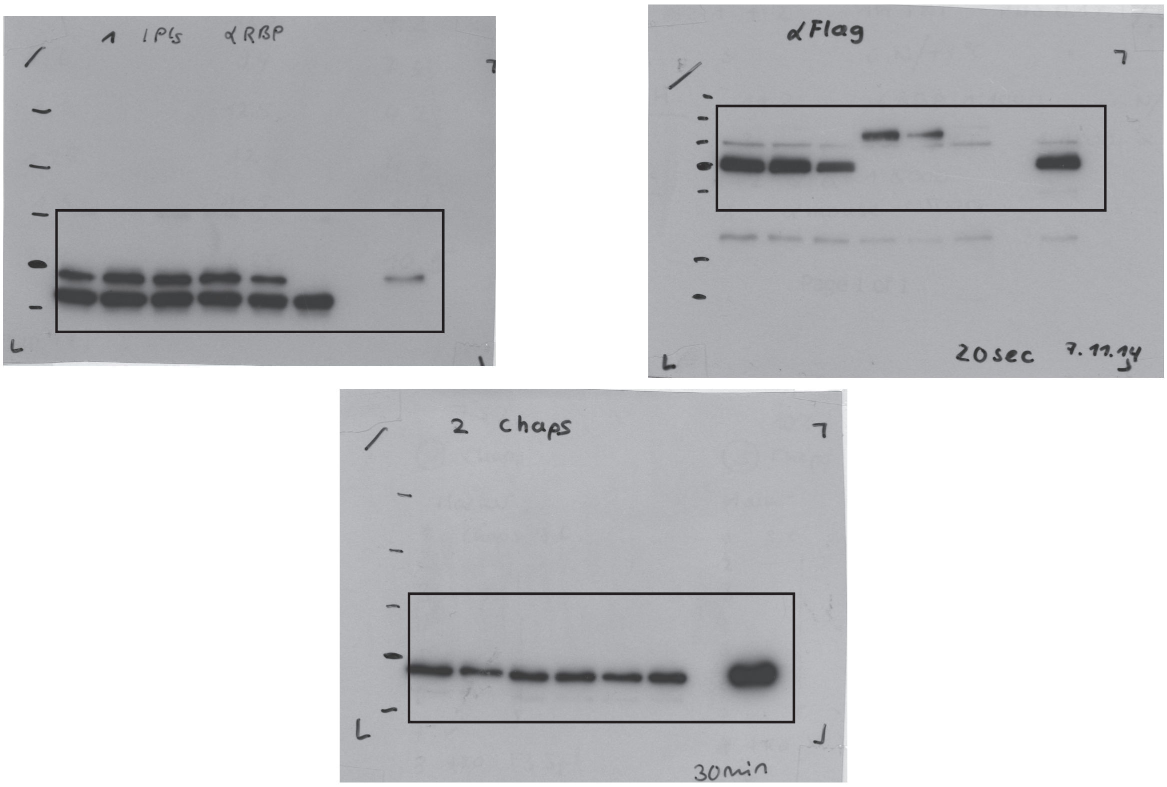

FigS8C

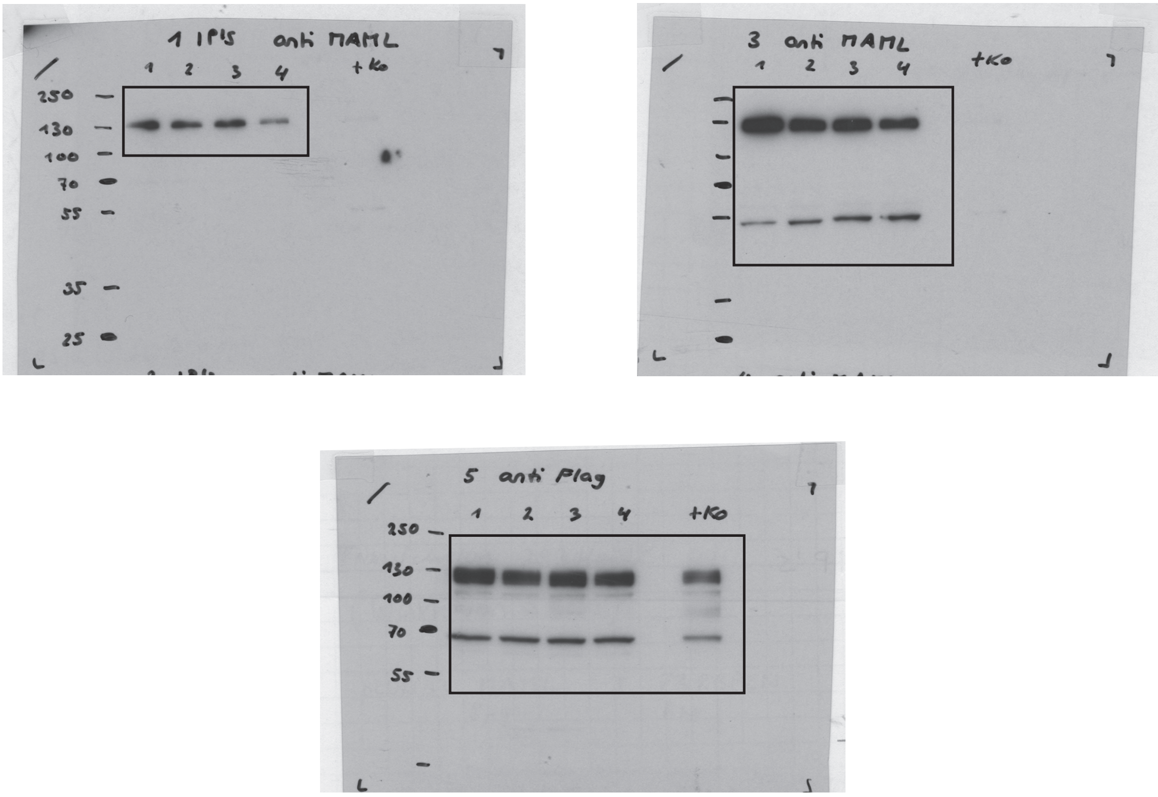

FigS8D

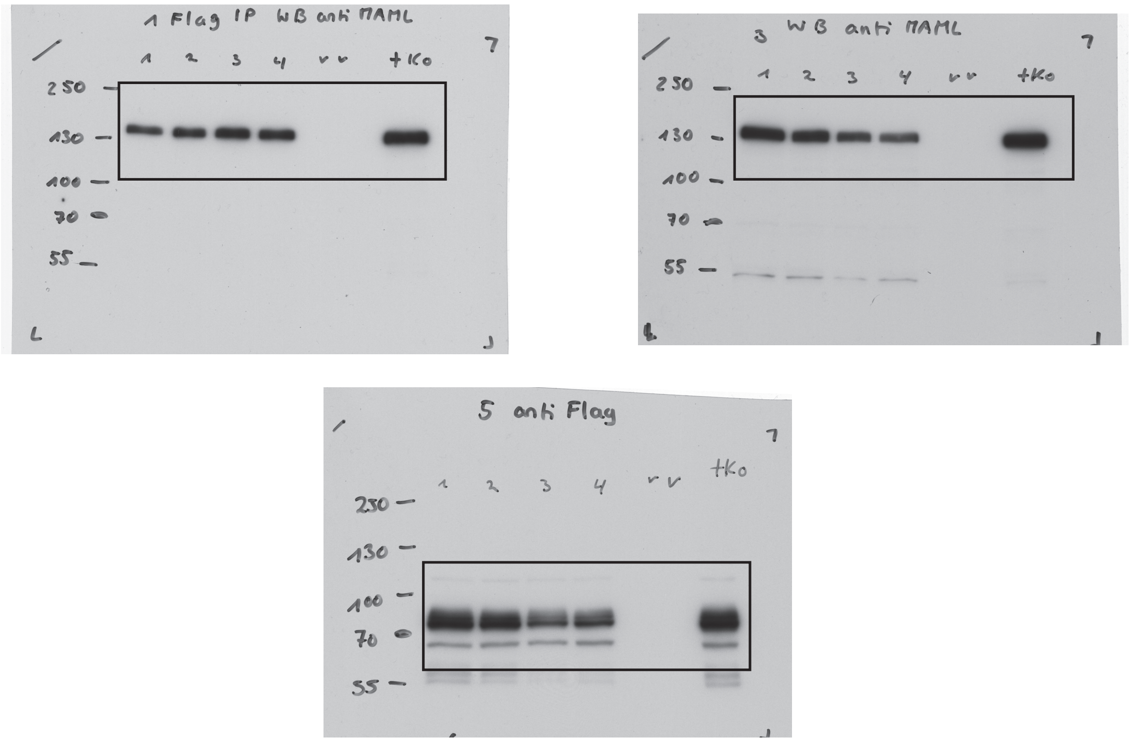

FigS9A

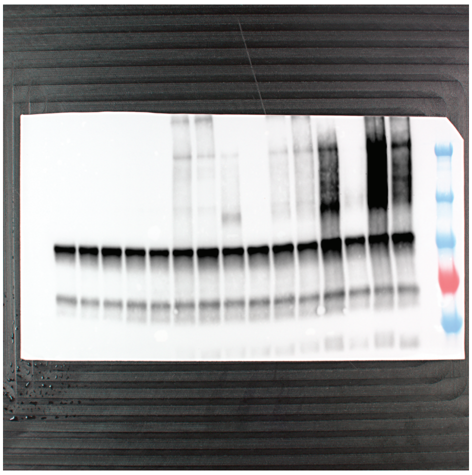

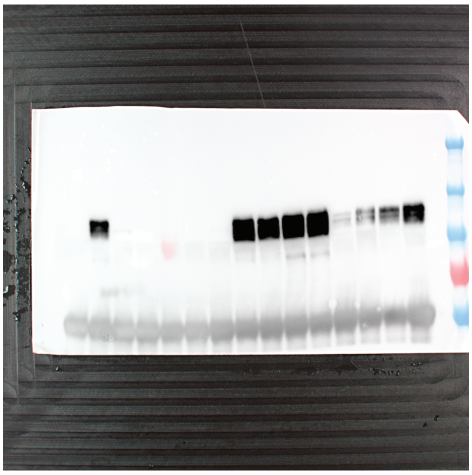

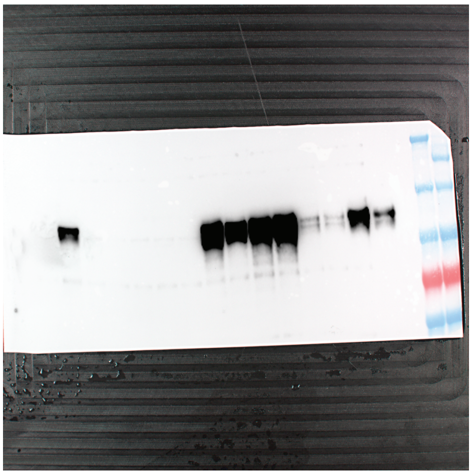

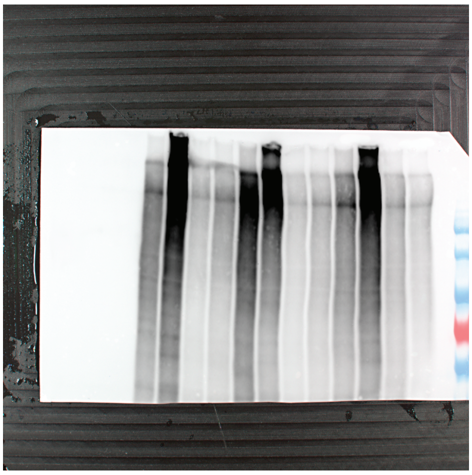

FigS9B

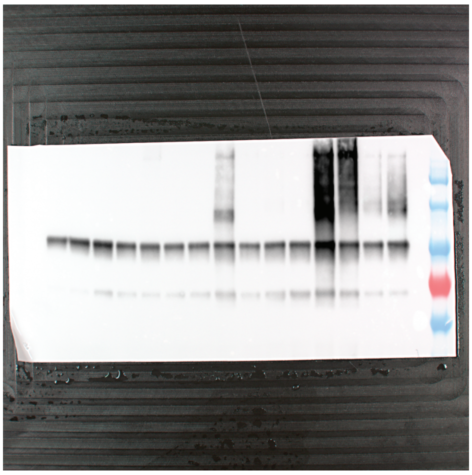

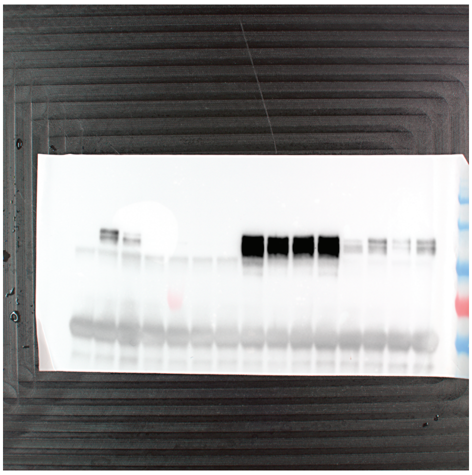

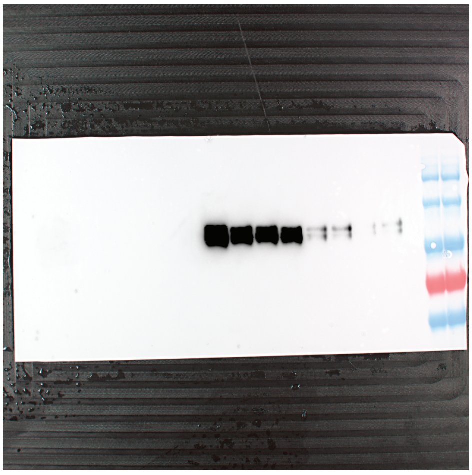

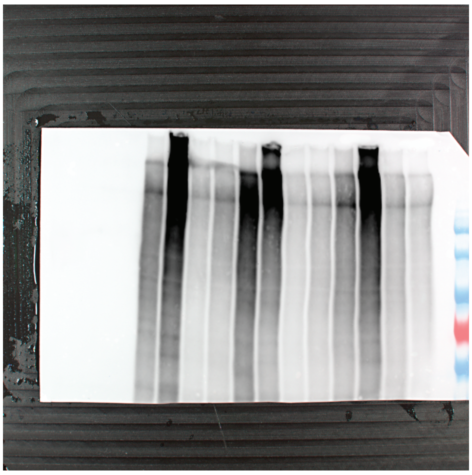

FigS10A

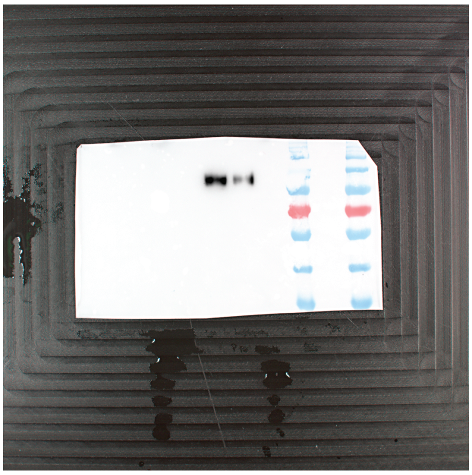

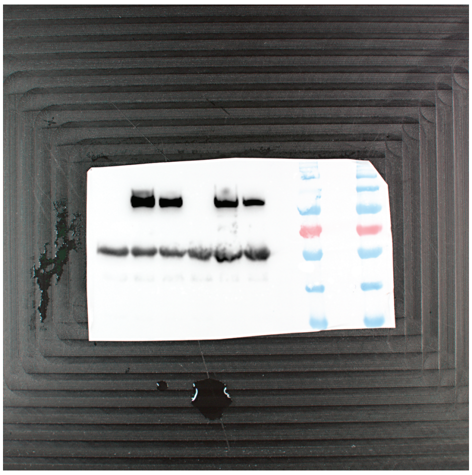

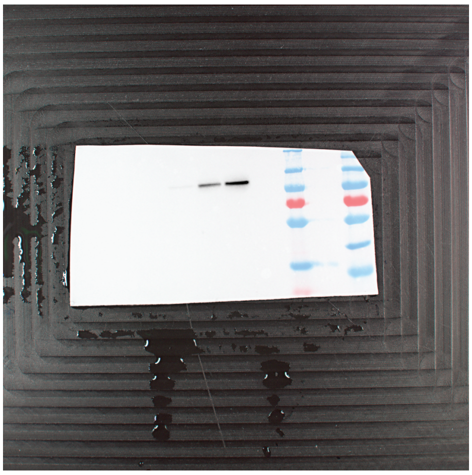

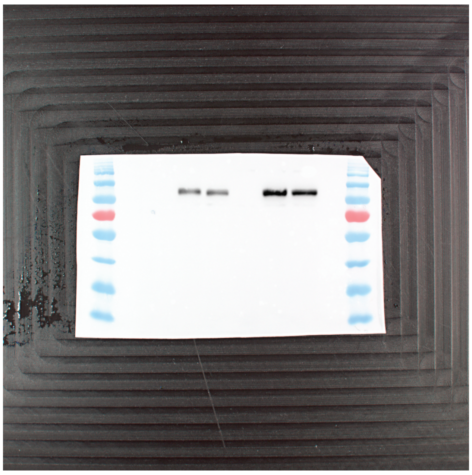

FigS10B

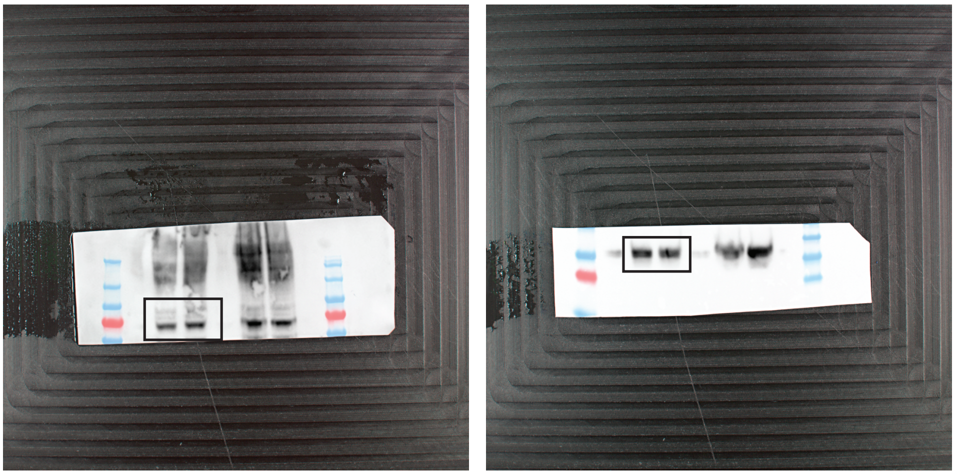

FigS10C

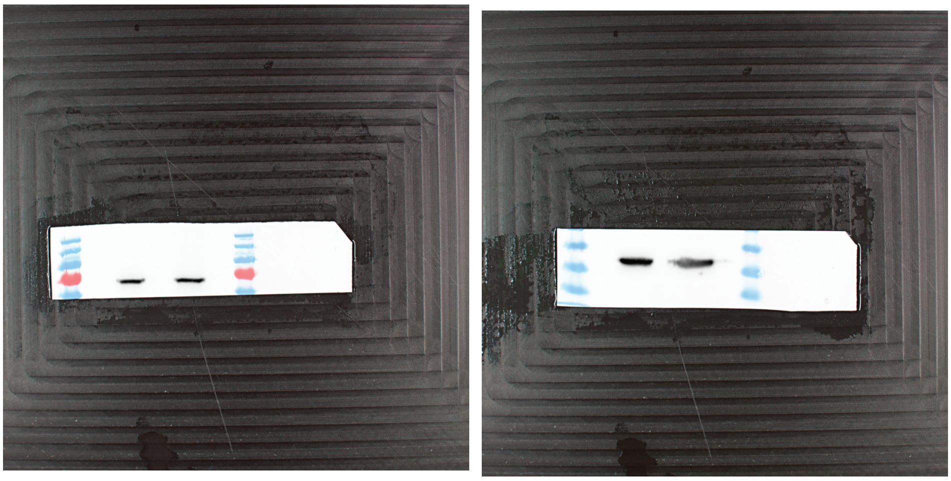

Supplement: Supplementary file 27 — original blots [file 41419_2022_5052_MOESM27_ESM.pdf]
